# Genetic Basis for Spontaneous Hybrid Genome Doubling during Allopolyploid Speciation of Common Wheat Shown by Natural Variation Analyses of the Paternal Species

**DOI:** 10.1371/journal.pone.0068310

**Published:** 2013-08-08

**Authors:** Yoshihiro Matsuoka, Shuhei Nasuda, Yasuyo Ashida, Miyuki Nitta, Hisashi Tsujimoto, Shigeo Takumi, Taihachi Kawahara

**Affiliations:** 1 Fukui Prefectural University, Matsuoka, Eiheiji, Yoshida, Fukui, Japan; 2 Laboratory of Plant Genetics, Graduate School of Agriculture, Kyoto University, Kitashirakawaoiwake-cho, Sakyo-ku, Kyoto, Japan; 3 Laboratory of Molecular Breeding, Arid Land Research Center, Tottori University, Tottori-shi, Tottori, Japan; 4 Laboratory of Plant Genetics, Graduate School of Agricultural Science, Kobe University, Nada-ku, Kobe, Japan; 5 Laboratory of Crop Evolution, Plant Germ-plasm Institute, Graduate School of Agriculture, Kyoto University, Mozume, Muko, Kyoto, Japan; Kansas State University, United States of America

## Abstract

The complex process of allopolyploid speciation includes various mechanisms ranging from species crosses and hybrid genome doubling to genome alterations and the establishment of new allopolyploids as persisting natural entities. Currently, little is known about the genetic mechanisms that underlie hybrid genome doubling, despite the fact that natural allopolyploid formation is highly dependent on this phenomenon. We examined the genetic basis for the spontaneous genome doubling of triploid F_1_ hybrids between the direct ancestors of allohexaploid common wheat (*Triticum aestivum* L., AABBDD genome), namely *Triticum* *turgidum* L. (AABB genome) and *Aegilops* *tauschii* Coss. (DD genome). An Ae. *tauschii* intraspecific lineage that is closely related to the D genome of common wheat was identified by population-based analysis. Two representative accessions, one that produces a high-genome-doubling-frequency hybrid when crossed with a *T* *. turgidum* cultivar and the other that produces a low-genome-doubling-frequency hybrid with the same cultivar, were chosen from that lineage for further analyses. A series of investigations including fertility analysis, immunostaining, and quantitative trait locus (QTL) analysis showed that (1) production of functional unreduced gametes through nonreductional meiosis is an early step key to successful hybrid genome doubling, (2) first division restitution is one of the cytological mechanisms that cause meiotic nonreduction during the production of functional male unreduced gametes, and (3) six QTLs in the *Ae* *. tauschii* genome, most of which likely regulate nonreductional meiosis and its subsequent gamete production processes, are involved in hybrid genome doubling. Interlineage comparisons of *Ae* *. tauschii* ’s ability to cause hybrid genome doubling suggested an evolutionary model for the natural variation pattern of the trait in which non-deleterious mutations in six QTLs may have important roles. The findings of this study demonstrated that the genetic mechanisms for hybrid genome doubling could be studied based on the intrinsic natural variation that exists in the parental species.

## Introduction

Allopolyploidy, i.e., polyploid formation via interspecific hybridization and subsequent genome doubling, is an important mode of speciation in plants. Allopolyploid formation, and ultimately speciation, is initiated by a species cross that produces interspecific F_1_ hybrids. Because the chromosomes inherited from the diverged parents are unable to pair with each other in meiosis, such hybrids usually have very low fertility, but may undergo genome doubling through somatic doubling, through union of unreduced gametes, or by means of a triploid bridge [1]. As a result of hybrid genome doubling, a new allopolyploid is formed with a complete set of somatic chromosomes from the parental species merged in a common nucleus. Newly formed allopolyploids may have, in addition to improved fertility, genetic and ecological advantages such as extreme phenotypic traits and reproductive isolation that provide avenues for exploiting the peaks in an adaptive landscape.

Allopolyploid speciation is a complex process that includes a variety of mechanisms ranging from species crosses and viable hybrid formation to hybrid genome doubling, genome alterations and the establishment of new allopolyploids as persisting entities in ecosystems. Owing mainly to the development of modern molecular techniques, recent studies have revealed novel details about genome alterations, including rapid genetic, epigenetic, and genomic changes, with which hybridization and allopolyploid genome formation are accompanied [2]. For the other mechanisms, however, several fundamental questions remain to be addressed. Particularly, very little progress has been made so far in understanding the genetic mechanisms that underlie hybrid genome doubling, even though the occurrence of natural allopolyploid formation is highly dependent on this phenomenon.

Direct ancestors of common wheat (*Triticum aestivum* L.), *Triticum* *turgidim* L. (AABB genome) and *Aegilops* *tauschii* Coss. (formerly known as *Aegilops squarrosa* L.) (DD genome), provide suitable materials for studying the genetic mechanisms for hybrid genome doubling. *T* *. turgidum* is one of the “founder grain crops” that were domesticated in the Fertile Crescent about 10,000 years ago, whereas *Ae* *. tauschii* is a selfing species that is widely distributed in central Eurasia [3]. The center of *Ae* *. tauschii* ’s current distribution is in the southern coastal region of the Caspian Sea and Azerbaijan, whereas the natural habitats spread from the center eastward to western China via the Kopet Dag Mountains of Turkmenistan and westward to central Syria via the valleys of mountainous southeastern Turkey [4]. Allohexaploid common wheat (AABBDD genome) originated in the Middle East/Transcaucasus region ca. 8,000 years ago and is derived from a natural hybrid cross between a cultivated form of *T* *. turgidum* (female parent) and the wild species *Ae* *. tauschii* (male parent) [5,6]. By making artificial crosses between *T* *. turgidum* and *Ae* *. tauschii* , an essential part of natural *T. aestivum* formation can be reproduced using neither chemicals nor embryo rescue techniques. Through such crosses, triploid F_1_ hybrids (ABD genome) that spontaneously undergo genome doubling by setting hexaploid seeds (AABBDD genome) via union of unreduced gametes can be obtained [7–9]. In wheat, therefore, an occurrence of hybrid genome doubling is detectable as a selfed seed set of the triploid F_1_ hybrids. Artificial crosses can also provide such hybrids that display various postzygotic barriers, i.e., such abnormalities as severe dwarfness and necrotic dysgenesis [10–13].

Since the report of unreduced gametes forming in a *T* *. turgidum* - *Ae* *. tauschii* F_1_ hybrid that underwent spontaneous genome doubling [14], the genetic underpinning of the *T* *. turgidum* - *Ae* *. tauschii* hybrid genome doubling has extensively been studied by the use of artificial hybrids derived from various parental genotypes. In the *T* *. turgidum* - *Ae* *. tauschii* F_1_ hybrids, functional male and female gametes are produced by nonreductional meiosis that generates unreduced gametes [7,8,15]. Those studies reported that (1) the hybrids produce unreduced gametes through a meiotic process that includes single cell division rather than two consective divisions, and (2) the occurrence of hybrid genome doubling is genetically controlled [7,8,15–22]. In addition, a previous artificial-cross study that used a *T* *. turgidum* cultivar as the tester showed the geographic patterns of cryptic natural variation for fertile triploid F_1_ hybrid formation in *Ae* *. tauschii* [11]. When crossed with a *T* *. turgidum* accession, some *Ae* *. tauschii* accessions produced high-genome-doubling-frequency hybrids (HGD hybrids; selfed seed set rate > 0.5), others produced low-genome-doubling-frequency hybrids (LGD hybrids; selfed seed set rate < ca. 0.1), whereas many accessions produced triploid F_1_ hybrids that have intermediate genome doubling frequencies (selfed seed set rates ranging from 0.1 to 0.5). The geographic and chloroplast-DNA-based genealogical structures of this intraspecific variation, however, are not evident. Interestingly, the *Ae* *. tauschii* accessions that gave rise to hexaploid-seed-setting hybrids have a wide geographic distribution, whereas those that caused hybrid abnormality are restricted to particular regions. Furthermore, the triploid F_1_ hybrids showed extensive variation in the selfed seed set rates (7.5-68.3%) depending on the genotype of the *Ae* *. tauschii* accessions, suggesting that, in those hybrids, the occurrence of genome doubling is under some sort of genetic control. These results underscored the potential of *Ae* *. tauschii* natural accessions for studying the genetic mechanisms that underlie hybrid genome doubling and invited further work. Unanswered questions regarding the hybrid genome doubling that occurred in the allopolyploid speciation of common wheat include: (1) whether or not some intraspecific lineages of the ancestors are more capable of producing hexaploid-seed-setting F_1_ hybrids than others; (2) what cytological mechanisms underlie the hybrid genome doubling; and (3) how many and what kind of genes are involved in hybrid genome doubling in the *T* *. turgidum* - *Ae* *. tauschii* system. Obviously, these questions have direct relevance to understanding the complex process of allopolyploid speciation in plants.

In this paper, we addressed these questions with the use of *Ae* *. tauschii* ’s natural variation regarding hybrid genome doubling. For the purpose of the study, we first analyzed the population structure of *Ae* *. tauschii* and identified the lineage that is genetically closely related to the D genome of common wheat. We then performed a series of genetic and cytological analyses using two representative accessions of *Ae* *. tauschii* lineages, one accession that produces an HGD hybrid when crossed with a *T* *. turgidum* cultivar ‘Langdon’ (LDN) and another accession that produces an LGD hybrid with LDN. The findings of this study demonstrated that the genetic mechanisms for hybrid genome doubling in allopolyploid speciation could be studied based on the intrinsic natural variation that exists in the parental species and provided novel insights into the evolution of common wheat.

## Results

### *Ae* *. tauschii* intraspecific lineages and their relationships to the D genome of common wheat

**Figure 1:**
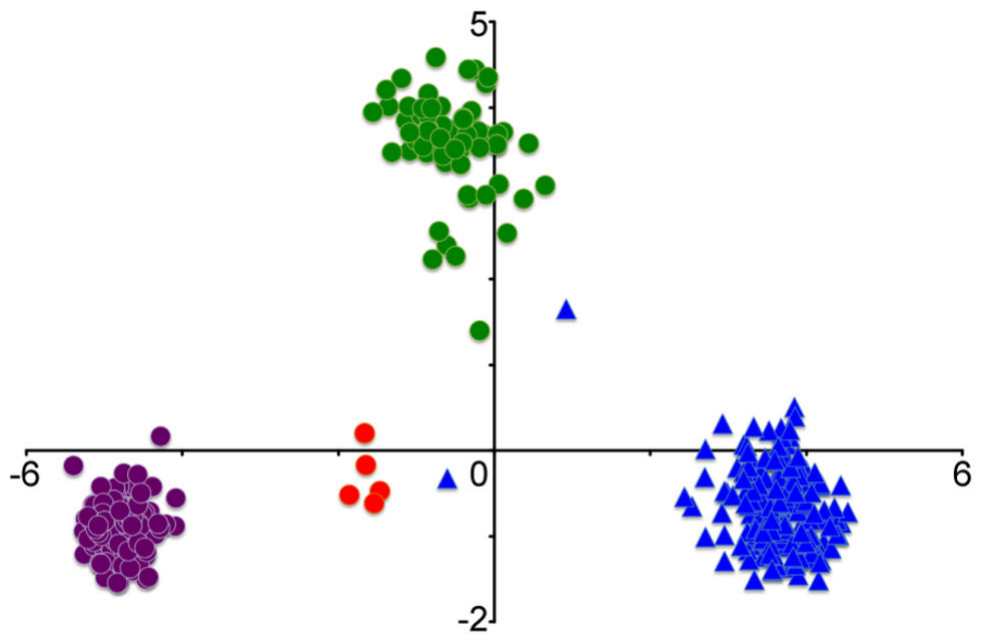
Graph of the first two axes from a PCA based on DArT marker variations. The first component (x) accounts for 41.8% and the second (y) for 8.5% of the total variance. Circles denote *Ae* *. tauschii* TauL1 (purple), TauL2 (green), and TauL3 (red), and triangles *T. aestivum*.

To identify the *Ae* *. tauschii* intraspecific lineage that is most closely related to the D genome of common wheat, population-level analyses were conducted using a Diversity Arrays Technology marker genotype dataset for 206 *Ae* *. tauschii* accessions that represented the entire species range and a diverse array of 188 common wheat accessions consisting of traditional and modern cultivars of the East and West and one synthetic wheat line W7984 (Table S1). In a previous amplified-fragment-length-polymorphism study that used a portion of the 206 *Ae* *. tauschii* accession set, three distinct intraspecific lineages were found [23]. Consistent with that result, a principal component analysis (PCA) of the DArT dataset showed that the 206 *Ae* *. tauschii* accessions are grouped into the same three intraspecific lineages: TauL1 (renamed from L1), TauL2 (renamed from L2), and TauL3 (renamed from HGL17) (Figure 1, Table S2). Geographically, the TauL1 accessions are widely spread across the species range, whereas the TauL2 and TauL3 accessions are restricted to the Transcaucasus/Middle East region and Georgia, respectively (Figure S1). In contrast, almost all common wheat accessions formed a single isolated cluster that was more closely associated with TauL2 and TauL3 than TauL1. Two common wheat accessions were exceptionally closely associated with *Ae* *. tauschii* : the synthetic wheat line W7984 (placed near TauL2) and an Ethiopian landrace KU-9873 (placed near TauL3). The reason for the KU-9873-*Ae. tauschii* association was not clear. Separation of the D genome of common wheat from *Ae* *. tauschii* was fully consistent with the results of restriction-fragment-length-polymorphism, microsatellite, and single-nucleotide-polymorphism studies [24–26].

**Figure 2:**
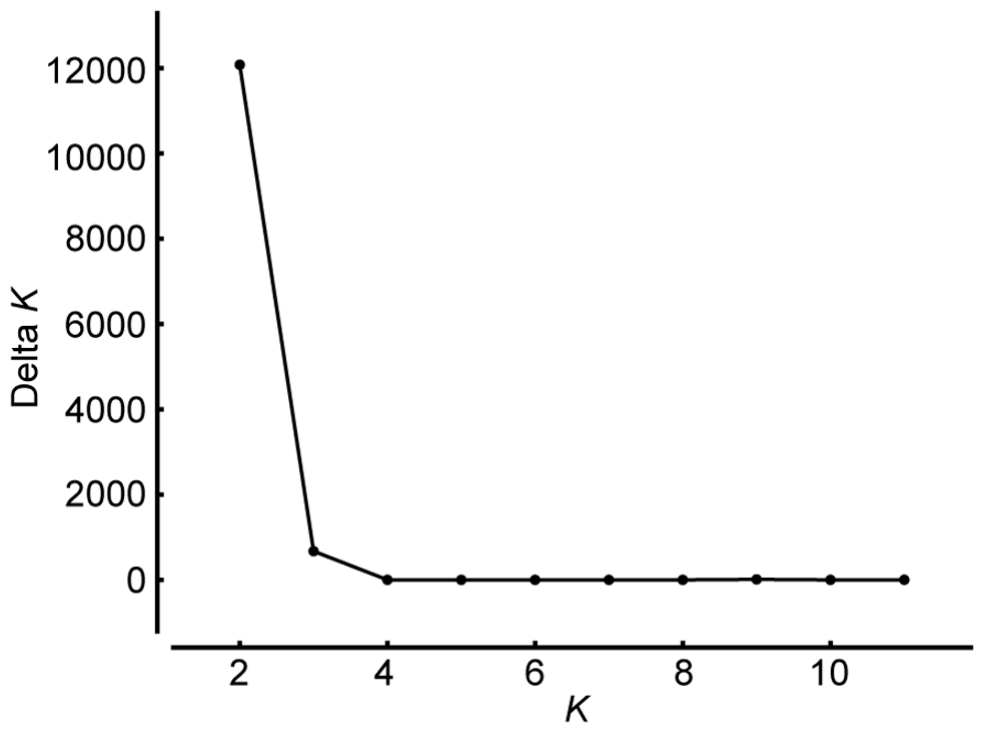
Plot of ∆*K* from the STRUCTURE analysis of the full set of sample accessions.

**Figure 3:**
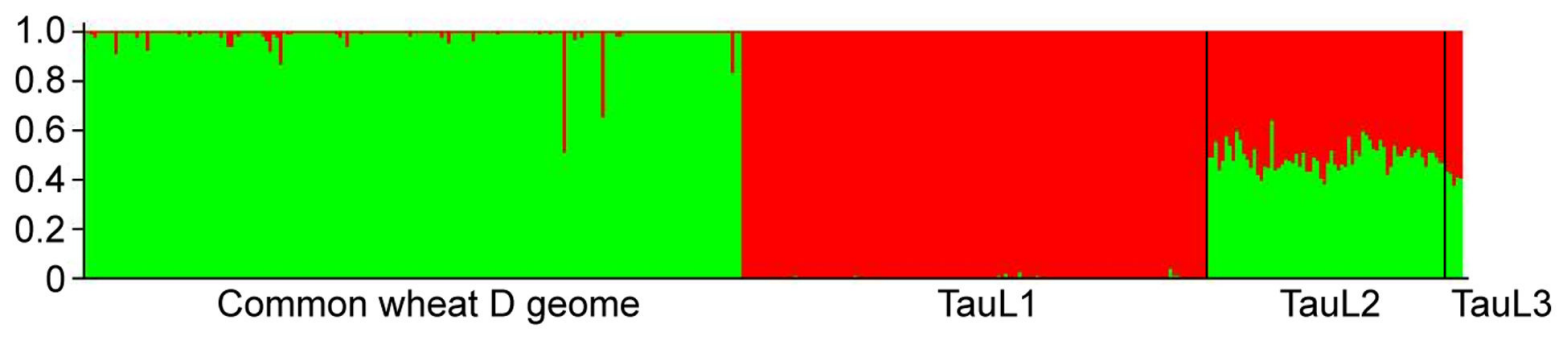
Propotional membership (Q) of each *Ae* *. tauschii* and *T. aestivum* sample accession at *K*=2.

To examine further the population structure of *Ae* *. tauschii* and its relationship to the D genome of common wheat, we estimated *K*, i.e., the number of putative genepools that best explained the pattern of variations at the DArT-marker loci, by the use of a Bayesian clustering approach implemented in the software program STRUCTURE 2.3 [27]. Calculation of ∆*K*, an *ad hoc* statistic based on the second order rate of change of likelihood of *K* [28], based on the STRUCTURE output indicated pronounced genepool differentiation at *K*=2 (Figure 2). At *K*=2, TauL1 and common wheat were grouped into distinct genepools, whereas TauL2 and TauL3 formed an intermediate genepool, a result consistent with the PCA result (Figure 3, Table S3). The distinction between TauL2 and TauL3 was not obvious, possibly because of the small accession sample size of TauL3. The PCA and Bayesian clustering, therefore, showed close genetic relationships of the TauL2 and TauL3 accessions to the D genome of common wheat. Of those, we chose the TauL2 accessions for further study, because these accessions had the necessary genotypic and phenotypic variability that was required for the genetic analyses of hybrid genome doubling (Figure 1, see below).

**Figure 4:**
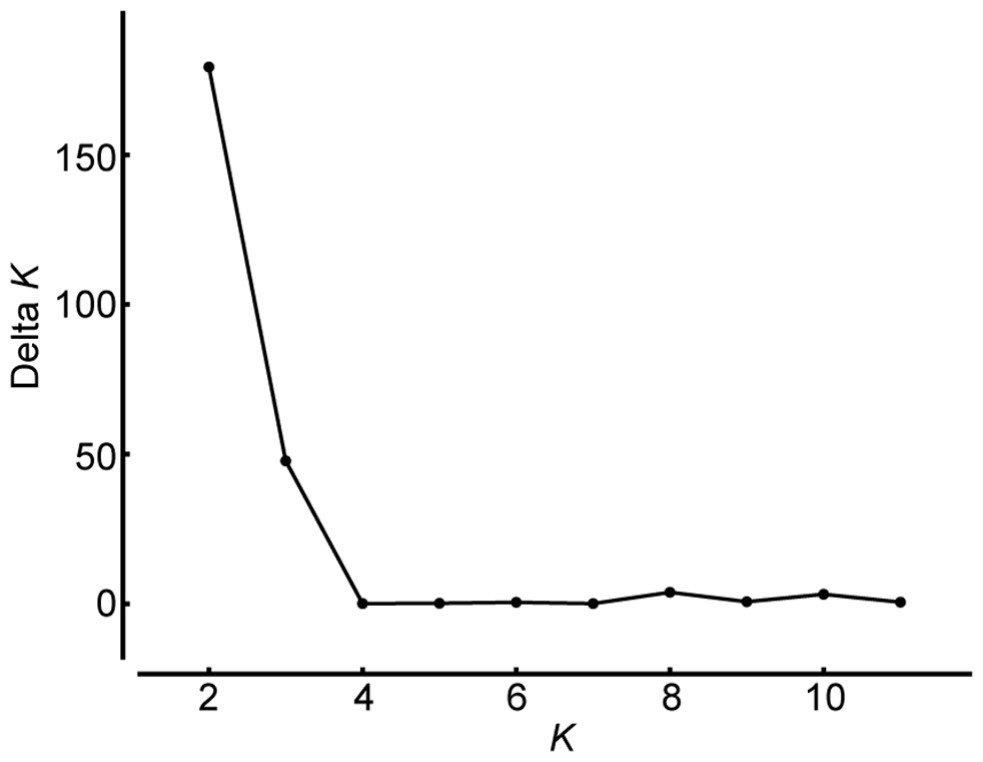
Plot of ∆*K* from the STRUCTURE analysis of the 187 *T. aestivum* accessions.

**Figure 5:**
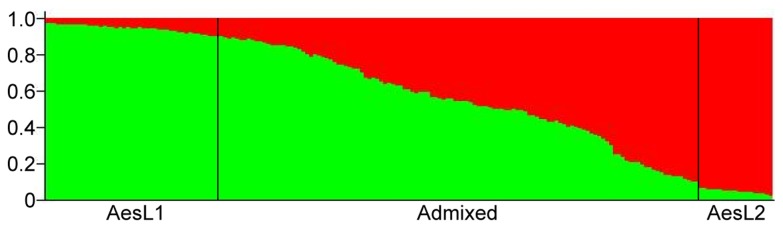
Proportional membership (*Q*) of each *T. aestivum* accession at *K*=2.

Notwithstanding the observed close genetic relationship of TauL2 with the D genome of common wheat, whether the TauL2 accessions represented descendants of the *Ae* *. tauschii* populations that were involved in the 8,000-years-ago allopolyploid speciation of common wheat was not clear. To address this issue, the divergence time between TauL2 and the D genome of common wheat was estimated by coalescent analysis. Prior to performing the analysis, we examined the structure of common wheat’s D genome population by Bayesian clustering since previous studies had suggested multiple origins for common wheat [29]. In a STRUCTURE analysis of 187 common wheat accessions (excluding W7984), common wheat’s D genome population was found to consist of two deeply structured genepools, consistent with the multiple origin hypothesis (Figure 4). In addition, possible less pronounced differentiation was suggested at *K*=3. At *K*=2, the 187 common wheat accessions were classified into three groups: AesL1, AesL2, and Admixed, using the threshold of 0.9000 for the *Q* statistics (i.e., the estimated membership coefficients for each individual in each genepool), (Figure 5). Interestingly, AesL1 mainly consisted of Oriental cultivars, whereas AesL2 was composed of Occidental cultivars (Table S4).

To reduce the complexity in coalescent modeling, the divergence time was inferred between AesL1 and TauL2 and between AesL2 and TauL2. The allopolyploid speciation of common wheat took place about 8,000 years ago after the domestication of *T* *. turgidum* 10,000 years ago, whereas the progenitors of the genus *Aegilops* radiated 2.5-4.5 million years ago (MYA) [30]. Accordingly, we compared a model that assumed a recent split through allopolyploid speciation 8,000-10,000 years ago (Model1) with another model that assumed a deep split of 10,000-2.5 million years ago (Model2) in each AesL1-TauL2 and AesL2-TauL2 analysis (Table 1). In Model1, one-way immigration from the diploids (TauL2) to hexaploids (AesL1 and AesL2) was allowed because each AesL1 and AesL2 might have been the product of multiple allopolyploid speciation events. In Model2, bidirectional immigration was assumed, because, for most of the simulated time span, each AesL1 and AesL2 represented a population of diploids that became hexaploids only after a recent speciation. The actual DArT marker mutation rates were not known, but these values might be higher than point mutation rates because the DNA variations detected by DArT markers are largely a result of nucleotide substitutions and indels at restriction sites [31]. For this reason, we used a mutation rate of 10^-8^ per site per generation as a conservative estimate [32].

**Table 1:** Prior distributions used in the approximate Bayesian computation approach to compare alternative demographic models for the AesL1-TauL2 and AesL2-TauL2 splits.

Parameter	Model1	Model2
Time of divergence	Uniform (8000-10,000 years ago)	Uniform (0.01-2.5 MYA)
Population size 1^a^	Uniform (0-10000 individuals)	Uniform (0-10000 individuals)
Population size 2^b^	Uniform (0-10000 individuals)	Uniform (0-10000 individuals)
Population size 3^c^	Uniform (0-10000 individuals)	Uniform (0-10000 individuals)
Immigration rate 1^d^	Uniform (0-0.0001)	Uniform (0-0.0001)
Immigration rate 2^e^	0	Uniform (0-0.0001)
Mutation rate^f^	1.0 × 10^-8^	1.0 × 10^-8^

a Effective population size of modern *Ae* *. tauschii* lineage TauL2

b Effective population size of modern common wheat D genome

c Effective population size of ancestral *Ae* *. tauschii* population

d Immigration rate from *Ae* *. tauschii* lineage TauL2 to the ancestor of common wheat D genome (measured as the fraction of immigrants per generation)

e Immigration rate from the ancestor of common wheat D genome to *Ae* *. tauschii* lineage TauL2 (measured as the fraction of immigrants per generation)

f Number of mutations per generation per locus.

**Figure 6:**
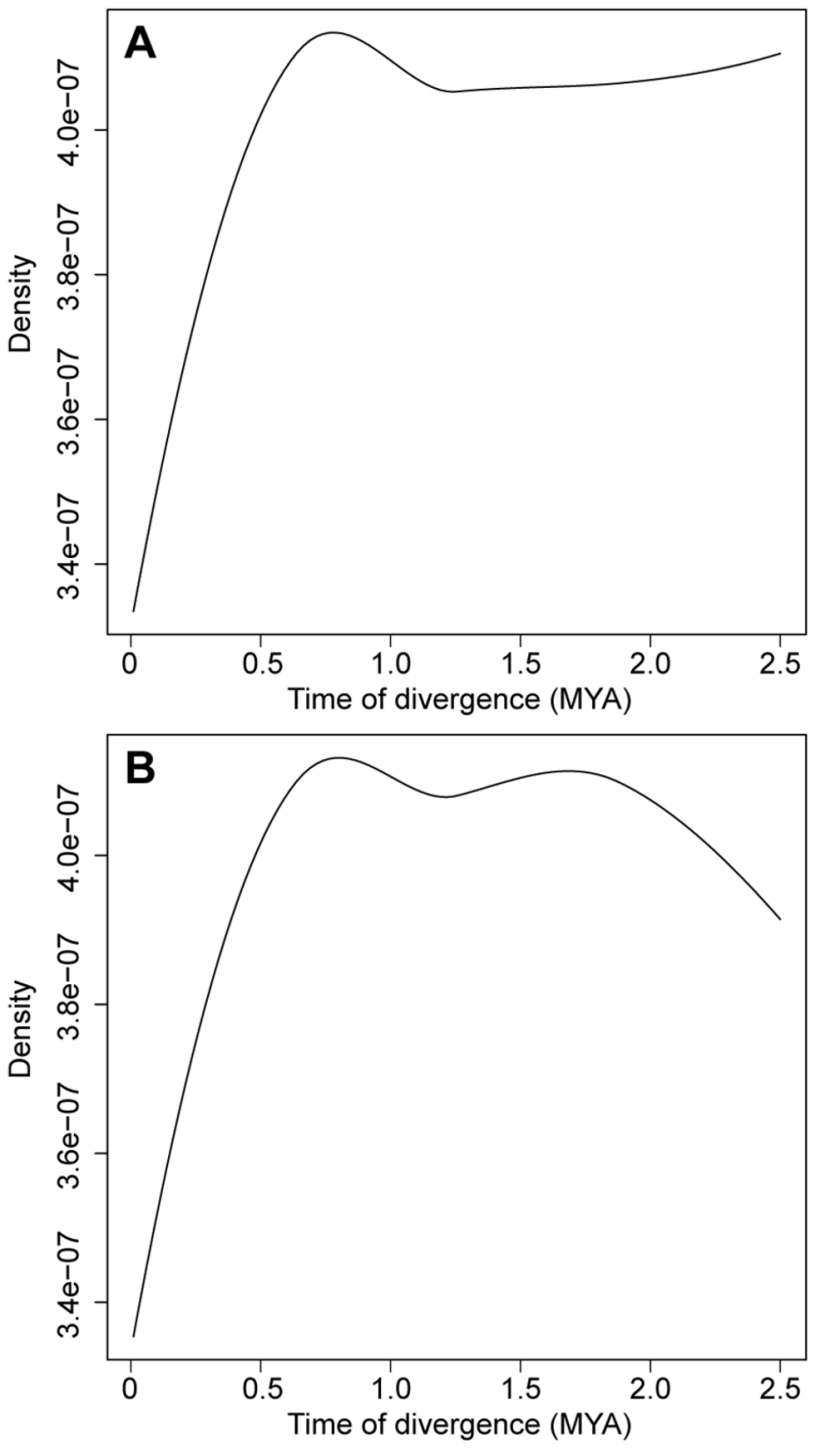
Posterior distribution of divergence time. A. AesL1-TauL2 split. B. AesL2-TauL2 split.

In the coalescent analyses, Model2 was found to fit the real data better than Model1 in both AesL1-TauL2 and AesL2-TauL2 splits because the proportions of the simulations accepted in the rejection step of ABC were larger for Model2 than for Model1: 0.29 (Model1) and 0.71 (Model2) in the AesL1-TauL2 analysis and 0.28 (Model1) and 0.72 (Model2) in the AesL2-TauL2 analysis. The posterior distributions of divergence time obtained by ABC under Model2 conditions indicated 0.5-1.0 MYA as the lower limit for both AesL1-TauL2 and AesL2-TauL2 splits (Figure 6). These results did not support the hypothesis that the D genome of common wheat was derived from an ancestor of the TauL2 accessions through allopolyploid speciation, whereas possible insufficient sampling of the *Ae* *. tauschii* and *T. aestivum* accessions and the use of a conservative estimate for DArT marker mutation rates might have biased the divergence-time estimates upward. Consequently, our population-level analyses identified TauL2 as one of the major existent *Ae* *. tauschii* lineages that are genetically closely related to, but not the closest sister of, the D genome of common wheat. We note, however, that this result requires further testing, because a recent large-scale single nucleotide polymorphism study suggested that a population within TauL2 was the main source of the common wheat D genome [26].

### The ability of *Ae* *. tauschii* to cause hybrid genome doubling

**Figure 7:**
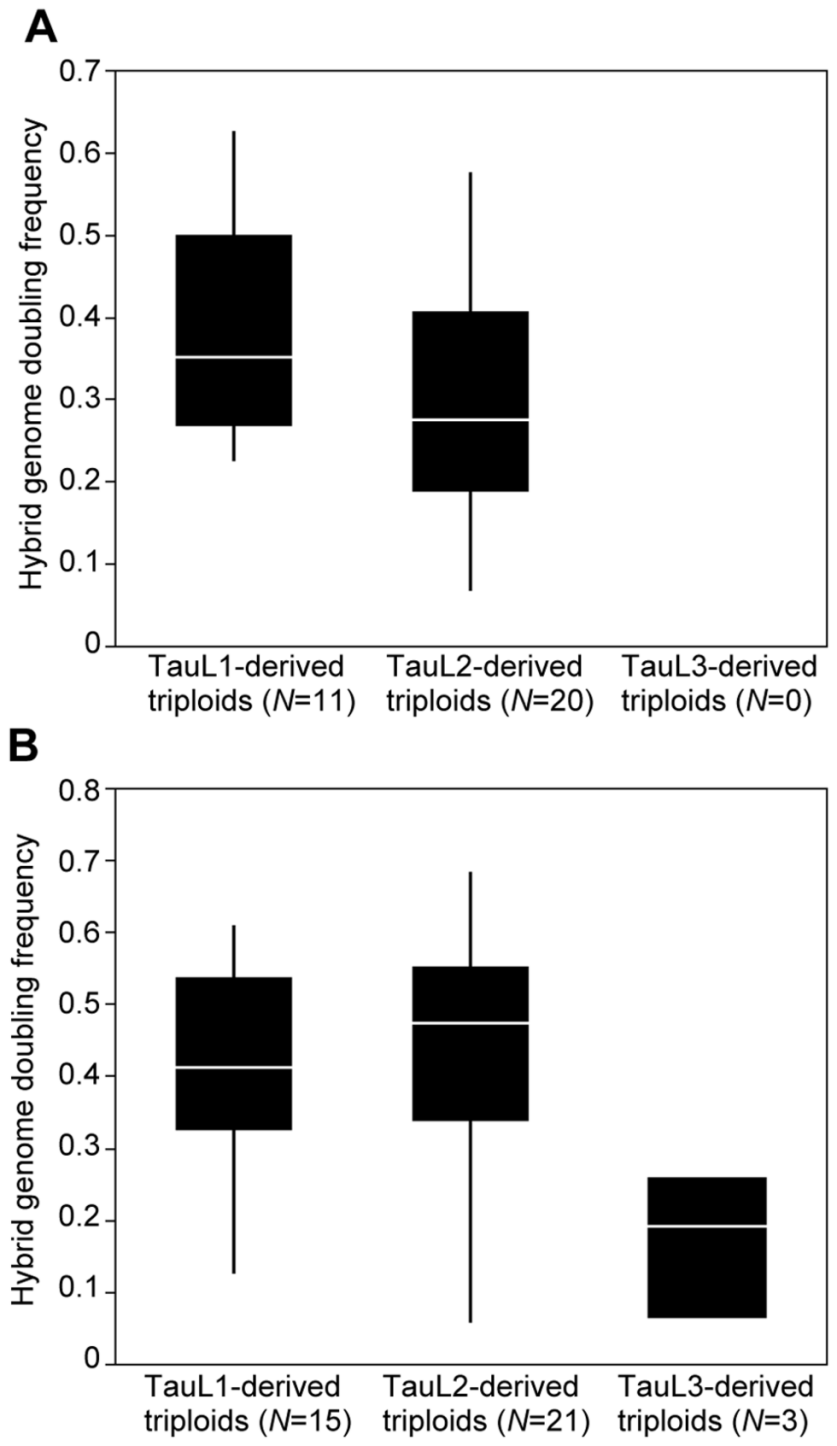
Box plots of genome doubling frequencies of LDN-*Ae*. ***tauschii* triploid F_1_ hybrids**. A. year 2004. B. year 2005.

We examined if the TauL2 accessions are more able to cause genome doubling in the triploid F_1_ hybrids with *T* *. turgidum* than the TauL1 and TauL3 accessions. In our previous experiment, 79 of the 206 *Ae* *. tauschii* accessions were artificially crossed with LDN, and genome doubling frequencies were obtained for 56 triploid F_1_ hybrids [11], (Y. Matsuoka, unpublished data). A reanalysis of that data showed that, within each lineage, the ability to cause genome doubling in the hybrids with LDN greatly varies between accessions: hybrid genome doubling frequencies ranging roughly from 0.1 to 0.6 in the TauL1-derived and TauL2-derived hybrids and from 0.06 to 0.26 in the TauL3-derived hybrids (Figure 7, Table S5). No obvious inter-lineage differentiation of that ability, however, was observed because in each year 2004 and 2005 the mean hybrid genome doubling frequencies did not significantly differ between the lineages (*p*=0.08-0.84, Steel-Dwass tests for all possible pairs in each year). These results did not indicate that the TauL2 accessions are more able to cause hybrid genome doubling than the TauL1 and TauL3 accessions, but support the inference that the *Ae* *. tauschii* ’s ability to cause hybrid genome doubling is a trait of deep evolutionary origin [11].

### Male and female fertilities of the HGD and LGD hybrids

The TauL2 accessions included multiple HGD-hybrid producers (KU-2088, KU-2092, KU-2103, KU-2104, KU-2106, KU-2111, and KU-2159) and LGD-hybrid producers (KU-2080 and KU-2097) (Table S5). To study the detailed genetic mechanism that underlies the genome doubling of *T* *. turgidum* *-Ae. tauschii* triploid F_1_ hybrids, the accessions KU-2103 (representing the HGD-hybrid producers) and KU-2080 (representing the LGD-hybrid producers) were chosen for further analyses. These two accessions were crossed with LDN and the HGD-hybrid (i.e., LDN-KU-2103 hybrid) and LGD hybrid (i.e., LDN-KU-2080 hybrid) produced without the use of gibberellic acid solution and embryo rescue techniques [8]. The hybrids grew to maturity without showing symptoms of necrotic dysgenesis under our growth conditions. Consistent with the previous results, the HGD hybrid showed high genome doubling frequencies (0.56 by self-crossing and 0.53 by spike-bagging), whereas the LGD hybrid had low genome doubling frequencies (0.08 by self-crossing and 0.05 by spike-bagging) (Table 2).

**Table 2:** Genome doubling frequencies of the HGD (i.e., LDN-KU-2013) and LGD (i.e., LDN-KU-2080) triploid F_1_ hybrids.

Hybrid	Selfing method	No. Plants	No. florets examined	No. seeds	Seed set rate
HGD	Crossing	6	466	263	0.56
	Bagging	6	370	197	0.53
LGD	Crossing	6	488	37	0.08
	Bagging	6	408	22	0.05

The relationship between hybrid genome doubling and the hybrids’ male and female fertilities was examined by a test cross experiment with the HGD and LGD hybrid plants using a hexaploid common wheat cultivar Chinese Spring (CS) as the reference line (Table 3). The male fertility of the HGD hybrid (0.76) was 3.3-hold higher than that of the LGD hybrid (0.23). Similarly, the female fertility of the HGD hybrid (0.64) was 2.1-fold higher than that of the LGD hybrid (0.30). The positive correlation of hybrid genome doubling frequencies with the degrees of hybrid male and female fertilities indicated that production of functional male and female gametes is essential for hybrid genome doubling to occur. In contrast, no obvious indications of post-gametogenesis activities that could negatively affect hybrid genome doubling (such as pollen-pistil incompatibility) were observed because in each hybrid the “by self-crossing” genome doubling frequency (0.56 for the HGD hybrid and 0.08 for the LGD hybrid) was not less than, but comparable to, the function of the male and female fertilities (0.49 for the HGD hybrid and 0.07 for the LGD hybrid). Furthermore, the hybrids’ emasculated florets set seeds only when pollinated, indicating that no apomictic activity was involved in this phenomenon. These findings showed that for the HGD and LGD hybrids, whether genome doubling occurs is mostly dependent on the successful production of functional male and female gametes.

**Table 3:** Test cross experiment to examine male and female fertilities of the HGD hybrid, LGD hybrid, and CS reference line.

Material, tested	Egg donor	Pollen donor	Emasculation	Treatment	No. Plants	No. florets examined	No. seeds	Seed set rate
HGD hybrid	CS	HGD hybrid	Yes	Crossing	9	240	182	0.76
	HGD hybrid	CS	Yes	Crossing	6	512	329	0.64
	HGD hybrid	None	Yes	Bagging	6	488	0	0
LGD hybrid	CS	LGD hybrid	Yes	Crossing	9	240	55	0.23
	LGD hybrid	CS	Yes	Crossing	6	512	152	0.30
	LGD hybrid	None	Yes	Bagging	6	476	0	0
CS	CS	CS	Yes	Crossing	2	100	96	0.96
	CS	CS	No	Bagging	9	340	324	0.95
	CS	None	Yes	Bagging	11	340	0	0

### Male sporogenesis of the HGD and LGD hybrids

Because no apomictic seed set was observed in the test cross experiment, the seed set of the HGD and LGD hybrids must have resulted from union of male and female unreduced gametes through fertilization. One logical hypothesis for the observed positive correlation of hybrid genome doubling frequencies with the degrees of male and female fertilities, therefore, is that functional unreduced gametes might be produced more abundantly in the HGD hybrid than in the LGD hybrid. In fact, both hybrids produced pollen grains that were intensively stained by aceto-carmine, but the variation in size and stain-intensity made it difficult to evaluate the frequencies of functional unreduced pollen grains based on morphology (data not shown). Accordingly, we tested the hypothesis by cytologically comparing sporogenesis in the hybrids. In this study, only male sporogenesis was analyzed because the abundance of pollen mother cells (PMCs) facilitated sampling and ensured that statistical tests could be conducted.

**Figure 8:**
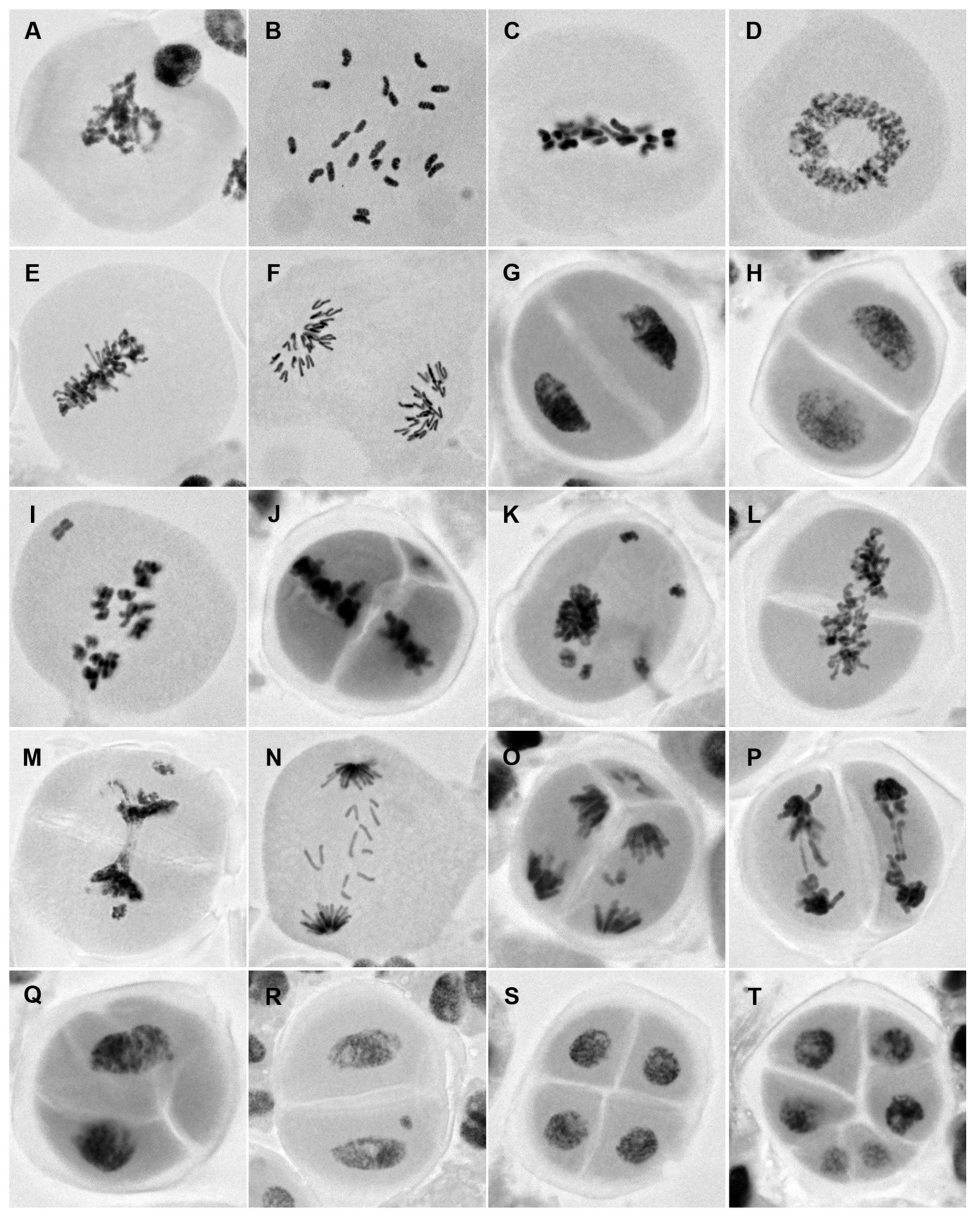
Pollen mother cell observations. Stages of normal nonreductional meiosis (A–H) and PMCs undergoing atypical cell divisions (I–T) are shown. The cells in A-I, L, N and Q were sampled from the HGD hybrid, the others are from the LGD hybrid. A. Prophase. B. Late prophase. Twenty-one univalents are visible. C. Metaphase. D. Restitutive nucleus (polar view). E. Restitutive nucleus (side view). F. Anaphase. G. Telophase. H. Dyad. I. Metaphase cell having a lagging chromosome. Sister chromatids are visible. J. Metaphase cell having a lagging chromosome. Irregular cytokinesis occurs in the direction vertical to the metaphase plate (i.e., vertical cytokinesis). K. Restitutive nucleus having decondensed lagging chromosomes. L. Restitutive nucleus undergoing vertical cytokinesis. M. Dumbbell-shaped restitutive nucleus. Irregular cytokinesis forcibly splits the restitutive nucleus. N. Anaphase cell having lagging chromosomes. O. Anaphase cell undergoing tri-polar division. The irregular cytokinesis seen in J seems to have resulted in the cells of this type. P. Anaphase cell having chromosome bridges. Chromosomes separate in each of the daughter cells that are produced through irregular cytokinesis. Q. Telophase cell undergoing irregular cytokinesis. R. Pollen dyad having a decondensed lagging chromosome. S. Tetrad. T. Hexad.

Our initial observation confirmed the occurrence of nonreductional meiosis that produces pollen dyads instead of tetrads in both HGD and LGD hybrids. Nonreductional meiosis differed from typical meiosis in that the PMCs underwent a single cell division (Figure 8). At late prophase, 21 univalent chromosomes that were randomly distributed in the PMCs became visible and subsequently aligned at the spindle equator at metaphase (Figure 8B-C). At this stage, chromosome pairing was not observed, indicative that homoeologous pairing is suppressed by the action of the *Ph* gene of LDN [33,34]. At late metaphase, the univalents split into sister chromatids, but failed to move to the spindle poles and formed a restitution nucleus (Figure 8D-E). Chromosomes of the PMCs underwent equational division at anaphase and started to decondense at telophase (Figure 8F-G). Symmetric dyad daughter cells were the final products of the process (Figure 8H). In parallel with the normal nonreductional process, atypical cell divisions that produce PMCs having irregular cytokinesis, lagging chromosomes, and bridges occurred in both HGD and LGD hybrids (Figure 8I–R). Irregular cytokinesis and lagging chromosomes were the most frequently observed abnomalities. Notably, these aberrations were limited to cells at metaphase or a later stage. The abnormal daughter cells such as tetrads and hexads probably were the products of such atypical cell divisions.

**Figure 9:**
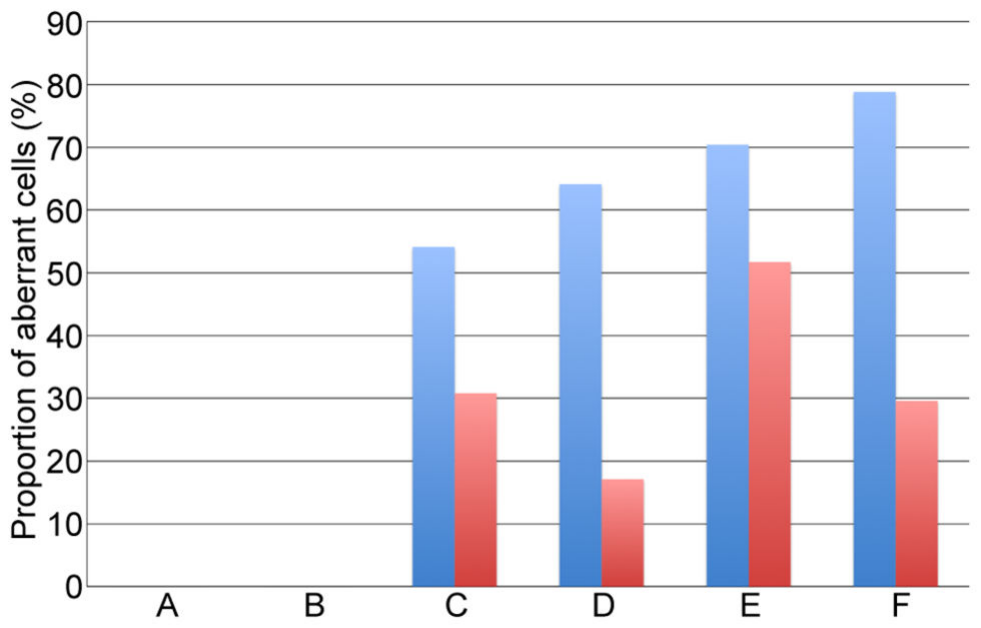
Stage-wise comparisons of aberrant cell frequencies. A. Prophase. B. Late prophase. C. Metaphase. D. Restitutive nucleus. E. Anaphase. F. Telophase/Dyads. The red and blue bars respectively denote the frequencies for HGD and LGD hybrids.

The hypothesis that functional unreduced gametes may be produced more abundantly in the HGD hybrid than in the LGD hybrid was tested by comparing the frequencies of aberrant PMCs between the hybrids. To promote unbiased representation of the cell division stages in the collected materials, this analysis was conducted using PMCs obtained from consecutive primary or secondary florets arrayed along the rachis of a spike. Typically, three to six consecutive florets covered prophase to the dyad stage. In total, six and five series of florets were used for PMC sampling in the HGD and LGD hybrids, respectively (26 florets for the HGD hybrid and 25 florets for the LGD hybrid). PMCs were randomly chosen from each preparation (20 cells per floret) and examined for the presence of irregular cytokinesis and lagging chromosomes. Consistent with our initial observations, no aberrations were observed in the PMCs at prophase and late prophase (Table S6). At metaphase and subsequent stages, the cells having irregular cytokinesis and/or lagging chromosomes were less frequent in the HGD hybrid (108 out of 348 cells, 31.0%) than in the LGD hybrid (203 out of 297 cells, 68.4%). A Pearson’s chi-squared test indicated that the difference in the cell counts was statistically significant (chi-squared value = 89.4, *p*=0.00). This result provided support for our hypothesis and showed that production of functional unreduced gametes through nonreductional meiosis is an early step key to successful hybrid genome doubling. In the stage-wise comparisons, aberrant cell frequencies were smaller for the HGD hybrid than for the LGD hybrid at metaphase and subsequent stages (Figure 9). The differences in the cell counts were statistically significant at metaphase (chi-squared value = 7.7, *p*=0.009), the restitutive nucleus stage (chi-squared value = 33.4, *p*=0.000), and telophase/dyads (chi-squared value = 58.3, *p*=0.000). At anaphase, the aberrant cells were less frequent in the HGD hybrid (30 out of 58 cells, 51.7%) than in the LGD hybrid (38 out of 54 cells, 70.4%), but the difference was not significant (chi-squared value = 4.1, *p*=0.055).

### Mechanisms for the meiotic nonreduction

To address the question of what cytological mechanisms are responsible for meiotic nonreduction in the unreduced gametes of the *T* *. turgidum* *-Ae. tauschii* F_1_ hybrids, we analyzed the modes of the single-cell-division pathway using molecular cytogenetic techniques that enable description of meiotic stages with respect to the cell-cycle dependent phosphorylation of histone H3. Phosphorylation of histone H3 at Ser10 is dependent on the cell cycle and an antibody against phosphorylated histone H3 provides a reliable indicator of cell division in normal plant meiosis: entire-chromosome immunosignals for the meiosis I divisions and pericentromeric immunosignals for the meiosis II divisions [35–37]. In fact, the entire-chromosome and pericentromeric patterns of immunosignals were observed in the male meiosis of common wheat (Figure S2).

**Figure 10:**
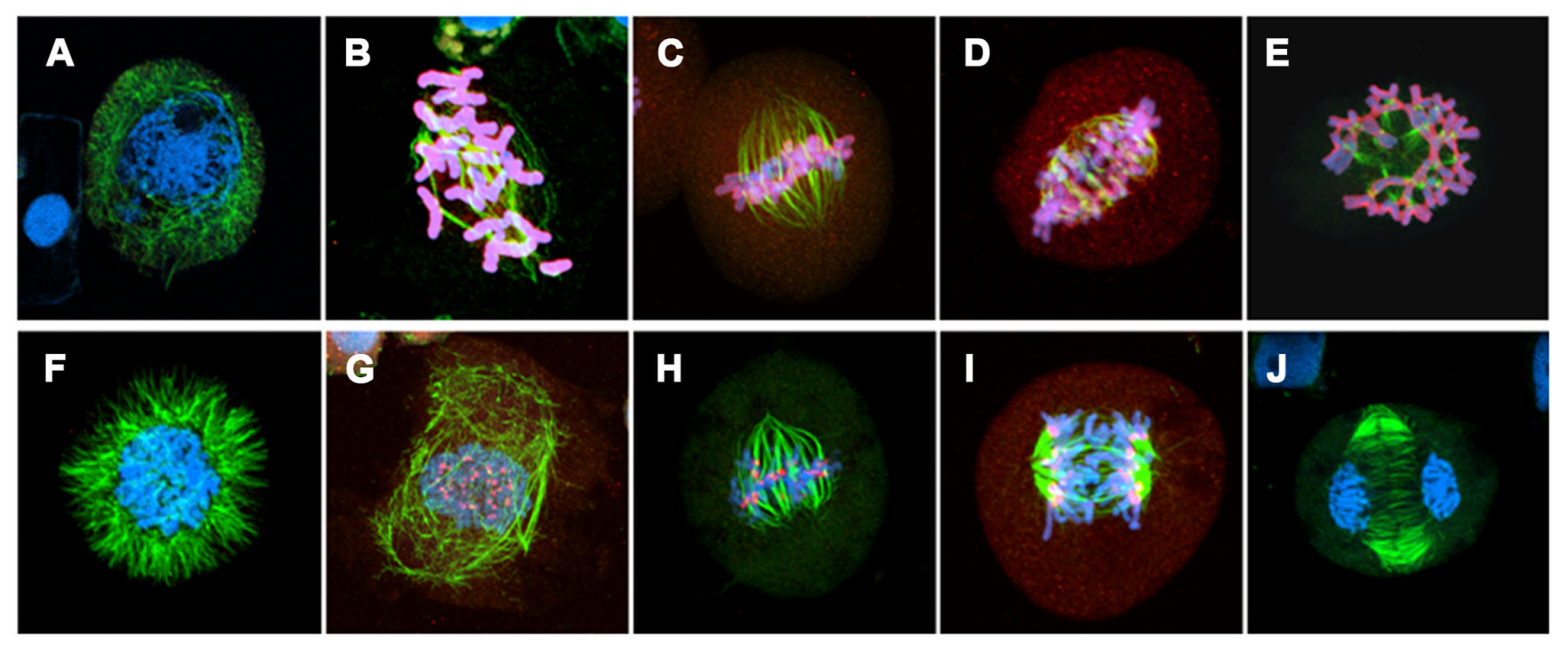
Immunostaining of male sporogenesis of the HGD hybrid. Merged images of the chromatin (blue), alpha-tubuling (green), and histone H3 phosphorylated at Ser 10 (phosphoH3S10) (red) signals. Essentially the same immunostaining pattern was observed for the LGD hybrid. The cells shown are from the HDG hybrids. A. Early prophase. B. Late prophase. C. Early metaphase. D. Late metaphase. E. Late metaphase (polar view). F. Restitutive nucleus. G. Late restitutive nucleus. H. Late restitutive nucleous. I. Anaphase. J. Telophase.

In the prophase and metaphase PMCs of the HGD and LGD hybrids, the phosphoH3S10 signals were observed on entire chromosomes (Figure 10A-B). The entire-chromosome signals were visible at late prophase, strongest at early metaphase, and weakened at late metaphase (Figure 10C–E). No phosphoH3S10 signals were observed at the early restitutive nucleus stage, indicating the chromosomes were decondensed (Figure 10F). At the late restitutive nucleus stage and anaphase, the phosphoH3S10 signals were observed at the pericentromeric region of the chromosomes (Figure 10G–I). At telophase during which cytokinesis occurred, the chromosomes were decondensed and the signals no longer visible (Figure 10J). We observed total reconstruction of the spindle structure at the restitutive nucleus stage. Early in this stage, the bipolar spindle structure formed at metaphase (Figure 10C) was fully dissociated (Figure 10F-G), whereas, later in this stage, the bipolar spindle structure was reconstructed (Figure 10H). These results showed that, in the single-cell-division pathway, cytokinesis did not occur when the chromosomes underwent the condensation pattern of normal meiosis I, whereas cytokinesis occurred when the chromosomes underwent the condensation pattern of normal meiosis II. In addition, the restitutive nucleus stage, in which the chromosomes underwent significant changes in condensation pattern, represented a critical stage in the single-cell-division pathway. Accordingly, the omission of the first division of normal meiosis was found to be one of the important cytological mechanisms that cause meiotic nonreduction in the *T* *. turgidum* *-Ae. tauschii* F_1_ hybrids.

### Mapping of quantitative trait loci that affect hybrid genome doubling

**Figure 11:**
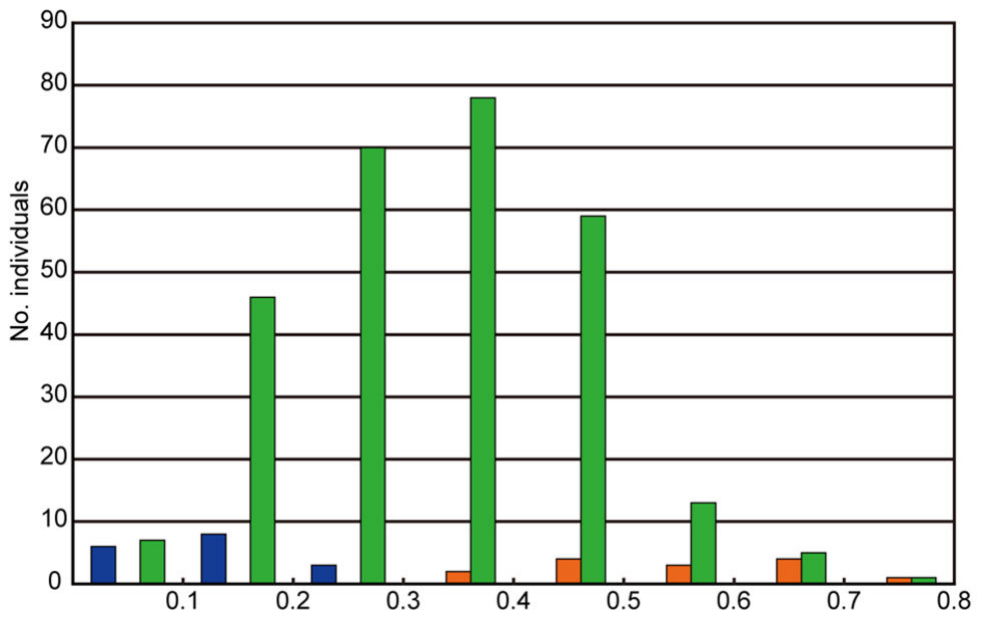
Hybrid genome doubling frequencies. The frequencies were measured as selfed seed set rates in the HGD hybrid (orange), the LGD hybrid (dark blue), and the segregants (green).

To evaluate what proportion of the variation in hybrid genome doubling occurrence is attributable to genetic factors, we estimated the broad-sense heritability of the trait. A population of triploid segregants (279 plants) was produced by crossing LDN with the F_1_ hybrid between *Ae* *. tauschii* accessions KU-2103 (the HGD-hybrid producer) and KU-2080 (the LGD-hybrid producer). The triploid segregants were then grown in a greenhouse with the HGD (14 plants) and LGD (17 plants) hybrids. To evaluate the hybrid genome doubling frequency for each plant, the first six spikes were bagged for selfing and 20 well-developed first and second florets per spike were examined for seed set. Hybrid genome doubling frequencies varied from 0.37 to 0.74 in the HGD hybrid (mean 0.56) and from 0.02 to 0.27 in the LGD hybrid (mean 0.14), whereas the segregants showed a widely ranging rate varying from 0.06 to 0.73 (mean 0.33) (Figure 11). Based on the observed variances, the estimated broad-sense heritability of hybrid genome doubling was calculated as 0.42 [{0.0161-0.5(0.0135+0.0051)}/0.0161] (0.32 when the rates were angle transformed) (Table 4). This result showed that, in these hybrids, the occurrence of genome doubling is under genetic control; however, environmental factors influenced the expression of this trait to a considerable degree.

**Table 4:** Means and variances of the hybrid genome doubling frequencies that were measured as selfed seed set rates in the HGD hybrid, LGD hybrid, and segregants.

	HGD hybrid	LGD hybrid	Segregants
Number of plants	14	17	279
Total number of florets examined	1680	2040	33650
Mean	0.561	0.135	0.325
Variance	0.0135	0.0051	0.0161
Mean, based on angle transformed rates	48.581	20.763	34.345
Variance, based on angle transformed rates	46.584	42.148	65.158

**Figure 12:**
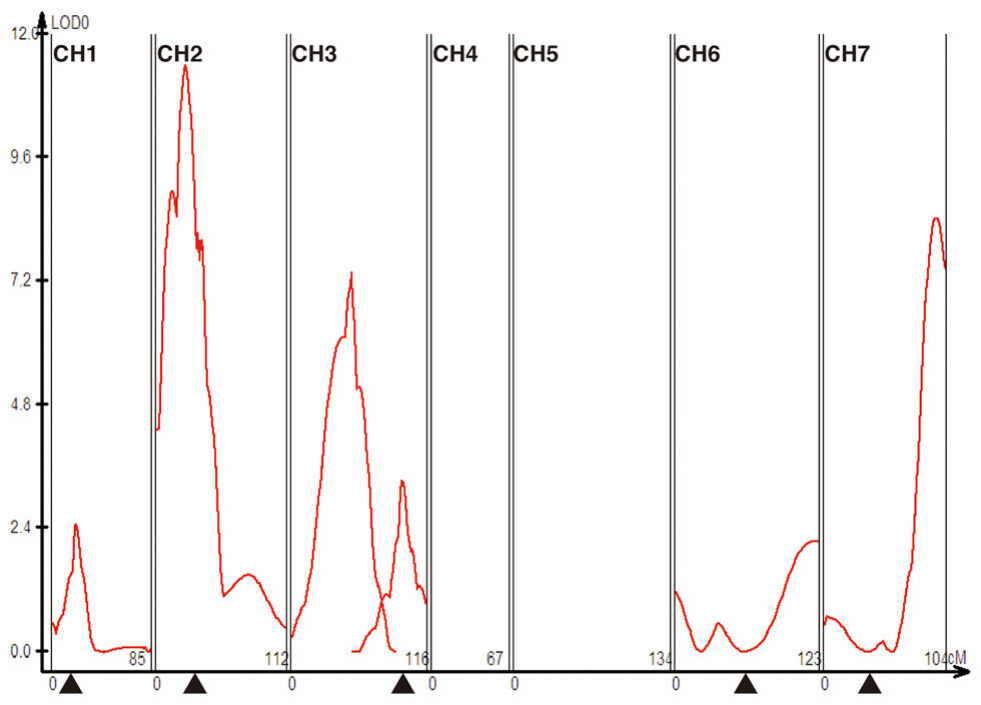
Multiple interval mapping of QTL associated with the occurrence of hybrid genome doubling. Black triangles indicate the approximate positions of centromeres estimated from the published wheat microsatellite consensus map [66].

To investigate the genetic basis of hybrid genome doubling, we addressed the question of how many genomic regions of *Ae* *. tauschii* contribute to the phenotype by performing QTL mapping. The 279 individuals of the triploid segregant population were genotyped by the use of 77 D-genome specific microsatellite markers. The linkage map based on those markers (total length 742.3 centimorgan) had 59.8% coverage of the wheat D genome (1242.1 centimorgan) [38]. In the QTL analyses, a model for doubled haploids was used because the data for the triploid segregants showed two genotype classes with no possibility of heterozygosity. Multiple interval mapping (MIM) [39] identified six genomic regions of *Ae* *. tauschii* as QTLs that were associated with the occurrence of hybrid genome doubling (Table 5; Figure 12). In a composite interval mapping analysis [40,41], the peaks for QTL1 (chromosome 1) and QTL5 (chromosome 6) did not meet the significance threshold criterion (LOD=2.5). Comparison of the six QTL and four QTL models through the MIM algorithm, however, confirmed that the former had an improved likelihood (-903.4) relative to the latter (-913.9). Among the six QTLs identified by MIM, QTL2 (chromosome 2), QTL3 (chromosome 3), and QTL6 (chromosome 7) had a relatively large effect and respectively explained 14.1%, 10.2%, and 11.3% of the phenotypic variance. All but one additive effect estimates for the QTLs were positive, indicating that the HGD-hybrid producer, *Ae* *. tauschii* accession KU-2103, provided the preponderance of alleles for high frequency hybrid genome doubling. In addition, one additive-by-additive epistatic interaction was detected between QTL2 and QTL6.

**Table 5:** QTLs and epistatic interaction that affect genome doubling frequency in the triploid F_1_ hybrids between *T* *. turgidum* and *Ae* *. tauschii* .

QTL/QTL combination^a^	Chromo- some^b^	Position^c^	Flanking SSR markers	LOD	Effect^d^	PVE (%)^e^
1	1	19.8	*Xwmc492* - *Xwmc216*	2.5	-1.3	2.7
2	2	24.3	*Xbarc168* - *Xwmc18*	8.0	2.7	14.1
3	3	50.7	*Xwmc533* - *Xcfd4*	7.4	2.3	10.2
4	3	94.4	*Xcfd223* - *Xcfd211*	3.4	1.6	4.9
5	6	118.0	*Xcfd76* - *Xwmc773*	2.2	1.3	2.9
6	7	94.4	*Xwmc671* - *Xwmc237*	6.3	2.6	11.3
2x6				1.5	-1.3	4.6

a Epistatic interaction between QTLs are denoted by “x”.

b *Ae* *. tauschii* chromosome that harbor the QTL.

c QTL position in centimorgan.

d Effect of the QTL/epistatic interaction on hybrid genome doubling frequency (unit: degrees).

e The proportion of the phenotypic variance explained by the QTL/epistatic interaction.

## Discussion

### Genetic basis for hybrid genome doubling in the *T* *. turgidum* - *Ae* *. tauschii* F_1_ hybrids

This study provided an answer to one of the important issues on the cytology and genetics of *T* *. turgidum* - *Ae* *. tauschii* hybrid genome doubling: the extent to which the functional unreduced gametes contribute to hybrid genome doubling. Previous studies agreed that functional unreduced gametes are produced in the *T* *. turgidum* - *Ae* *. tauschii* F_1_ hybrids, but the importance of this mechanism relative to other reproductive mechanisms (such as apomixis, pollen-pistil incompatibility, and seed abortion) in spontaneous genome doubling was not clear. In this study, the production of functional male and female gametes was found to be essential for genome doubling to occur in the HGD and LGD hybrids. In contrast, no obvious activity of other reproductive mechanisms was observed. By analyzing male sporogenesis in these hybrids, nonreductional meiosis was confirmed to be the pathway for the production of functional unreduced gametes in the male germline of the hybrids, whereas the occurrence of nonreductional meiosis in the female germline is safely inferred based on previous work [7,8]. Furthermore, significantly fewer aberrant PMCs were observed in the HGD hybrid than the LGD hybrid, providing evidence that production of functional unreduced gametes through nonreductional meiosis is an early step key to successful genome doubling. Taken together, all these findings underscore a predominant role of functional unreduced gametes in the *T* *. turgidum* - *Ae* *. tauschii* hybrid genome doubling.

Another important issue regarding the *T* *. turgidum* - *Ae* *. tauschii* hybrid genome doubling was the identity of the cytological mechanism causing meiotic nonreduction during gamete production. In diploid species, the omission of the first or second meiotic division may cause meiotic nonreduction. In such cases, the skipped division can be determined by sister chromatid analysis of the unreduced gametes: two non-sister chromatids and two sister chromatids residing in the unreduced gametes respectively indicate the first and second division omission [42]. Sister chromatid analysis, however, would not work in the *T* *. turgidum* *-Ae. tauschii* F_1_ hybrids because the lack of homologous chromosomes does not enable detection of the second division omission. Several previous studies observing PMCs agreed in that, unlike normal meiosis, the *T* *. turgidum* - *Ae* *. tauschii* F_1_ hybrids produce functional unreduced gametes through a pathway that involves a single cell division [7,8,15–22]. In many of those studies, the cytological mechanisms for meiotic nonreduction in the *T* *. turgidum* - *Ae* *. tauschii* triploid F_1_ hybrids were analyzed by the use of the concepts and terminology originally developed for nonreductional meiosis of diploid plant species that may produce unreduced gametes through meiotic defects such as the omission of the first or second meiotic division, abnormal spindle geometry, and abnormal cytokinesis [42]. The single-cell-division pathway was referred to as FDR based on the simple analogy with nonreductional meiosis of diploid plant species [19,20] or single-division meiosis solely based on the fact that the pathway undergoes a single cell division [8]. Omission of the first division was deduced from the defects observed in the PMCs [19,20], but whether the single-cell-division pathway skips one of the meiotic divisions had not rigorously been tested.

In this study, we addressed that question by charactering the mode of meiosis based on the immunosignal patterns of phosphoH3S10 on the chromosomes, i.e., entire-chromosome and pericentromeric signals respectively are reliable indicators of the first and second divisions in normal plant meiosis [36]. The results of the immunoanalysis showed that the chromosomes of hybrid PMCs underwent the condensation patterns that were typically observed in normal meiosis, despite the fact that cytokinesis occurred only at telophase. Furthermore, the restitutive nucleus stage, when the chromosomes underwent significant changes in condensation pattern, represented a critical stage in the single-cell-division pathway. In normal meiosis I, the sister kinetochores on the replicated sister chromatids are arranged side-by–side to ensure bipolar orientation of spindle when the homologous chromosomes pairs [43]. Throughout the meiosis I process, the pericentromeric Shugoshin proteins protect the cohesion of centromeric regions where the side-by-side sister kinetochores form [44,45]. In the case of nonreductional meiosis that is reported here, however, the side-by-side sister kinetochores formed bipolar spindles despite that no pairing was observed. For this reason, we speculate that restitution nuclei often found in the *T* *. turgidum* - *Ae* *. tauschii* F_1_ hybrids may be the cradle for dynamic chromatin-status transition (as indicated by the phosphoH3S10 localization pattern) that is likely associated with the change in the sister kinetochore configuration from the side-by-side (meiosis I) to the back-to-back (meiosis II) arrangements. Despite the recent progress made in cytological studies on the LDN-*Ae. tauschii* F_1_ hybrids [15], our view underscores the importance of accurate description of spindle attachment patterns in nonreductional meiosis, because whether the observed pattern is consistent with the spindle attachment/orientation of sister chromatids rather than that of homologous chromosomes is an essential question in understanding how the nonreductional process is comparable to normal meiosis. In terms of the timing of cytokinesis, the single-cell-division pathway could be viewed as FDR, a modified version of normal meiosis that skips the first division of normal meiosis. Consequently, this study provided evidence that FDR is one of the cytological mechanisms responsible for meiotic nonreduction in male sporogenesis of the hybrids. The formation of functional female unreduced gametes was inferred, but the occurrence of FDR in female sporogenesis remained to be examined [7,8].

Previous studies reported that the genotypes of the parental *T* *. turgidum* and *Ae* *. tauschii* accessions may influence the genome doubling frequencies of the triploid F_1_ hybrids [11,16–19,22]. This finding indicated that hybrid genome doubling is genetically controlled, but the details of the genetic mechanisms for hybrid genome doubling remained unknown. In this study, the results of MIM provided for the first time QTL evidence for *Ae* *. tauschii* having genes that influence genome doubling frequency when placed in the *T* *. turgidum* - *Ae* *. tauschii* triploid hybrid genome background. Because hybrid genome doubling frequencies were measured as selfed seed set rates, these QTLs may harbor genes that regulate such reproductive activities as gametogenesis, fertilization, and seed development in the hybrids. Nevertheless, we argue that many, if not all, of the six QTLs likely regulate nonreductional meiosis and its subsequent gamete production processes based on the predominant role of functional unreduced gametes in the genome doubling of HGD and LGD hybrids.

To date, genes involved in gametogenesis activities have not been reported from *Ae* *. tauschii* , but some genes with this function are known from the D genome of *T. aestivum*. Examples include *Ph2* located on the short arm of chromosome 3D [46–48], *Taf1* on the short arm of chromosome 2D [49], and an unnamed gene(s) on the short arm of chromosome 7D [50]. The *Ph2* gene is a suppressor of homeologous pairing. This study identified two QTLs (QTL3 and QTL4) on the short arm of chromosome 3D (Table 5; Figure 12). Of these, QTL3 might correspond to the *Ph2* gene, because, on the basis of the chromosomal bin information for the flanking makers, the possibility for this QTL to roughly overlap with the *Ph2* region could not be ruled out. The *Taf1* gene, which is involved in female sterility, might correspond to QTL2 found in this study. Consequently, four of six QTLs may represent a novel group of reproduction genes in wheat. In comparing male sporogenesis between the HGD and LGD hybrids, metaphase was found to be the earliest stage of nonreductional meiosis in which a significant difference in aberrant cell frequencies was detected. Accordingly, some of the QTLs might be involved in the regulation of such events as formation of bipolarly oriented univalents that occur at that stage [15].

LDN, the donor of the maternal component of the HGD and LGD hybrid genomes, has genes for genome doubling that function in hybrid genome backgrounds [51]. These results, together with the results of our study, indicate that, most likely, both maternal and paternal genetic factors are involved in the *T* *. turgidum* - *Ae* *. tauschii* F_1_ hybrid genome doubling. Interestingly, the synthetic haploid of LDN (AB genome) sets seeds of normal disomic plants (AABB genome) through union of male and female unreduced gametes produced via nonreductional meiosis [52]. This finding indicates that, even when the *Ae* *. tauschii* genome is absent, the LDN genome is capable of undergoing genome doubling. The genome doubling frequency of the synthetic LDN haploid (2.75 seeds per plant, measured as selfed seed set rate), however, is greatly increased by addition of the *Ae* *. tauschii* genome because the HGD and LGD hybrids set roughly 10-106 or more seeds per plant (based on the data in Table 2). Accordingly, the *Ae* *. tauschii* genes for hybrid genome doubling, especially the major alleles from the HGD-hybrid-producer, seem to have considerable positive impact on hybrid genome doubling when the LDN genome is merged. To what extent the additive effects of the *Ae* *. tauschii* genes are responsible for improvement of the hybrid genome doubling frequency and whether epistatic interactions between the LDN and *Ae* *. tauschii* genes are involved remain to be addressed.

### Implications for the evolution of common wheat

Hybrid genome doubling through the union of unreduced gametes represents an important mechanism for allopolyploid speciation [53]. In such cases as *T* *. turgidum* and *Ae* *. tauschii* , hybrid genome doubling is a generation-specific phenomenon that is expressed in F_1_ individuals, but not in the parental species nor the individuals of the F_2_ or later generations. The fitness of F_1_ individuals is greatly improved, whereas the adaptive significance of hybrid genome doubling for the parental species is not clear. How genes involved in hybrid genome doubling are maintained in the parental species is an intriguing question. To date, however, few studies have addressed this issue.

In our study, the ability of *Ae* *. tauschii* to cause genome doubling in the triploid F_1_ hybrids with LDN varied little between the TauL1, TauL2, and TauL3 lineages, but varied greatly within the lineages (Figure 7). The observed pattern of natural variation may provide some clues to answer the how-the-genes-are-maintained question based on an evolutionary model that assumes that, in *Ae* *. tauschii* , those six QTLs have some “normal” (presumably reproductive) function and that the observed natural variation in the ability to cause hybrid genome doubling is a by-product of non-deleterious mutations occurred at those QTLs. The ability of *Ae* *. tauschii* to cause hybrid genome doubling may have a deep evolutionary origin because the trait is commonly shared by accessions of the species. Accordingly, the six QTLs identified in this study likely are inherited from a common ancestor of the TauL1, TauL2, and TauL3 lineages and are maintained in each lineage because their “normal” function has some adaptive value. Mutations occur at the QTLs, but only the non-deleterious mutations that do not critically influence the “normal” function accumulate over time. Most such mutations likely remain phenotypically cryptic in *Ae* *. tauschii* , but each resultant QTL allele may positively or negatively affect genome doubling when placed in the hybrid genome background. The natural allelic variation for QTLs that arise through such a process may generate the within-lineage variability in *Ae* *. tauschii* ’s ability to cause hybrid genome doubling, whereas similar strength of natural selection on the “normal” function of the QTLs would result in fewer between-lineage differences. Non-deleterious mutations occurring at QTLs, therefore, may have had some important roles in shaping the pattern of that natural variation.

Our model to explain the pattern of natural variation regarding *Ae* *. tauschii* ’s ability to cause hybrid genome doubling sheds some light on the origin of common wheat. In this study, the population-level analyses showed that the TauL2 accessions, including the parents of the HGD and LGD hybrids, were genetically closely related to, but not the closest sister of, the D genome of common wheat. The association of the synthetic wheat line W7984 with *Ae* *. tauschii* indicated that possible nonspecific hybridization of the DArT markers to the A and B genomes of common wheat, if any, had no significant influence on the PCA result. Accordingly, a straightforward question may arise about the implications of this study’s finding of the mechanisms that underlie allopolyploid common wheat speciation. In contrast, the model for *Ae* *. tauschii* ’s natural variation in causing hybrid genome doubling suggests that, most likely, the QTLs identified in this study also were inherited and maintained in the two still-undiscovered groups of *Ae* *. tauschii* from which the current D genome of common wheat was derived. This hypothesis further suggests that, if the ancestral *Ae* *. tauschii* had hybridized with a LDN-like *T* *. turgidum* under natural conditions 8,000 years ago, the cytological and genetic mechanisms for hybrid genome doubling that gave rise to common wheat would have been essentially the same as those observed in this study. Accordingly, the findings of this study may provide an empirical basis for theories on the evolution of common wheat.

## Materials and Methods

### Plant materials, growth conditions, and DNA extraction

The plant materials used in this study were 206 accessions of *Aegilops* *tauschii* Coss. and 188 accessions of *Triticum aestivum* L. (Table S1). In addition, *Triticum* *turgidum* L. subsp. *durum* cv. ‘Langdon’ (LDN), and *T. aestivum* L. subsp. *aestivum* cv. ‘Chinese Spring’ (CS) were used as reference lines. Seeds of all plants, including the HGD and LGD hybrids and the triploid segregants used for QTL mapping, were germinated in Petri dishes at 23°C prior to transplanting to individual pots in a greenhouse in late November. The greenhouse was slightly heated (the temperature was kept above 10°C) during the first 3-4 weeks to enhance early development but was unheated thereafter. The monthly means of minimum daily greenhouse temperatures were 2.6°C (January), 2.6°C (February), and 4.1°C (March). Total DNA was extracted from the young leaves of individual plants by the CTAB method [54,55].

### DArT marker genotyping

The DArT marker genotyping was done at Diversity Arrays Technology Pty. Ltd., Yarralumla, Australia, using the array that was developed for *Ae* *. tauschii* genotyping [56,57]. Of the several thousand markers that showed polymorphisms between the accessions, 169 were selected based on the data quality, redundancy, and reproducibility. To reduce the influence of possible nonspecific hybridization when the array was used for *T. aestivum*, the markers that detect A and B genome loci were excluded based on the information provided by Diversity Arrays Technology Pty. Ltd. The 169 DArT-marker loci were spread across the seven chromosomes of the D genome of common wheat. The genotype data for 19 *Ae* *. tauschii* accessions (AT 47, AT 76, CGN 10734, CGN 10768, IG126991, IG127015, IG 47202, IG 47203, IG 49095, KU-2022, KU-2035, KU-2063, KU-2069, KU-2097, KU-2109, KU-2136, KU-2159, KU-2809, and KU-2814) were obtained from Sohail et al. [57]. The genotypes at each locus were scored as either presence (coded as “1”) or absence (coded as “0”) of hybridization to the corresponding array element. The missing data percentages ranged from 0% to 19.5% (mean 2.5%) between accessions and 0% to 17.0% (mean 2.5%) between markers.

### Principal component analysis

PCA was done with the among-accession covariance matrix using JMP 9 software (SAS Institute, Inc). Missing genotype scores were coded as “0.5” in this analysis.

### Bayesian clustering

Bayesian clustering was done using the STRUCTURE 2.3 software [27]. Because *Ae* *. tauschii* and *T. aestivum* are largely homozygous, we used a haploid setting. The algorithm was run with a burn-in length of 50,000 and then 20,000 Markov Chain Monte Carlo simulations for estimating parameters. We performed 10 independent runs for each *K* between 1 and 12 using the admixture model and correlated allele frequencies. The STRUCTURE HARVESTER software [58] was used to calculate the Evanno’s ∆*K* values and the CLUMPP software [59] to combine the outputs from the STRUCTURE software with the FullSearch algorithm.

### Coalescent analysis

Coalescent analysis was performed using an approximate Bayesian computation (ABC) approach implemented in the software program popABC [60]. The framework of ABC estimates population and genetic parameters and compares alternative demographic models based on the estimated Bayesian posterior probability. The statistics used to summarize the DArT genotype data in the approximate Bayesian computation (ABC) were the mean of pairwise differences, the number of segregating sites, the number of haplotypes, Shannon’s index [61], the mean of the Mutation Frequency Spectrum [62], the Nm-related statistic using the number of segregating sites, the number of private segregating sites, and the frequency of private segregating sites [63]. Some of these summary statistics were highly correlated so that the ABC was done without using a regression step. Instead, we simulated the DArT genotype data a large number of times (5,000,000 times) and accepted 50,000 simulations at the rejection step in each model, because, when the number of simulated points is large, the difference between the results obtained by the “with regression” and “without regression” methods is negligible [64]. In the model comparison, the model that fit better to the real data was determined using a rejection step with a tolerance value of 0.01.

### Hybrid production

The *T* *. turgidum* - *Ae* *. tauschii* F_1_ hybrids were produced as previously described [8]. Durum wheat ( *T* *. turgidum* subsp. *durum* cv. ‘Langdon’) was chosen as the egg parent in the crosses, because of its candidacy for the female progenitor of *T. aestivum* [8].

### Crossing, selfing, and evaluation of hybrid genome doubling frequency

The method of crossing was hand pollination using fresh pollen collected at anthesis and fully emasculated spikes. Selfing was done by individually bagging the spikes prior to flowering, whereas, for reassessing the genome doubling frequencies of the HGD and LGD hybrids (Table 2), selfing was done by spike-bagging and self-crossing (i.e., pollinating the hybrid’s florets with its own pollen). Well-developed first and second florets were used for crossing and evaluating hybrid genome doubling frequencies. Selfed seed set rates were calculated as the number of seed sets/number of florets examined.

### Evaluation of male and female fertilities

Male fertility of the hybrids was evaluated as the seed set rate of CS when pollinated with hybrid pollen. Female fertility of the hybrid was evaluated as the seed set rate of the hybrids when pollinated with CS pollen.

### Observations of carmine-stained PMCs

Meiotic cell divisions were analyzed by the conventional aceto-carmine squash method. Immature anthers were fixed in a mixture of absolute ethanol and acetic acid (3:1) and stored at 4°C until use. For the cytological observations, fixed anthers were stained with aceto-carmine and squashed in a drop of 45% acetic acid. The cell images were documented using an Olympus BX-51 microscope and an Olympus DP21 digital camera. To compare the aberrant PMCs frequencies between the triploid F_1_ hybrids, two preparations per floret were made using two fixed anthers (one preparation from one anther). The accession of origin was anonymous to the observer. In each preparation, the cells were observed at a low magnification and then numbered and randomized to sample 10 cells per anther from 30–60 cells for in-depth observations. Pearson’s chi-squared tests were done using the freely available software package R [65]. The simulated *p*-values were based on 10,000 replicates.

### Immunostaining

Wheat anthers containing meiocytes were collected from young ears 4-5cm in the length. Meiotic stages were determined by aceto-carmin squashes of one of the three anthers in one floret, and the remaining two were fixed over night in freshly prepared 4% (w/v) paraformaldehyde dissolved in PMEG buffer (25 mM PIPES, 5 mM MgSO_4_, 5 mM EGTA, pH 6.9), then washed three times in PMEG buffer. Post-fixed anthers were stored in PMEG buffer at 4^o^C until analyzed. The anther was digested on a MAS-coated slide glass (Matsunami) at 37 ^o^C for 30 min in a mixture of 2.5% (w/v) Cellulase Onozuka R-10 (Yakult) and 2.5% (w/v) Pectolyase Y-23 (ICN) dissolved in PMEG buffer and squashed under a coverslip. After freezing in a deep-freezer (-70 ^o^C), the coverslip was removed with a razor blade, and the slides were transferred immediately to PMEG buffer at room temperature. Slides were incubated over night with the primary antibodies in a humid chamber at 4 ^o^C. The primary antibodies used were anti-alpha-tubulin antibodies (NeoMarkers) diluted 1:100 and anti-histone H3 phosphorylated at Ser 10 (phosphoH3S10) antibodies (Upstate) diluted 1:200 in PMEG buffer containing 1% (w/v) BSA and 0.1% (v/v) Tween 20. After overnight incubation, slides were washed for 15 min in PMEG buffer and incubated with secondary antibodies for one hour at 37 ^o^C. The secondary antibodies were Alexa Fluor 488-conjugated anti-mouse IgG and Alexa Fluor 546-conjugated anti-rabbit IgG goat F(ab’)_2_ fragment (Invitrogen), both diluted 1:200 in PMEG buffer containing 1% (w/v) BSA and 0.1% (v/v) Tween 20. After a final wash in PMEG buffer for 15 min, the slides were mounted in the antifade mixture (0.5% (w/v) N-propyl gallate and 80% (v/v) glycerol in 20 mM Tris-HCl pH 8.0) containing 4’,6-diamidino-2-phenylindole dihydrochloride (DAPI, 1.5 µg/ml). Fluorescent signals were detected with a Fluoview FV500 confocal laser microscopy (Olympus) for both immunostaining and FISH analyses. The digital images were captured by FLUOVIEW ver. 4.2 (Olympus) and processed with Photoshop ver. 6.0 (Adobe).

### Microsatellite genotyping and linkage map construction

A total of 312 D genome microsatellite marker primer sets (100 WMC [66], 88 CFD [67], 70 GWM [68,69], 45 GDM [70], eight BARC [71], and one KSUM [72]) were used to screen the parental *Ae* *. tauschii* accessions and LDN for polymorphisms and stable amplification. The microsatellite PCR reaction mixture consisted of a 50 ng total DNA template, 2 nmol each dNTP, 10 pmol each primer, 2 µl of 10X buffer, 0.1 U *Ex Taq* polymerase (TaKaRa), and distilled water to 20µl. PCR amplification was done in a Model 9700 (Applied Biosystems) thermal cycler and the runs were: a 3-min initial denaturing step at 94°C followed by a total of 40 cycles at 94°C for 0.5 min, 55°C or 60°C (depending on the primer set) for 0.5 min, and 72°C for 0.5 min, plus a 7-min final extension at 72°C. PCR products were separated by capillary gel electrophoresis using a QIAxcel device (Qiagen). For linkage map construction, the computer program MAPMAKER/EXP version 3.0b [73,74] was used with the Kosambi function [75]. Of the 131 markers that passed the initial screening, a set of 75 markers provided reasonable linkage groups under the criteria of minimum LOD threshold 3.0. In addition, two markers *Xwmc167* (chromosome 2) and *Xwmc773* (chromosome 6) were included. In each linkage group, the most plausible marker order was determined by the use of the “compare”, “order”, and “try” commands (Table S7).

### QTL mapping

Because the MIM and CIM algorithms assume a normal distribution for the residual errors, the selfed seed set rate data were angle-transformed prior to analysis. QTL mapping was done using the program Windows QTL Cartographer version 2.5 [76]. The initial MIM model was built by the use of the “MIM forward search” method and subsequently refined through optimizing QTL positions, searching for new QTLs/QTL interactions, and testing for existing QTLs/QTL interactions. As recommended by the authors, significance of the detected QTL was determined based on the BIO-M0 criterion. MIM walk speed was set to one centimorgan. CIM (model 6) was done with a 10-cM window and five background control markers. CIM walking speed was set to two centimorgan and the forward and backward method were chosen for regression. The significance threshold was determined by 1000 permutation.

## Supporting Information

Figure S1 **Geographic distribution of the *Ae* *. tauschii* accessions.** Purple, green, and red circles respectively denote the TauL1, TauL2, and TauL3 accessions. One accession from Armenia (CGN 10734) and six accessions from central China (AT 47, AT 55, AT 60, AT 76, AT 80, and PI508264) are not shown.
(TIF)

Figure S2 **Stage specific distribution pattern of phosphorylated histone H3 at Serine 10 (phosphoH3S10) in male meiosis of common wheat cultivar Chinese Spring.** Chromatin and phosphoH3S10, as denoted phH3S10 in this figure, are shown in red and green, respectively. Two images of cells at each meiotic stage are shown as a pair; (left) merged image of chromatin and phosphoH3S10, and (right) signals of phosphoH3S10. (A–H) Cells are in first meiotic division, (I–P) in second meiotic division. Note the contrasting localization of phosphoH3S10 in first and second meiotic divisions; whole arm localization in meta- and anaphases of first meiosis (C, D, E, F), and centromeric localization in meta- and anaphases of second division (K, L, M, N).
(TIF)

Table S1 **The *Ae* *. tauschii* and *T. aestivum* accessions used.** Source codes are IPK for Institut für Pflanzengenetik und Kulturpflanzenforschung, CGN for Centre for Genetic Resources, The Netherlands, ICARDA for International Center for Agricultural Research in the Dry Areas, KYOTO for Plant Germ-plasm Institute of Kyoto University, NBRP for National BioResources Project, OKAYAMA for Dr. Kenji Kato, Okayama University, and USDA for US Department of Agriculture. A hyphen indicates that the information is not available.
(DOCX)

Table S2 **Principal component scores used to provide Figure 1.** The lineage classification is provided for the *Ae* *. tauschii* accessions. A hyphen indicates that the information is not available.
(DOCX)

Table S3 **The STRUCTURE membership coefficients of the *Ae* *. tauschii* and *T. aestivum* accessions (*K*=2).** The *Ae* *. tauschii* lineages are based on the PCA (Figure 1). A hyphen indicates that the information is not available.
(DOCX)

Table S4 **The STRUCTURE membership coefficients of the *T. aestivum* accessions excluding W7984 (*K*=2).** The lineage classification of the accessions is provided. A hyphen indicates that the information is not available.
(DOCX)

Table S5 **Genome doubling frequencies of the LDN-*Ae.tauschii* triploid F_1_ hybrids.** A hyphen indicates that the information is not available.
(DOCX)

Table S6 **Comparison of normal and aberrant PMC counts between the HGD and LGD hybrids.**
(DOCX)

Table S7 **Linkages and positions of microsatellite markers used for the QTL analysis of hybrid genome doubling.**
(DOCX)
