# Supplementary figures and images for: Genetic Basis for Spontaneous Hybrid Genome Doubling during Allopolyploid Speciation of Common Wheat Shown by Natural Variation Analyses of the Paternal Species
Source: PLoS One. 2013 Aug 8;8(8):e68310. doi: 10.1371/journal.pone.0068310 (PMC3738567; doi:10.1371/journal.pone.0068310)

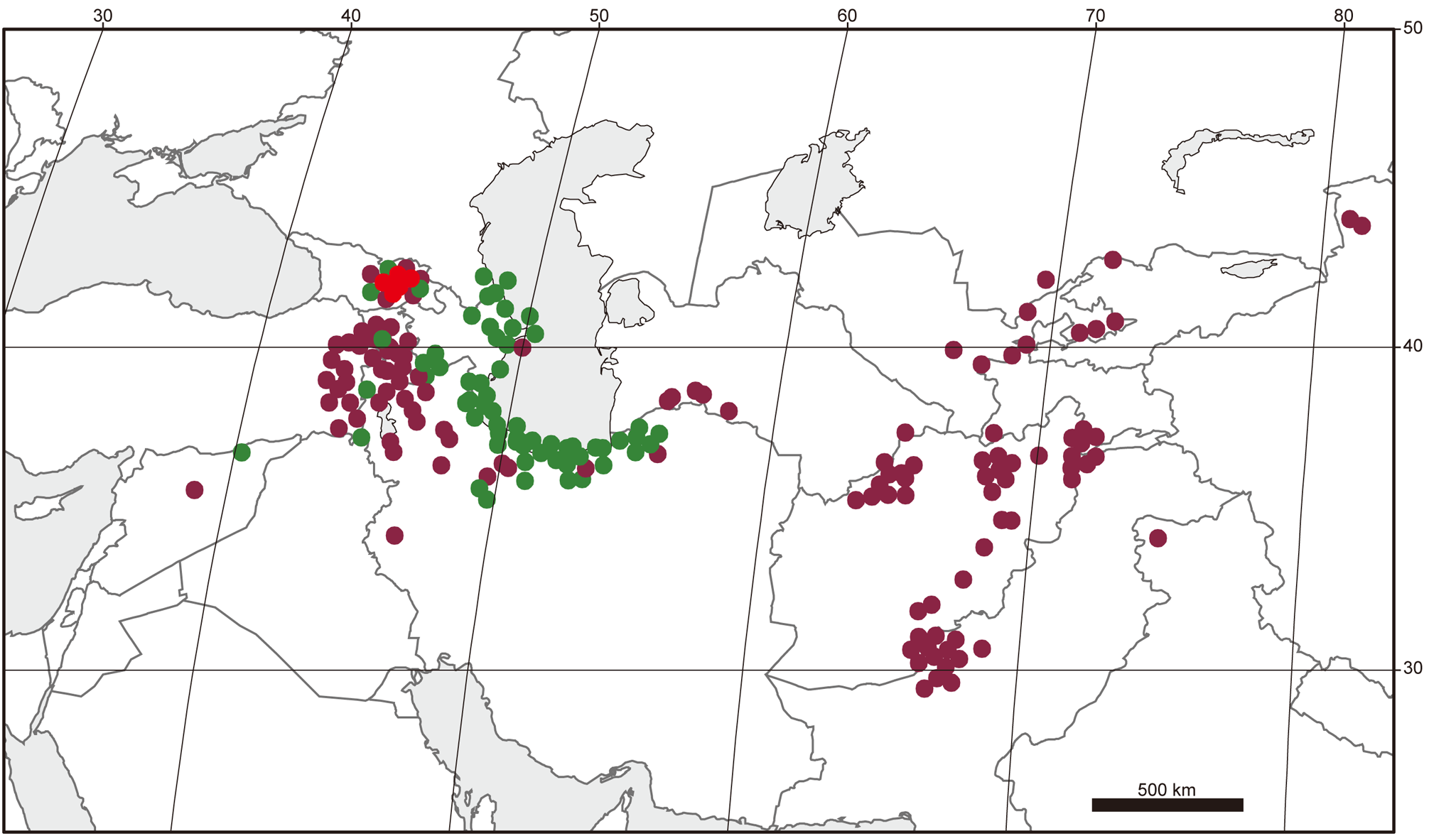

Supplement: Figure S1 — Geographic distribution of the Ae . tauschii accessions. Purple, green, and red circles respectively denote the TauL1, TauL2, and TauL3 accessions. One accession from Armenia (CGN 10734) and six accessions from central China (AT 47, AT 55, AT 60, AT 76, AT 80, and PI508264) are not shown. (TIF) [file pone.0068310.s001.tif]

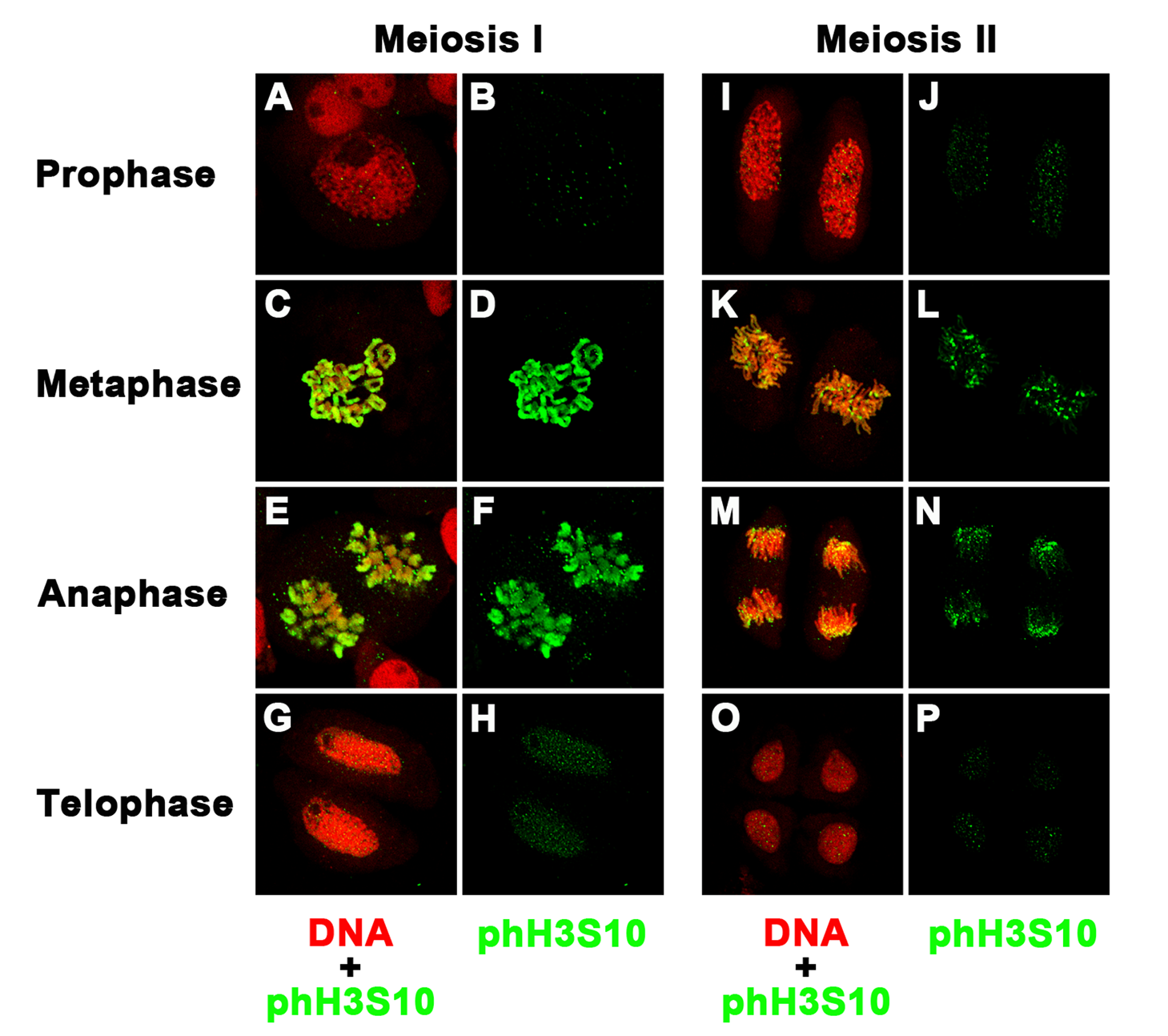

Supplement: Figure S2 — Stage specific distribution pattern of phosphorylated histone H3 at Serine 10 (phosphoH3S10) in male meiosis of common wheat cultivar Chinese Spring. Chromatin and phosphoH3S10, as denoted phH3S10 in this figure, are shown in red and green, respectively. Two images of cells at each meiotic stage are shown as a pair; (left) merged image of chromatin and phosphoH3S10, and (right) signals of phosphoH3S10. (A–H) Cells are in first meiotic division, (I–P) in second meiotic division. Note the contrasting localization of phosphoH3S10 in first and second meiotic divisions; whole arm localization in meta- and anaphases of first meiosis (C, D, E, F), and centromeric localization in meta- and anaphases of second division (K, L, M, N). (TIF) [file pone.0068310.s002.tif]
