# Supplementary material for: Genetic Basis for Spontaneous Hybrid Genome Doubling during Allopolyploid Speciation of Common Wheat Shown by Natural Variation Analyses of the Paternal Species
Source: PLoS One. 2013 Aug 8;8(8):e68310. doi: 10.1371/journal.pone.0068310 (PMC3738567; doi:10.1371/journal.pone.0068310)
Supplement: Table S1 — The Ae . tauschii and T. aestivum accessions used. Source codes are IPK for Institut für Pflanzengenetik und Kulturpflanzenforschung, CGN for Centre for Genetic Resources, The Netherlands, ICARDA for International Center for Agricultural Research in the Dry Areas, KYOTO for Plant Germ-plasm Institute of Kyoto University, NBRP for National BioResources Project, OKAYAMA for Dr. Kenji Kato, Okayama University, and USDA for US Department of Agriculture. A hyphen indicates that the information is not available. (DOCX) [file pone.0068310.s003.docx]

Table S1. The *Ae. tauschii* and *T. aestivum* accessions used.

| No. | Species | Accession | Origin | Source |
| --- | --- | --- | --- | --- |
| 1 | *Aegilops tauschii* Coss. | AE 1037 | Georgia | IPK |
| 2 | *Aegilops tauschii* Coss. | AE 1038 | Tajikistan | IPK |
| 3 | *Aegilops tauschii* Coss. | AE 1090 | Kazakhstan | IPK |
| 4 | *Aegilops tauschii* Coss. | AE 454 | Georgia | IPK |
| 5 | *Aegilops tauschii* Coss. | AE 457 | Georgia | IPK |
| 6 | *Aegilops tauschii* Coss. | AE 929 | Georgia | IPK |
| 7 | *Aegilops tauschii* Coss. | AE 933 | Georgia | IPK |
| 8 | *Aegilops tauschii* Coss. | AT 47 | China | OKAYAMA |
| 9 | *Aegilops tauschii* Coss. | AT 55 | China | OKAYAMA |
| 10 | *Aegilops tauschii* Coss. | AT 60 | China | OKAYAMA |
| 11 | *Aegilops tauschii* Coss. | AT 76 | China | OKAYAMA |
| 12 | *Aegilops tauschii* Coss. | AT 80 | China | OKAYAMA |
| 13 | *Aegilops tauschii* Coss. | CGN 10734 | Armenia | CGN |
| 14 | *Aegilops tauschii* Coss. | CGN 10767 | Pakistan | CGN |
| 15 | *Aegilops tauschii* Coss. | CGN 10768 | Pakistan | CGN |
| 16 | *Aegilops tauschii* Coss. | CGN 10769 | Pakistan | CGN |
| 17 | *Aegilops tauschii* Coss. | CGN 10770 | Pakistan | CGN |
| 18 | *Aegilops tauschii* Coss. | CGN 10771 | Pakistan | CGN |
| 19 | *Aegilops tauschii* Coss. | IG 108561 | Pakistan | ICARDA |
| 20 | *Aegilops tauschii* Coss. | IG 120735 | Turkmenistan | ICARDA |
| 21 | *Aegilops tauschii* Coss. | IG 120736 | Uzbekistan | ICARDA |
| 22 | *Aegilops tauschii* Coss. | IG 120863 | Dagestan | ICARDA |
| 23 | *Aegilops tauschii* Coss. | IG 120866 | Dagestan | ICARDA |
| 24 | *Aegilops tauschii* Coss. | IG 123910 | Uzbekistan | ICARDA |
| 25 | *Aegilops tauschii* Coss. | IG 126273 | Armenia | ICARDA |
| 26 | *Aegilops tauschii* Coss. | IG 126280 | Armenia | ICARDA |
| 27 | *Aegilops tauschii* Coss. | IG 126293 | Armenia | ICARDA |
| 28 | *Aegilops tauschii* Coss. | IG 126353 | Armenia | ICARDA |
| 29 | *Aegilops tauschii* Coss. | IG 126387 | Turkmenistan | ICARDA |
| 30 | *Aegilops tauschii* Coss. | IG 126489 | Turkmenistan | ICARDA |
| 31 | *Aegilops tauschii* Coss. | IG 126991 | Armenia | ICARDA |
| 32 | *Aegilops tauschii* Coss. | IG 127015 | Armenia | ICARDA |
| 33 | *Aegilops tauschii* Coss. | IG 131606 | Kyrgyzstan | ICARDA |
| 34 | *Aegilops tauschii* Coss. | IG 46623 | Syria | ICARDA |
| 35 | *Aegilops tauschii* Coss. | IG 46663 | Pakistan | ICARDA |
| 36 | *Aegilops tauschii* Coss. | IG 46666 | Pakistan | ICARDA |
| 37 | *Aegilops tauschii* Coss. | IG 46682 | Pakistan | ICARDA |
| 38 | *Aegilops tauschii* Coss. | IG 47173 | Armenia | ICARDA |
| 39 | *Aegilops tauschii* Coss. | IG 47182 | Azerbaijan | ICARDA |
| 40 | *Aegilops tauschii* Coss. | IG 47186 | Azerbaijan | ICARDA |
| 41 | *Aegilops tauschii* Coss. | IG 47188 | Azerbaijan | ICARDA |
| 42 | *Aegilops tauschii* Coss. | IG 47192 | Azerbaijan | ICARDA |
| 43 | *Aegilops tauschii* Coss. | IG 47193 | Azerbaijan | ICARDA |
| 44 | *Aegilops tauschii* Coss. | IG 47194 | Azerbaijan | ICARDA |
| 45 | *Aegilops tauschii* Coss. | IG 47196 | Azerbaijan | ICARDA |
| 46 | *Aegilops tauschii* Coss. | IG 47199 | Azerbaijan | ICARDA |
| 47 | *Aegilops tauschii* Coss. | IG 47202 | Azerbaijan | ICARDA |
| 48 | *Aegilops tauschii* Coss. | IG 47203 | Azerbaijan | ICARDA |
| 49 | *Aegilops tauschii* Coss. | IG 47204 | Azerbaijan | ICARDA |
| 50 | *Aegilops tauschii* Coss. | IG 47259 | Syria | ICARDA |
| 51 | *Aegilops tauschii* Coss. | IG 48042 | India | ICARDA |
| 52 | *Aegilops tauschii* Coss. | IG 48274 | Dagestan | ICARDA |
| 53 | *Aegilops tauschii* Coss. | IG 48508 | Turkmenistan | ICARDA |
| 54 | *Aegilops tauschii* Coss. | IG 48518 | Turkmenistan | ICARDA |
| 55 | *Aegilops tauschii* Coss. | IG 48539 | Uzbekistan | ICARDA |
| 56 | *Aegilops tauschii* Coss. | IG 48554 | Tajikistan | ICARDA |
| 57 | *Aegilops tauschii* Coss. | IG 48559 | Tajikistan | ICARDA |
| 58 | *Aegilops tauschii* Coss. | IG 48564 | Tajikistan | ICARDA |
| 59 | *Aegilops tauschii* Coss. | IG 48565 | Uzbekistan | ICARDA |
| 60 | *Aegilops tauschii* Coss. | IG 48567 | Uzbekistan | ICARDA |
| 61 | *Aegilops tauschii* Coss. | IG 48747 | Armenia | ICARDA |
| 62 | *Aegilops tauschii* Coss. | IG 48748 | Armenia | ICARDA |
| 63 | *Aegilops tauschii* Coss. | IG 48758 | Armenia | ICARDA |
| 64 | *Aegilops tauschii* Coss. | IG 49095 | Iran | ICARDA |
| 65 | *Aegilops tauschii* Coss. | KU-20-1 | Dagestan | KYOTO |
| 66 | *Aegilops tauschii* Coss. | KU-20-10 | Iran | KYOTO |
| 67 | *Aegilops tauschii* Coss. | KU-20-6 | Pakistan | KYOTO |
| 68 | *Aegilops tauschii* Coss. | KU-20-7 | Iran | KYOTO |
| 69 | *Aegilops tauschii* Coss. | KU-20-8 | Iran | KYOTO |
| 70 | *Aegilops tauschii* Coss. | KU-20-9 | Iran | KYOTO |
| 71 | *Aegilops tauschii* Coss. | KU-2001 | Pakistan | KYOTO |
| 72 | *Aegilops tauschii* Coss. | KU-2003 | Pakistan | KYOTO |
| 73 | *Aegilops tauschii* Coss. | KU-2006 | Pakistan | KYOTO |
| 74 | *Aegilops tauschii* Coss. | KU-2008 | Pakistan | KYOTO |
| 75 | *Aegilops tauschii* Coss. | KU-2010 | Afghanistan | KYOTO |
| 76 | *Aegilops tauschii* Coss. | KU-2012 | Afghanistan | KYOTO |
| 77 | *Aegilops tauschii* Coss. | KU-2016 | Afghanistan | KYOTO |
| 78 | *Aegilops tauschii* Coss. | KU-2018 | Afghanistan | KYOTO |
| 79 | *Aegilops tauschii* Coss. | KU-2022 | Afghanistan | KYOTO |
| 80 | *Aegilops tauschii* Coss. | KU-2025 | Afghanistan | KYOTO |
| 81 | *Aegilops tauschii* Coss. | KU-2027 | Afghanistan | KYOTO |
| 82 | *Aegilops tauschii* Coss. | KU-2028 | Afghanistan | KYOTO |
| 83 | *Aegilops tauschii* Coss. | KU-2032 | Afghanistan | KYOTO |
| 84 | *Aegilops tauschii* Coss. | KU-2035 | Afghanistan | KYOTO |
| 85 | *Aegilops tauschii* Coss. | KU-2039 | Afghanistan | KYOTO |
| 86 | *Aegilops tauschii* Coss. | KU-2042 | Afghanistan | KYOTO |
| 87 | *Aegilops tauschii* Coss. | KU-2043 | Afghanistan | KYOTO |
| 88 | *Aegilops tauschii* Coss. | KU-2044 | Afghanistan | KYOTO |
| 89 | *Aegilops tauschii* Coss. | KU-2050 | Afghanistan | KYOTO |
| 90 | *Aegilops tauschii* Coss. | KU-2051 | Afghanistan | KYOTO |
| 91 | *Aegilops tauschii* Coss. | KU-2056 | Afghanistan | KYOTO |
| 92 | *Aegilops tauschii* Coss. | KU-2058 | Afghanistan | KYOTO |
| 93 | *Aegilops tauschii* Coss. | KU-2059 | Afghanistan | KYOTO |
| 94 | *Aegilops tauschii* Coss. | KU-2061 | Afghanistan | KYOTO |
| 95 | *Aegilops tauschii* Coss. | KU-2063 | Afghanistan | KYOTO |
| 96 | *Aegilops tauschii* Coss. | KU-2066 | Afghanistan | KYOTO |
| 97 | *Aegilops tauschii* Coss. | KU-2068 | Iran | KYOTO |
| 98 | *Aegilops tauschii* Coss. | KU-2069 | Iran | KYOTO |
| 99 | *Aegilops tauschii* Coss. | KU-2074 | Iran | KYOTO |
| 100 | *Aegilops tauschii* Coss. | KU-2075 | Iran | KYOTO |
| 101 | *Aegilops tauschii* Coss. | KU-2076 | Iran | KYOTO |
| 102 | *Aegilops tauschii* Coss. | KU-2077 | Iran | KYOTO |
| 103 | *Aegilops tauschii* Coss. | KU-2078 | Iran | KYOTO |
| 104 | *Aegilops tauschii* Coss. | KU-2079 | Iran | KYOTO |
| 105 | *Aegilops tauschii* Coss. | KU-2080 | Iran | KYOTO |
| 106 | *Aegilops tauschii* Coss. | KU-2082 | Iran | KYOTO |
| 107 | *Aegilops tauschii* Coss. | KU-2083 | Iran | KYOTO |
| 108 | *Aegilops tauschii* Coss. | KU-2086 | Iran | KYOTO |
| 109 | *Aegilops tauschii* Coss. | KU-2087 | Iran | KYOTO |
| 110 | *Aegilops tauschii* Coss. | KU-2088 | Iran | KYOTO |
| 111 | *Aegilops tauschii* Coss. | KU-2090 | Iran | KYOTO |
| 112 | *Aegilops tauschii* Coss. | KU-2091 | Iran | KYOTO |
| 113 | *Aegilops tauschii* Coss. | KU-2092 | Iran | KYOTO |
| 114 | *Aegilops tauschii* Coss. | KU-2093 | Iran | KYOTO |
| 115 | *Aegilops tauschii* Coss. | KU-2096 | Iran | KYOTO |
| 116 | *Aegilops tauschii* Coss. | KU-2097 | Iran | KYOTO |
| 117 | *Aegilops tauschii* Coss. | KU-2098 | Iran | KYOTO |
| 118 | *Aegilops tauschii* Coss. | KU-2100 | Iran | KYOTO |
| 119 | *Aegilops tauschii* Coss. | KU-2101 | Iran | KYOTO |
| 120 | *Aegilops tauschii* Coss. | KU-2102 | Iran | KYOTO |
| 121 | *Aegilops tauschii* Coss. | KU-2103 | Iran | KYOTO |
| 122 | *Aegilops tauschii* Coss. | KU-2104 | Iran | KYOTO |
| 123 | *Aegilops tauschii* Coss. | KU-2105 | Iran | KYOTO |
| 124 | *Aegilops tauschii* Coss. | KU-2106 | Iran | KYOTO |
| 125 | *Aegilops tauschii* Coss. | KU-2107 | Iran | KYOTO |
| 126 | *Aegilops tauschii* Coss. | KU-2108 | Iran | KYOTO |
| 127 | *Aegilops tauschii* Coss. | KU-2109 | Iran | KYOTO |
| 128 | *Aegilops tauschii* Coss. | KU-2110 | Iran | KYOTO |
| 129 | *Aegilops tauschii* Coss. | KU-2111 | Iran | KYOTO |
| 130 | *Aegilops tauschii* Coss. | KU-2112 | Iran | KYOTO |
| 131 | *Aegilops tauschii* Coss. | KU-2113 | Iran | KYOTO |
| 132 | *Aegilops tauschii* Coss. | KU-2115 | Iran | KYOTO |
| 133 | *Aegilops tauschii* Coss. | KU-2116 | Iran | KYOTO |
| 134 | *Aegilops tauschii* Coss. | KU-2118 | Iran | KYOTO |
| 135 | *Aegilops tauschii* Coss. | KU-2120 | Iran | KYOTO |
| 136 | *Aegilops tauschii* Coss. | KU-2121 | Iran | KYOTO |
| 137 | *Aegilops tauschii* Coss. | KU-2122 | Iran | KYOTO |
| 138 | *Aegilops tauschii* Coss. | KU-2124 | Iran | KYOTO |
| 139 | *Aegilops tauschii* Coss. | KU-2126 | Iran | KYOTO |
| 140 | *Aegilops tauschii* Coss. | KU-2131 | Turkey | KYOTO |
| 141 | *Aegilops tauschii* Coss. | KU-2132 | Turkey | KYOTO |
| 142 | *Aegilops tauschii* Coss. | KU-2133 | Turkey | KYOTO |
| 143 | *Aegilops tauschii* Coss. | KU-2136 | Turkey | KYOTO |
| 144 | *Aegilops tauschii* Coss. | KU-2137 | Turkey | KYOTO |
| 145 | *Aegilops tauschii* Coss. | KU-2138 | Turkey | KYOTO |
| 146 | *Aegilops tauschii* Coss. | KU-2140 | Turkey | KYOTO |
| 147 | *Aegilops tauschii* Coss. | KU-2141 | Turkey | KYOTO |
| 148 | *Aegilops tauschii* Coss. | KU-2142 | Iran | KYOTO |
| 149 | *Aegilops tauschii* Coss. | KU-2143 | Iran | KYOTO |
| 150 | *Aegilops tauschii* Coss. | KU-2144 | Iran | KYOTO |
| 151 | *Aegilops tauschii* Coss. | KU-2145 | Iran | KYOTO |
| 152 | *Aegilops tauschii* Coss. | KU-2148 | Iran | KYOTO |
| 153 | *Aegilops tauschii* Coss. | KU-2149 | Iran | KYOTO |
| 154 | *Aegilops tauschii* Coss. | KU-2150 | Iran | KYOTO |
| 155 | *Aegilops tauschii* Coss. | KU-2151 | Iran | KYOTO |
| 156 | *Aegilops tauschii* Coss. | KU-2152 | Iran | KYOTO |
| 157 | *Aegilops tauschii* Coss. | KU-2153 | Iran | KYOTO |
| 158 | *Aegilops tauschii* Coss. | KU-2154 | Iran | KYOTO |
| 159 | *Aegilops tauschii* Coss. | KU-2155 | Iran | KYOTO |
| 160 | *Aegilops tauschii* Coss. | KU-2156 | Iran | KYOTO |
| 161 | *Aegilops tauschii* Coss. | KU-2157 | Iran | KYOTO |
| 162 | *Aegilops tauschii* Coss. | KU-2158 | Iran | KYOTO |
| 163 | *Aegilops tauschii* Coss. | KU-2159 | Iran | KYOTO |
| 164 | *Aegilops tauschii* Coss. | KU-2160 | Iran | KYOTO |
| 165 | *Aegilops tauschii* Coss. | KU-2612 | Afghanistan | KYOTO |
| 166 | *Aegilops tauschii* Coss. | KU-2617 | Afghanistan | KYOTO |
| 167 | *Aegilops tauschii* Coss. | KU-2619 | Afghanistan | KYOTO |
| 168 | *Aegilops tauschii* Coss. | KU-2621 | Afghanistan | KYOTO |
| 169 | *Aegilops tauschii* Coss. | KU-2624 | Afghanistan | KYOTO |
| 170 | *Aegilops tauschii* Coss. | KU-2627 | Afghanistan | KYOTO |
| 171 | *Aegilops tauschii* Coss. | KU-2630 | Afghanistan | KYOTO |
| 172 | *Aegilops tauschii* Coss. | KU-2632 | Afghanistan | KYOTO |
| 173 | *Aegilops tauschii* Coss. | KU-2633 | Afghanistan | KYOTO |
| 174 | *Aegilops tauschii* Coss. | KU-2635 | Afghanistan | KYOTO |
| 175 | *Aegilops tauschii* Coss. | KU-2636 | Afghanistan | KYOTO |
| 176 | *Aegilops tauschii* Coss. | KU-2638 | Afghanistan | KYOTO |
| 177 | *Aegilops tauschii* Coss. | KU-2639 | Afghanistan | KYOTO |
| 178 | *Aegilops tauschii* Coss. | KU-2801 | Azerbaijan | KYOTO |
| 179 | *Aegilops tauschii* Coss. | KU-2804 | Azerbaijan | KYOTO |
| 180 | *Aegilops tauschii* Coss. | KU-2806 | Azerbaijan | KYOTO |
| 181 | *Aegilops tauschii* Coss. | KU-2809 | Armenia | KYOTO |
| 182 | *Aegilops tauschii* Coss. | KU-2810 | Armenia | KYOTO |
| 183 | *Aegilops tauschii* Coss. | KU-2811 | Armenia | KYOTO |
| 184 | *Aegilops tauschii* Coss. | KU-2814 | Armenia | KYOTO |
| 185 | *Aegilops tauschii* Coss. | KU-2816 | Armenia | KYOTO |
| 186 | *Aegilops tauschii* Coss. | KU-2821 | Armenia | KYOTO |
| 187 | *Aegilops tauschii* Coss. | KU-2822A | Armenia | KYOTO |
| 188 | *Aegilops tauschii* Coss. | KU-2823 | Armenia | KYOTO |
| 189 | *Aegilops tauschii* Coss. | KU-2824 | Armenia | KYOTO |
| 190 | *Aegilops tauschii* Coss. | KU-2826 | Georgia | KYOTO |
| 191 | *Aegilops tauschii* Coss. | KU-2827 | Georgia | KYOTO |
| 192 | *Aegilops tauschii* Coss. | KU-2828 | Georgia | KYOTO |
| 193 | *Aegilops tauschii* Coss. | KU-2829A | Georgia | KYOTO |
| 194 | *Aegilops tauschii* Coss. | KU-2832 | Georgia | KYOTO |
| 195 | *Aegilops tauschii* Coss. | KU-2834 | Georgia | KYOTO |
| 196 | *Aegilops tauschii* Coss. | KU-2835B | Georgia | KYOTO |
| 197 | *Aegilops tauschii* Coss. | KU-2836 | Georgia | KYOTO |
| 198 | *Aegilops tauschii* Coss. | PI 476874 | Afghanistan | USDA |
| 199 | *Aegilops tauschii* Coss. | PI 486267 | Turkey | USDA |
| 200 | *Aegilops tauschii* Coss. | PI 486270 | Turkey | USDA |
| 201 | *Aegilops tauschii* Coss. | PI 486274 | Turkey | USDA |
| 202 | *Aegilops tauschii* Coss. | PI 486277 | Turkey | USDA |
| 203 | *Aegilops tauschii* Coss. | PI 499262 | China | USDA |
| 204 | *Aegilops tauschii* Coss. | PI 508262 | China | USDA |
| 205 | *Aegilops tauschii* Coss. | PI 508264 | China | USDA |
| 206 | *Aegilops tauschii* Coss. | PI 554319 | Turkey | USDA |
| 207 | *Triticum aestivum* L. | KU-152 | China | KYOTO |
| 208 | *Triticum aestivum* L. | KU-161 | - | KYOTO |
| 209 | *Triticum aestivum* L. | KU-162-2 | Pakistan | KYOTO |
| 210 | *Triticum aestivum* L. | KU-166 | China | KYOTO |
| 211 | *Triticum aestivum* L. | KU-192 | - | KYOTO |
| 212 | *Triticum aestivum* L. | KU-197 | Turkey | KYOTO |
| 213 | *Triticum aestivum* L. | KU-265 | Japan | KYOTO |
| 214 | *Triticum aestivum* L. | KU-309 | United States of America | KYOTO |
| 215 | *Triticum aestivum* L. | KU-333 | Canada | KYOTO |
| 216 | *Triticum aestivum* L. | KU-336 | United States of America | KYOTO |
| 217 | *Triticum aestivum* L. | KU-366 | United Kingdom | KYOTO |
| 218 | *Triticum aestivum* L. | KU-370 | United Kingdom | KYOTO |
| 219 | *Triticum aestivum* L. | KU-371 | United Kingdom | KYOTO |
| 220 | *Triticum aestivum* L. | KU-372 | United Kingdom | KYOTO |
| 221 | *Triticum aestivum* L. | KU-373 | United Kingdom | KYOTO |
| 222 | *Triticum aestivum* L. | KU-374 | United Kingdom | KYOTO |
| 223 | *Triticum aestivum* L. | KU-405 | The former Union of Soviet Socialist Republics | KYOTO |
| 224 | *Triticum aestivum* L. | KU-479 | China | KYOTO |
| 225 | *Triticum aestivum* L. | KU-481 | China | KYOTO |
| 226 | *Triticum aestivum* L. | KU-483 | Tanzania | KYOTO |
| 227 | *Triticum aestivum* L. | KU-497 | India | KYOTO |
| 228 | *Triticum aestivum* L. | KU-504 | China | KYOTO |
| 229 | *Triticum aestivum* L. | KU-601 | Japan | KYOTO |
| 230 | *Triticum aestivum* L. | KU-1002 | Spain | KYOTO |
| 231 | *Triticum aestivum* L. | KU-1005 | Spain | KYOTO |
| 232 | *Triticum aestivum* L. | KU-1011 | Spain | KYOTO |
| 233 | *Triticum aestivum* L. | KU-1020 | Spain | KYOTO |
| 234 | *Triticum aestivum* L. | KU-1049 | Spain | KYOTO |
| 235 | *Triticum aestivum* L. | KU-1062 | Spain | KYOTO |
| 236 | *Triticum aestivum* L. | KU-1137 | Spain | KYOTO |
| 237 | *Triticum aestivum* L. | KU-1143 | Spain | KYOTO |
| 238 | *Triticum aestivum* L. | KU-1208 | Japan | KYOTO |
| 239 | *Triticum aestivum* L. | KU-1215 | Japan | KYOTO |
| 240 | *Triticum aestivum* L. | KU-1230 | Japan | KYOTO |
| 241 | *Triticum aestivum* L. | KU-1279 | Japan | KYOTO |
| 242 | *Triticum aestivum* L. | KU-1302 | Greece | KYOTO |
| 243 | *Triticum aestivum* L. | KU-1347 | Greece | KYOTO |
| 244 | *Triticum aestivum* L. | KU-1392 | Romania | KYOTO |
| 245 | *Triticum aestivum* L. | KU-1394 | Romania | KYOTO |
| 246 | *Triticum aestivum* L. | KU-1421 | Romania | KYOTO |
| 247 | *Triticum aestivum* L. | KU-1424 | Romania | KYOTO |
| 248 | *Triticum aestivum* L. | KU-1521 | The former Union of Soviet Socialist Republics | KYOTO |
| 249 | *Triticum aestivum* L. | KU-1527 | The former Union of Soviet Socialist Republics | KYOTO |
| 250 | *Triticum aestivum* L. | KU-1644 | The former Union of Soviet Socialist Republics | KYOTO |
| 251 | *Triticum aestivum* L. | KU-1668 | The former Union of Soviet Socialist Republics | KYOTO |
| 252 | *Triticum aestivum* L. | KU-1697 | The former Union of Soviet Socialist Republics | KYOTO |
| 253 | *Triticum aestivum* L. | KU-1797 | The former Union of Soviet Socialist Republics | KYOTO |
| 254 | *Triticum aestivum* L. | KU-1812 | Georgia | KYOTO |
| 255 | *Triticum aestivum* L. | KU-1814 | Georgia | KYOTO |
| 256 | *Triticum aestivum* L. | KU-1817 | Georgia | KYOTO |
| 257 | *Triticum aestivum* L. | KU-3004 | Pakistan | KYOTO |
| 258 | *Triticum aestivum* L. | KU-3006 | Pakistan | KYOTO |
| 259 | *Triticum aestivum* L. | KU-3010 | Pakistan | KYOTO |
| 260 | *Triticum aestivum* L. | KU-3037 | Pakistan | KYOTO |
| 261 | *Triticum aestivum* L. | KU-3045 | Afghanistan | KYOTO |
| 262 | *Triticum aestivum* L. | KU-3054 | Afghanistan | KYOTO |
| 263 | *Triticum aestivum* L. | KU-3062 | Afghanistan | KYOTO |
| 264 | *Triticum aestivum* L. | KU-3063 | Afganistan | KYOTO |
| 265 | *Triticum aestivum* L. | KU-3083 | Afghanistan | KYOTO |
| 266 | *Triticum aestivum* L. | KU-3089 | Afghanistan | KYOTO |
| 267 | *Triticum aestivum* L. | KU-3097 | Iran | KYOTO |
| 268 | *Triticum aestivum* L. | KU-3098 | Iran | KYOTO |
| 269 | *Triticum aestivum* L. | KU-3121 | Iran | KYOTO |
| 270 | *Triticum aestivum* L. | KU-3126 | Iran | KYOTO |
| 271 | *Triticum aestivum* L. | KU-3136 | Iran | KYOTO |
| 272 | *Triticum aestivum* L. | KU-3162 | Iran | KYOTO |
| 273 | *Triticum aestivum* L. | KU-3184 | Iran | KYOTO |
| 274 | *Triticum aestivum* L. | KU-3189 | Iran | KYOTO |
| 275 | *Triticum aestivum* L. | KU-3202 | Iran | KYOTO |
| 276 | *Triticum aestivum* L. | KU-3232 | Iran | KYOTO |
| 277 | *Triticum aestivum* L. | KU-3236 | Iran | KYOTO |
| 278 | *Triticum aestivum* L. | KU-3242 | Iran | KYOTO |
| 279 | *Triticum aestivum* L. | KU-3274 | Iran | KYOTO |
| 280 | *Triticum aestivum* L. | KU-3289 | Iran | KYOTO |
| 281 | *Triticum aestivum* L. | KU-3299 | Pakistan | KYOTO |
| 282 | *Triticum aestivum* L. | KU-3351 | Pakistan | KYOTO |
| 283 | *Triticum aestivum* L. | KU-3377 | Iran | KYOTO |
| 284 | *Triticum aestivum* L. | KU-3401 | The former German Democratic Republic | KYOTO |
| 285 | *Triticum aestivum* L. | KU-3413 | The former German Democratic Republic | KYOTO |
| 286 | *Triticum aestivum* L. | KU-3416 | The former German Democratic Republic | KYOTO |
| 287 | *Triticum aestivum* L. | KU-3417 | The former German Democratic Republic | KYOTO |
| 288 | *Triticum aestivum* L. | KU-3421 | The former German Democratic Republic | KYOTO |
| 289 | *Triticum aestivum* L. | KU-3443 | The former German Democratic Republic | KYOTO |
| 290 | *Triticum aestivum* L. | KU-3444 | The former German Democratic Republic | KYOTO |
| 291 | *Triticum aestivum* L. | KU-3445 | The former German Democratic Republic | KYOTO |
| 292 | *Triticum aestivum* L. | KU-3752 | Egypt | KYOTO |
| 293 | *Triticum aestivum* L. | KU-3777 | Jordan | KYOTO |
| 294 | *Triticum aestivum* L. | KU-3778 | Lebanon | KYOTO |
| 295 | *Triticum aestivum* L. | KU-3780 | Syria | KYOTO |
| 296 | *Triticum aestivum* L. | KU-3784 | Turkey | KYOTO |
| 297 | *Triticum aestivum* L. | KU-3789 | Turkey | KYOTO |
| 298 | *Triticum aestivum* L. | KU-3801 | Turkey | KYOTO |
| 299 | *Triticum aestivum* L. | KU-3806 | Turkey | KYOTO |
| 300 | *Triticum aestivum* L. | KU-3818 | Turkey | KYOTO |
| 301 | *Triticum aestivum* L. | KU-3834 | Turkey | KYOTO |
| 302 | *Triticum aestivum* L. | KU-3848 | Turkey | KYOTO |
| 303 | *Triticum aestivum* L. | KU-3851 | Turkey | KYOTO |
| 304 | *Triticum aestivum* L. | KU-3857 | Turkey | KYOTO |
| 305 | *Triticum aestivum* L. | KU-3860 | Turkey | KYOTO |
| 306 | *Triticum aestivum* L. | KU-3868 | Italy | KYOTO |
| 307 | *Triticum aestivum* L. | KU-4703 | Nepal | KYOTO |
| 308 | *Triticum aestivum* L. | KU-4714 | Nepal | KYOTO |
| 309 | *Triticum aestivum* L. | KU-4734 | Nepal | KYOTO |
| 310 | *Triticum aestivum* L. | KU-4759 | Nepal | KYOTO |
| 311 | *Triticum aestivum* L. | KU-4769 | Nepal | KYOTO |
| 312 | *Triticum aestivum* L. | KU-4783 | Nepal | KYOTO |
| 313 | *Triticum aestivum* L. | KU-7001 | Bhutan | KYOTO |
| 314 | *Triticum aestivum* L. | KU-7041 | Bhutan | KYOTO |
| 315 | *Triticum aestivum* L. | KU-7113 | Bhutan | KYOTO |
| 316 | *Triticum aestivum* L. | KU-7180 | Bhutan | KYOTO |
| 317 | *Triticum aestivum* L. | KU-7350 | Turkey | KYOTO |
| 318 | *Triticum aestivum* L. | KU-7356 | Ethiopia | KYOTO |
| 319 | *Triticum aestivum* L. | KU-7379 | Ethiopia | KYOTO |
| 320 | *Triticum aestivum* L. | KU-7406 | Ethiopia | KYOTO |
| 321 | *Triticum aestivum* L. | KU-7437 | Afghanistan | KYOTO |
| 322 | *Triticum aestivum* L. | KU-7459 | Afghanistan | KYOTO |
| 323 | *Triticum aestivum* L. | KU-7480 | Afghanistan | KYOTO |
| 324 | *Triticum aestivum* L. | KU-7624 | Afghanistan | KYOTO |
| 325 | *Triticum aestivum* L. | KU-7653 | Afghanistan | KYOTO |
| 326 | *Triticum aestivum* L. | KU-7669 | Afghanistan | KYOTO |
| 327 | *Triticum aestivum* L. | KU-9431 | Ethiopia | KYOTO |
| 328 | *Triticum aestivum* L. | KU-9460 | Ethiopia | KYOTO |
| 329 | *Triticum aestivum* L. | KU-9797 | Ethiopia | KYOTO |
| 330 | *Triticum aestivum* L. | KU-9820 | Ethiopia | KYOTO |
| 331 | *Triticum aestivum* L. | KU-9867 | Ethiopia | KYOTO |
| 332 | *Triticum aestivum* L. | KU-9873 | Ethiopia | KYOTO |
| 333 | *Triticum aestivum* L. | KU-10001 | Iraq | KYOTO |
| 334 | *Triticum aestivum* L. | KU-10154 | Iraq | KYOTO |
| 335 | *Triticum aestivum* L. | KU-10393 | Iran | KYOTO |
| 336 | *Triticum aestivum* L. | KU-10439 | Iran | KYOTO |
| 337 | *Triticum aestivum* L. | KU-10480 | Iran | KYOTO |
| 338 | *Triticum aestivum* L. | KU-10510 | Iran | KYOTO |
| 339 | *Triticum aestivum* L. | KU-11201 | Afghanistan | KYOTO |
| 340 | *Triticum aestivum* L. | KU-11214 | Afghanistan | KYOTO |
| 341 | *Triticum aestivum* L. | KU-11240A | Afghanistan | KYOTO |
| 342 | *Triticum aestivum* L. | KU-11351 | Romania | KYOTO |
| 343 | *Triticum aestivum* L. | KU-11702 | Greece | KYOTO |
| 344 | *Triticum aestivum* L. | KU-11809 | Greece | KYOTO |
| 345 | *Triticum aestivum* L. | KU-11829 | Greece | KYOTO |
| 346 | *Triticum aestivum* L. | KU-13501 | China | KYOTO |
| 347 | *Triticum aestivum* L. | KU-13506 | China | KYOTO |
| 348 | *Triticum aestivum* L. | KU-13546 | China | KYOTO |
| 349 | *Triticum aestivum* L. | KU-13631 | China | KYOTO |
| 350 | *Triticum aestivum* L. | KU-13662 | China | KYOTO |
| 351 | *Triticum aestivum* L. | KU-13708 | China | KYOTO |
| 352 | *Triticum aestivum* L. | KU-13807 | China | KYOTO |
| 353 | *Triticum aestivum* L. | KU-13891 | China | KYOTO |
| 354 | *Triticum aestivum* L. | Abukumawase (winter type) | Japan | NBRP |
| 355 | *Triticum aestivum* L. | Akadaruma | Japan | NBRP |
| 356 | *Triticum aestivum* L. | Ayahikari | Japan | NBRP |
| 357 | *Triticum aestivum* L. | Bobwhite | Mexico | NBRP |
| 358 | *Triticum aestivum* L. | Cheyenne | United States of America | NBRP |
| 359 | *Triticum aestivum* L. | Chihokukomugi | Japan | NBRP |
| 360 | *Triticum aestivum* L. | Chikugoizumi | Japan | NBRP |
| 361 | *Triticum aestivum* L. | Chinese Spring | China | NBRP |
| 362 | *Triticum aestivum* L. | Chogokuwase | Japan | NBRP |
| 363 | *Triticum aestivum* L. | Fujimikomugi | Japan | NBRP |
| 364 | *Triticum aestivum* L. | Gamenya | Autralia | NBRP |
| 365 | *Triticum aestivum* L. | Hanamanten | Japan | NBRP |
| 366 | *Triticum aestivum* L. | Haruyokoi | Japan | NBRP |
| 367 | *Triticum aestivum* L. | Hokkai 240 | Japan | NBRP |
| 368 | *Triticum aestivum* L. | Hope | United States of America | NBRP |
| 369 | *Triticum aestivum* L. | Iwainodaichi | Japan | NBRP |
| 370 | *Triticum aestivum* L. | Kanto 107 | Japan | NBRP |
| 371 | *Triticum aestivum* L. | Kinuiroha | Japan | NBRP |
| 372 | *Triticum aestivum* L. | Kitakamikomugi | Japan | NBRP |
| 373 | *Triticum aestivum* L. | Kitanokaori | Japan | NBRP |
| 374 | *Triticum aestivum* L. | KS831987 | United States of America | NBRP |
| 375 | *Triticum aestivum* L. | Minaminokaori | Japan | NBRP |
| 376 | *Triticum aestivum* L. | Minaminokomugi | Japan | NBRP |
| 377 | *Triticum aestivum* L. | Nambukomugi | Japan | NBRP |
| 378 | *Triticum aestivum* L. | Nebarigoshi | Japan | NBRP |
| 379 | *Triticum aestivum* L. | Nishikazekomugi | Japan | NBRP |
| 380 | *Triticum aestivum* L. | Nobeokabozykomugi | Japan | NBRP |
| 381 | *Triticum aestivum* L. | Norin 26 | Japan | NBRP |
| 382 | *Triticum aestivum* L. | Norin 61 | Japan | NBRP |
| 383 | *Triticum aestivum* L. | Opata 85 | Mexico | NBRP |
| 384 | *Triticum aestivum* L. | Saikai 165 | Japan | NBRP |
| 385 | *Triticum aestivum* L. | Saikai 193 | Japan | NBRP |
| 386 | *Triticum aestivum* L. | Shiroganekomugi | Japan | NBRP |
| 387 | *Triticum aestivum* L. | Shyunyou | Japan | NBRP |
| 388 | *Triticum aestivum* L. | Sumai #3 | China | NBRP |
| 389 | *Triticum aestivum* L. | Synthetic W7984 | - | NBRP |
| 390 | *Triticum aestivum* L. | Tamaizumi | Japan | NBRP |
| 391 | *Triticum aestivum* L. | Timstein | United States of America | NBRP |
| 392 | *Triticum aestivum* L. | U24 | China | NBRP |
| 393 | *Triticum aestivum* L. | Variety duhamerianum | - | NBRP |
| 394 | *Triticum aestivum* L. | Zenkojikomugi | Japan | NBRP |
