# Supplementary material for: Genetic Basis for Spontaneous Hybrid Genome Doubling during Allopolyploid Speciation of Common Wheat Shown by Natural Variation Analyses of the Paternal Species
Source: PLoS One. 2013 Aug 8;8(8):e68310. doi: 10.1371/journal.pone.0068310 (PMC3738567; doi:10.1371/journal.pone.0068310)
Supplement: Table S2 — Principal component scores used to provide Figure 1. The lineage classification is provided for the Ae . tauschii accessions. A hyphen indicates that the information is not available. (DOCX) [file pone.0068310.s004.docx]

Table S2. Principal component scores used to provide Figure 1.

| No. | Species | Accession | Origin | PC1 score | PC2 score | Lineage |
| --- | --- | --- | --- | --- | --- | --- |
| 1 | *Aegilops tauschii* Coss. | AE 1038 | Tajikistan | -4.66 | -1.05 | TauL1 |
| 2 | *Aegilops tauschii* Coss. | AE 1090 | Kazakhstan | -4.87 | -1.03 | TauL1 |
| 3 | *Aegilops tauschii* Coss. | AE 933 | Georgia | -4.66 | -0.80 | TauL1 |
| 4 | *Aegilops tauschii* Coss. | AT 47 | China | -4.58 | -0.86 | TauL1 |
| 5 | *Aegilops tauschii* Coss. | AT 55 | China | -4.51 | -1.06 | TauL1 |
| 6 | *Aegilops tauschii* Coss. | AT 60 | China | -4.57 | -1.15 | TauL1 |
| 7 | *Aegilops tauschii* Coss. | AT 76 | China | -4.57 | -0.93 | TauL1 |
| 8 | *Aegilops tauschii* Coss. | AT 80 | China | -4.51 | -1.14 | TauL1 |
| 9 | *Aegilops tauschii* Coss. | CGN 10734 | Armenia | -4.76 | -0.95 | TauL1 |
| 10 | *Aegilops tauschii* Coss. | CGN 10767 | Pakistan | -5.00 | -1.01 | TauL1 |
| 11 | *Aegilops tauschii* Coss. | CGN 10768 | Pakistan | -4.78 | -1.17 | TauL1 |
| 12 | *Aegilops tauschii* Coss. | CGN 10769 | Pakistan | -5.00 | -1.30 | TauL1 |
| 13 | *Aegilops tauschii* Coss. | CGN 10770 | Pakistan | -4.86 | -1.10 | TauL1 |
| 14 | *Aegilops tauschii* Coss. | CGN 10771 | Pakistan | -4.90 | -1.29 | TauL1 |
| 15 | *Aegilops tauschii* Coss. | IG 108561 | Pakistan | -4.95 | -1.16 | TauL1 |
| 16 | *Aegilops tauschii* Coss. | IG 120735 | Turkmenistan | -4.89 | -0.67 | TauL1 |
| 17 | *Aegilops tauschii* Coss. | IG 120736 | Uzbekistan | -5.25 | -1.21 | TauL1 |
| 18 | *Aegilops tauschii* Coss. | IG 123910 | Uzbekistan | -5.14 | -0.63 | TauL1 |
| 19 | *Aegilops tauschii* Coss. | IG 126273 | Armenia | -4.09 | -0.56 | TauL1 |
| 20 | *Aegilops tauschii* Coss. | IG 126280 | Armenia | -4.69 | -0.43 | TauL1 |
| 21 | *Aegilops tauschii* Coss. | IG 126293 | Armenia | -4.60 | -0.41 | TauL1 |
| 22 | *Aegilops tauschii* Coss. | IG 126353 | Armenia | -4.93 | -0.90 | TauL1 |
| 23 | *Aegilops tauschii* Coss. | IG 126387 | Turkmenistan | -4.80 | -0.91 | TauL1 |
| 24 | *Aegilops tauschii* Coss. | IG 126489 | Turkmenistan | -4.66 | -0.72 | TauL1 |
| 25 | *Aegilops tauschii* Coss. | IG 127015 | Armenia | -5.19 | -0.88 | TauL1 |
| 26 | *Aegilops tauschii* Coss. | IG 131606 | Kyrgyzstan | -5.24 | -0.79 | TauL1 |
| 27 | *Aegilops tauschii* Coss. | IG 46663 | Pakistan | -4.90 | -1.29 | TauL1 |
| 28 | *Aegilops tauschii* Coss. | IG 46666 | Pakistan | -4.94 | -0.92 | TauL1 |
| 29 | *Aegilops tauschii* Coss. | IG 46682 | Pakistan | -4.86 | -1.10 | TauL1 |
| 30 | *Aegilops tauschii* Coss. | IG 47196 | Azerbaijan | -4.42 | -0.71 | TauL1 |
| 31 | *Aegilops tauschii* Coss. | IG 47259 | Syria | -4.75 | -0.85 | TauL1 |
| 32 | *Aegilops tauschii* Coss. | IG 48042 | India | -5.02 | -0.72 | TauL1 |
| 33 | *Aegilops tauschii* Coss. | IG 48508 | Turkmenistan | -4.81 | -1.53 | TauL1 |
| 34 | *Aegilops tauschii* Coss. | IG 48518 | Turkmenistan | -4.65 | -0.94 | TauL1 |
| 35 | *Aegilops tauschii* Coss. | IG 48539 | Uzbekistan | -4.67 | -1.03 | TauL1 |
| 36 | *Aegilops tauschii* Coss. | IG 48554 | Tajikistan | -5.13 | -1.11 | TauL1 |
| 37 | *Aegilops tauschii* Coss. | IG 48559 | Tajikistan | -5.01 | -0.84 | TauL1 |
| 38 | *Aegilops tauschii* Coss. | IG 48564 | Tajikistan | -4.65 | -0.89 | TauL1 |
| 39 | *Aegilops tauschii* Coss. | IG 48565 | Uzbekistan | -5.13 | -0.63 | TauL1 |
| 40 | *Aegilops tauschii* Coss. | IG 48567 | Uzbekistan | -5.19 | -0.79 | TauL1 |
| 41 | *Aegilops tauschii* Coss. | IG 48747 | Armenia | -4.85 | -0.92 | TauL1 |
| 42 | *Aegilops tauschii* Coss. | IG 48748 | Armenia | -4.79 | -0.64 | TauL1 |
| 43 | *Aegilops tauschii* Coss. | IG 48758 | Armenia | -4.71 | -0.95 | TauL1 |
| 44 | *Aegilops tauschii* Coss. | IG 49095 | Iran | -4.81 | -0.42 | TauL1 |
| 45 | *Aegilops tauschii* Coss. | KU-20-6 | Pakistan | -5.09 | -0.93 | TauL1 |
| 46 | *Aegilops tauschii* Coss. | KU-2001 | Pakistan | -4.90 | -1.23 | TauL1 |
| 47 | *Aegilops tauschii* Coss. | KU-2003 | Pakistan | -5.01 | -1.14 | TauL1 |
| 48 | *Aegilops tauschii* Coss. | KU-2006 | Pakistan | -5.00 | -1.35 | TauL1 |
| 49 | *Aegilops tauschii* Coss. | KU-2008 | Pakistan | -4.49 | -1.09 | TauL1 |
| 50 | *Aegilops tauschii* Coss. | KU-2010 | Afghanistan | -5.02 | -1.04 | TauL1 |
| 51 | *Aegilops tauschii* Coss. | KU-2012 | Afghanistan | -4.94 | -1.10 | TauL1 |
| 52 | *Aegilops tauschii* Coss. | KU-2016 | Afghanistan | -5.06 | -1.31 | TauL1 |
| 53 | *Aegilops tauschii* Coss. | KU-2018 | Afghanistan | -4.91 | -1.16 | TauL1 |
| 54 | *Aegilops tauschii* Coss. | KU-2022 | Afghanistan | -4.88 | -1.05 | TauL1 |
| 55 | *Aegilops tauschii* Coss. | KU-2025 | Afghanistan | -4.90 | -0.81 | TauL1 |
| 56 | *Aegilops tauschii* Coss. | KU-2027 | Afghanistan | -5.27 | -0.94 | TauL1 |
| 57 | *Aegilops tauschii* Coss. | KU-2028 | Afghanistan | -4.98 | -0.86 | TauL1 |
| 58 | *Aegilops tauschii* Coss. | KU-2032 | Afghanistan | -4.66 | -0.81 | TauL1 |
| 59 | *Aegilops tauschii* Coss. | KU-2035 | Afghanistan | -4.92 | -0.74 | TauL1 |
| 60 | *Aegilops tauschii* Coss. | KU-2039 | Afghanistan | -4.76 | -1.26 | TauL1 |
| 61 | *Aegilops tauschii* Coss. | KU-2042 | Afghanistan | -4.75 | -1.02 | TauL1 |
| 62 | *Aegilops tauschii* Coss. | KU-2043 | Afghanistan | -4.97 | -1.30 | TauL1 |
| 63 | *Aegilops tauschii* Coss. | KU-2044 | Afghanistan | -4.71 | -1.25 | TauL1 |
| 64 | *Aegilops tauschii* Coss. | KU-2050 | Afghanistan | -4.99 | -1.49 | TauL1 |
| 65 | *Aegilops tauschii* Coss. | KU-2051 | Afghanistan | -5.04 | -1.32 | TauL1 |
| 66 | *Aegilops tauschii* Coss. | KU-2056 | Afghanistan | -5.02 | -0.92 | TauL1 |
| 67 | *Aegilops tauschii* Coss. | KU-2058 | Afghanistan | -5.14 | -1.06 | TauL1 |
| 68 | *Aegilops tauschii* Coss. | KU-2059 | Afghanistan | -5.21 | -1.03 | TauL1 |
| 69 | *Aegilops tauschii* Coss. | KU-2061 | Afghanistan | -4.73 | -1.10 | TauL1 |
| 70 | *Aegilops tauschii* Coss. | KU-2063 | Afghanistan | -4.82 | -0.66 | TauL1 |
| 71 | *Aegilops tauschii* Coss. | KU-2066 | Afghanistan | -4.47 | -1.50 | TauL1 |
| 72 | *Aegilops tauschii* Coss. | KU-2068 | Iran | -4.86 | -0.49 | TauL1 |
| 73 | *Aegilops tauschii* Coss. | KU-2082 | Iran | -4.82 | -0.78 | TauL1 |
| 74 | *Aegilops tauschii* Coss. | KU-2087 | Iran | -4.78 | -0.69 | TauL1 |
| 75 | *Aegilops tauschii* Coss. | KU-2113 | Iran | -4.77 | -1.34 | TauL1 |
| 76 | *Aegilops tauschii* Coss. | KU-2115 | Iran | -4.38 | -0.42 | TauL1 |
| 77 | *Aegilops tauschii* Coss. | KU-2116 | Iran | -4.87 | -0.97 | TauL1 |
| 78 | *Aegilops tauschii* Coss. | KU-2120 | Iran | -4.80 | -0.65 | TauL1 |
| 79 | *Aegilops tauschii* Coss. | KU-2121 | Iran | -4.57 | -0.91 | TauL1 |
| 80 | *Aegilops tauschii* Coss. | KU-2122 | Iran | -4.28 | 0.16 | TauL1 |
| 81 | *Aegilops tauschii* Coss. | KU-2131 | Turkey | -4.70 | -0.90 | TauL1 |
| 82 | *Aegilops tauschii* Coss. | KU-2132 | Turkey | -4.67 | -1.07 | TauL1 |
| 83 | *Aegilops tauschii* Coss. | KU-2133 | Turkey | -4.48 | -0.73 | TauL1 |
| 84 | *Aegilops tauschii* Coss. | KU-2136 | Turkey | -4.62 | -1.06 | TauL1 |
| 85 | *Aegilops tauschii* Coss. | KU-2137 | Turkey | -4.47 | -1.23 | TauL1 |
| 86 | *Aegilops tauschii* Coss. | KU-2138 | Turkey | -4.63 | -1.35 | TauL1 |
| 87 | *Aegilops tauschii* Coss. | KU-2140 | Turkey | -4.64 | -1.40 | TauL1 |
| 88 | *Aegilops tauschii* Coss. | KU-2141 | Turkey | -4.55 | -1.33 | TauL1 |
| 89 | *Aegilops tauschii* Coss. | KU-2142 | Iran | -4.44 | -1.48 | TauL1 |
| 90 | *Aegilops tauschii* Coss. | KU-2143 | Iran | -4.48 | -0.90 | TauL1 |
| 91 | *Aegilops tauschii* Coss. | KU-2144 | Iran | -4.77 | -0.73 | TauL1 |
| 92 | *Aegilops tauschii* Coss. | KU-2145 | Iran | -4.87 | -0.44 | TauL1 |
| 93 | *Aegilops tauschii* Coss. | KU-2148 | Iran | -4.76 | -0.26 | TauL1 |
| 94 | *Aegilops tauschii* Coss. | KU-2149 | Iran | -4.52 | -0.74 | TauL1 |
| 95 | *Aegilops tauschii* Coss. | KU-2150 | Iran | -4.65 | -0.98 | TauL1 |
| 96 | *Aegilops tauschii* Coss. | KU-2151 | Iran | -4.10 | -0.89 | TauL1 |
| 97 | *Aegilops tauschii* Coss. | KU-2152 | Iran | -4.89 | -0.58 | TauL1 |
| 98 | *Aegilops tauschii* Coss. | KU-2153 | Iran | -5.05 | -0.43 | TauL1 |
| 99 | *Aegilops tauschii* Coss. | KU-2154 | Iran | -4.35 | -0.98 | TauL1 |
| 100 | *Aegilops tauschii* Coss. | KU-2157 | Iran | -4.68 | -1.14 | TauL1 |
| 101 | *Aegilops tauschii* Coss. | KU-2612 | Afghanistan | -4.83 | -1.54 | TauL1 |
| 102 | *Aegilops tauschii* Coss. | KU-2617 | Afghanistan | -5.18 | -0.79 | TauL1 |
| 103 | *Aegilops tauschii* Coss. | KU-2619 | Afghanistan | -4.74 | -0.86 | TauL1 |
| 104 | *Aegilops tauschii* Coss. | KU-2621 | Afghanistan | -5.01 | -0.61 | TauL1 |
| 105 | *Aegilops tauschii* Coss. | KU-2624 | Afghanistan | -5.02 | -0.85 | TauL1 |
| 106 | *Aegilops tauschii* Coss. | KU-2627 | Afghanistan | -5.04 | -0.99 | TauL1 |
| 107 | *Aegilops tauschii* Coss. | KU-2630 | Afghanistan | -5.08 | -0.96 | TauL1 |
| 108 | *Aegilops tauschii* Coss. | KU-2632 | Afghanistan | -5.40 | -0.18 | TauL1 |
| 109 | *Aegilops tauschii* Coss. | KU-2633 | Afghanistan | -5.02 | -0.74 | TauL1 |
| 110 | *Aegilops tauschii* Coss. | KU-2635 | Afghanistan | -5.17 | -0.82 | TauL1 |
| 111 | *Aegilops tauschii* Coss. | KU-2636 | Afghanistan | -5.11 | -0.91 | TauL1 |
| 112 | *Aegilops tauschii* Coss. | KU-2638 | Afghanistan | -5.21 | -0.77 | TauL1 |
| 113 | *Aegilops tauschii* Coss. | KU-2639 | Afghanistan | -5.00 | -0.82 | TauL1 |
| 114 | *Aegilops tauschii* Coss. | KU-2809 | Armenia | -4.58 | -0.28 | TauL1 |
| 115 | *Aegilops tauschii* Coss. | KU-2810 | Armenia | -4.70 | -0.80 | TauL1 |
| 116 | *Aegilops tauschii* Coss. | KU-2814 | Armenia | -4.58 | -0.84 | TauL1 |
| 117 | *Aegilops tauschii* Coss. | KU-2816 | Armenia | -4.54 | -0.88 | TauL1 |
| 118 | *Aegilops tauschii* Coss. | KU-2821 | Armenia | -4.21 | -0.86 | TauL1 |
| 119 | *Aegilops tauschii* Coss. | KU-2822A | Armenia | -4.46 | -1.18 | TauL1 |
| 120 | *Aegilops tauschii* Coss. | KU-2823 | Armenia | -4.82 | -1.09 | TauL1 |
| 121 | *Aegilops tauschii* Coss. | KU-2824 | Armenia | -4.69 | -0.75 | TauL1 |
| 122 | *Aegilops tauschii* Coss. | KU-2826 | Georgia | -4.64 | -0.79 | TauL1 |
| 123 | *Aegilops tauschii* Coss. | KU-2828 | Georgia | -4.29 | -0.91 | TauL1 |
| 124 | *Aegilops tauschii* Coss. | KU-2834 | Georgia | -4.57 | -0.64 | TauL1 |
| 125 | *Aegilops tauschii* Coss. | KU-2836 | Georgia | -4.32 | -0.85 | TauL1 |
| 126 | *Aegilops tauschii* Coss. | PI 476874 | Afghanistan | -4.67 | -1.38 | TauL1 |
| 127 | *Aegilops tauschii* Coss. | PI 486270 | Turkey | -4.70 | -1.17 | TauL1 |
| 128 | *Aegilops tauschii* Coss. | PI 486274 | Turkey | -4.53 | -0.50 | TauL1 |
| 129 | *Aegilops tauschii* Coss. | PI 486277 | Turkey | -4.56 | -1.08 | TauL1 |
| 130 | *Aegilops tauschii* Coss. | PI 499262 | China | -5.08 | -0.87 | TauL1 |
| 131 | *Aegilops tauschii* Coss. | PI 508262 | China | -4.80 | -0.70 | TauL1 |
| 132 | *Aegilops tauschii* Coss. | PI 508264 | China | -4.49 | -1.14 | TauL1 |
| 133 | *Aegilops tauschii* Coss. | PI 554319 | Turkey | -4.65 | -0.88 | TauL1 |
| 134 | *Aegilops tauschii* Coss. | AE 1037 | Georgia | -0.64 | 3.48 | TauL2 |
| 135 | *Aegilops tauschii* Coss. | IG 120863 | Dagestan | -0.58 | 3.56 | TauL2 |
| 136 | *Aegilops tauschii* Coss. | IG 120866 | Dagestan | -0.14 | 3.68 | TauL2 |
| 137 | *Aegilops tauschii* Coss. | IG 126991 | Armenia | -1.09 | 3.49 | TauL2 |
| 138 | *Aegilops tauschii* Coss. | IG 46623 | Syria | -0.85 | 3.85 | TauL2 |
| 139 | *Aegilops tauschii* Coss. | IG 47173 | Armenia | 0.06 | 3.11 | TauL2 |
| 140 | *Aegilops tauschii* Coss. | IG 47182 | Azerbaijan | -0.21 | 3.72 | TauL2 |
| 141 | *Aegilops tauschii* Coss. | IG 47186 | Azerbaijan | -0.68 | 3.78 | TauL2 |
| 142 | *Aegilops tauschii* Coss. | IG 47188 | Azerbaijan | 0.37 | 2.93 | TauL2 |
| 143 | *Aegilops tauschii* Coss. | IG 47192 | Azerbaijan | 0.12 | 3.71 | TauL2 |
| 144 | *Aegilops tauschii* Coss. | IG 47193 | Azerbaijan | -0.55 | 3.73 | TauL2 |
| 145 | *Aegilops tauschii* Coss. | IG 47194 | Azerbaijan | -0.63 | 3.36 | TauL2 |
| 146 | *Aegilops tauschii* Coss. | IG 47199 | Azerbaijan | -0.97 | 3.72 | TauL2 |
| 147 | *Aegilops tauschii* Coss. | IG 47202 | Azerbaijan | -0.19 | 1.40 | TauL2 |
| 148 | *Aegilops tauschii* Coss. | IG 47203 | Azerbaijan | -1.13 | 3.85 | TauL2 |
| 149 | *Aegilops tauschii* Coss. | IG 47204 | Azerbaijan | -1.35 | 4.01 | TauL2 |
| 150 | *Aegilops tauschii* Coss. | IG 48274 | Dagestan | -0.87 | 3.95 | TauL2 |
| 151 | *Aegilops tauschii* Coss. | KU-20-1 | Dagestan | -0.93 | 3.91 | TauL2 |
| 152 | *Aegilops tauschii* Coss. | KU-20-10 | Iran | 0.65 | 3.09 | TauL2 |
| 153 | *Aegilops tauschii* Coss. | KU-20-7 | Iran | -0.92 | 3.64 | TauL2 |
| 154 | *Aegilops tauschii* Coss. | KU-20-8 | Iran | -0.87 | 3.47 | TauL2 |
| 155 | *Aegilops tauschii* Coss. | KU-20-9 | Iran | -0.75 | 4.58 | TauL2 |
| 156 | *Aegilops tauschii* Coss. | KU-2069 | Iran | -0.44 | 3.51 | TauL2 |
| 157 | *Aegilops tauschii* Coss. | KU-2074 | Iran | -0.69 | 4.01 | TauL2 |
| 158 | *Aegilops tauschii* Coss. | KU-2075 | Iran | -0.84 | 4.07 | TauL2 |
| 159 | *Aegilops tauschii* Coss. | KU-2076 | Iran | -0.39 | 3.85 | TauL2 |
| 160 | *Aegilops tauschii* Coss. | KU-2077 | Iran | -0.85 | 4.16 | TauL2 |
| 161 | *Aegilops tauschii* Coss. | KU-2078 | Iran | -0.33 | 3.92 | TauL2 |
| 162 | *Aegilops tauschii* Coss. | KU-2079 | Iran | -1.19 | 4.34 | TauL2 |
| 163 | *Aegilops tauschii* Coss. | KU-2080 | Iran | -1.00 | 3.62 | TauL2 |
| 164 | *Aegilops tauschii* Coss. | KU-2083 | Iran | -0.43 | 3.34 | TauL2 |
| 165 | *Aegilops tauschii* Coss. | KU-2086 | Iran | -0.67 | 3.79 | TauL2 |
| 166 | *Aegilops tauschii* Coss. | KU-2088 | Iran | -1.39 | 4.21 | TauL2 |
| 167 | *Aegilops tauschii* Coss. | KU-2090 | Iran | -1.56 | 3.95 | TauL2 |
| 168 | *Aegilops tauschii* Coss. | KU-2091 | Iran | -0.84 | 3.75 | TauL2 |
| 169 | *Aegilops tauschii* Coss. | KU-2092 | Iran | -0.29 | 3.96 | TauL2 |
| 170 | *Aegilops tauschii* Coss. | KU-2093 | Iran | -0.91 | 3.54 | TauL2 |
| 171 | *Aegilops tauschii* Coss. | KU-2096 | Iran | -1.10 | 4.01 | TauL2 |
| 172 | *Aegilops tauschii* Coss. | KU-2097 | Iran | -0.86 | 3.91 | TauL2 |
| 173 | *Aegilops tauschii* Coss. | KU-2098 | Iran | -0.85 | 3.73 | TauL2 |
| 174 | *Aegilops tauschii* Coss. | KU-2100 | Iran | 0.04 | 3.69 | TauL2 |
| 175 | *Aegilops tauschii* Coss. | KU-2101 | Iran | -0.66 | 3.44 | TauL2 |
| 176 | *Aegilops tauschii* Coss. | KU-2102 | Iran | -0.35 | 3.67 | TauL2 |
| 177 | *Aegilops tauschii* Coss. | KU-2103 | Iran | -0.32 | 2.94 | TauL2 |
| 178 | *Aegilops tauschii* Coss. | KU-2104 | Iran | 0.44 | 3.58 | TauL2 |
| 179 | *Aegilops tauschii* Coss. | KU-2105 | Iran | 0.16 | 2.53 | TauL2 |
| 180 | *Aegilops tauschii* Coss. | KU-2106 | Iran | 0.03 | 3.57 | TauL2 |
| 181 | *Aegilops tauschii* Coss. | KU-2107 | Iran | -0.35 | 2.98 | TauL2 |
| 182 | *Aegilops tauschii* Coss. | KU-2108 | Iran | -0.11 | 2.98 | TauL2 |
| 183 | *Aegilops tauschii* Coss. | KU-2109 | Iran | 0.03 | 3.57 | TauL2 |
| 184 | *Aegilops tauschii* Coss. | KU-2110 | Iran | -0.19 | 3.53 | TauL2 |
| 185 | *Aegilops tauschii* Coss. | KU-2111 | Iran | -1.32 | 3.48 | TauL2 |
| 186 | *Aegilops tauschii* Coss. | KU-2112 | Iran | -1.08 | 3.71 | TauL2 |
| 187 | *Aegilops tauschii* Coss. | KU-2118 | Iran | -0.11 | 4.28 | TauL2 |
| 188 | *Aegilops tauschii* Coss. | KU-2124 | Iran | -0.41 | 3.59 | TauL2 |
| 189 | *Aegilops tauschii* Coss. | KU-2126 | Iran | -0.40 | 3.88 | TauL2 |
| 190 | *Aegilops tauschii* Coss. | KU-2155 | Iran | -0.24 | 4.45 | TauL2 |
| 191 | *Aegilops tauschii* Coss. | KU-2156 | Iran | -0.09 | 4.35 | TauL2 |
| 192 | *Aegilops tauschii* Coss. | KU-2158 | Iran | -0.79 | 2.23 | TauL2 |
| 193 | *Aegilops tauschii* Coss. | KU-2159 | Iran | -0.62 | 2.39 | TauL2 |
| 194 | *Aegilops tauschii* Coss. | KU-2160 | Iran | -0.50 | 2.27 | TauL2 |
| 195 | *Aegilops tauschii* Coss. | KU-2801 | Azerbaijan | -0.70 | 3.64 | TauL2 |
| 196 | *Aegilops tauschii* Coss. | KU-2804 | Azerbaijan | -0.92 | 3.99 | TauL2 |
| 197 | *Aegilops tauschii* Coss. | KU-2806 | Azerbaijan | -0.40 | 3.87 | TauL2 |
| 198 | *Aegilops tauschii* Coss. | KU-2811 | Armenia | -0.33 | 4.44 | TauL2 |
| 199 | *Aegilops tauschii* Coss. | KU-2827 | Georgia | -0.51 | 3.52 | TauL2 |
| 200 | *Aegilops tauschii* Coss. | KU-2835B | Georgia | -0.71 | 2.56 | TauL2 |
| 201 | *Aegilops tauschii* Coss. | PI 486267 | Turkey | -0.81 | 3.99 | TauL2 |
| 202 | *Aegilops tauschii* Coss. | AE 454 | Georgia | -1.46 | -0.48 | TauL3 |
| 203 | *Aegilops tauschii* Coss. | AE 457 | Georgia | -1.54 | -0.61 | TauL3 |
| 204 | *Aegilops tauschii* Coss. | AE 929 | Georgia | -1.86 | -0.52 | TauL3 |
| 205 | *Aegilops tauschii* Coss. | KU-2829A | Georgia | -1.65 | -0.17 | TauL3 |
| 206 | *Aegilops tauschii* Coss. | KU-2832 | Georgia | -1.66 | 0.20 | TauL3 |
| 207 | *Triticum aestivum* L. | KU-152 | China | 3.56 | -0.66 | - |
| 208 | *Triticum aestivum* L. | KU-161 | - | 3.06 | -0.53 | - |
| 209 | *Triticum aestivum* L. | KU-162-2 | Pakistan | 3.54 | -0.66 | - |
| 210 | *Triticum aestivum* L. | KU-166 | China | 4.11 | -0.35 | - |
| 211 | *Triticum aestivum* L. | KU-192 | - | 3.35 | -0.91 | - |
| 212 | *Triticum aestivum* L. | KU-197 | Turkey | 3.69 | 0.15 | - |
| 213 | *Triticum aestivum* L. | KU-265 | Japan | 4.07 | -1.07 | - |
| 214 | *Triticum aestivum* L. | KU-309 | United States of America | 3.20 | -0.33 | - |
| 215 | *Triticum aestivum* L. | KU-333 | Canada | 2.95 | -0.98 | - |
| 216 | *Triticum aestivum* L. | KU-336 | United States of America | 3.47 | -0.95 | - |
| 217 | *Triticum aestivum* L. | KU-366 | United Kingdom | 3.55 | -1.11 | - |
| 218 | *Triticum aestivum* L. | KU-370 | United Kingdom | 3.57 | -1.41 | - |
| 219 | *Triticum aestivum* L. | KU-371 | United Kingdom | 3.99 | -1.07 | - |
| 220 | *Triticum aestivum* L. | KU-372 | United Kingdom | 3.45 | -0.91 | - |
| 221 | *Triticum aestivum* L. | KU-373 | United Kingdom | 2.95 | -1.29 | - |
| 222 | *Triticum aestivum* L. | KU-374 | United Kingdom | 3.56 | -0.93 | - |
| 223 | *Triticum aestivum* L. | KU-405 | The former Union of Soviet Socialist Republics | 3.73 | -0.87 | - |
| 224 | *Triticum aestivum* L. | KU-479 | China | 3.43 | -1.04 | - |
| 225 | *Triticum aestivum* L. | KU-481 | China | 3.69 | -0.89 | - |
| 226 | *Triticum aestivum* L. | KU-483 | Tanzania | 3.93 | -1.17 | - |
| 227 | *Triticum aestivum* L. | KU-497 | India | 3.32 | -0.57 | - |
| 228 | *Triticum aestivum* L. | KU-504 | China | 3.94 | -0.38 | - |
| 229 | *Triticum aestivum* L. | KU-601 | Japan | 4.18 | -0.38 | - |
| 230 | *Triticum aestivum* L. | KU-1002 | Spain | 3.22 | -0.62 | - |
| 231 | *Triticum aestivum* L. | KU-1005 | Spain | 3.68 | -0.62 | - |
| 232 | *Triticum aestivum* L. | KU-1011 | Spain | 4.06 | -0.70 | - |
| 233 | *Triticum aestivum* L. | KU-1020 | Spain | 4.18 | -0.40 | - |
| 234 | *Triticum aestivum* L. | KU-1049 | Spain | 3.77 | -1.36 | - |
| 235 | *Triticum aestivum* L. | KU-1062 | Spain | 3.94 | -0.27 | - |
| 236 | *Triticum aestivum* L. | KU-1137 | Spain | 3.65 | -0.31 | - |
| 237 | *Triticum aestivum* L. | KU-1143 | Spain | 4.22 | -1.07 | - |
| 238 | *Triticum aestivum* L. | KU-1208 | Japan | 3.42 | -1.05 | - |
| 239 | *Triticum aestivum* L. | KU-1215 | Japan | 3.85 | -1.27 | - |
| 240 | *Triticum aestivum* L. | KU-1230 | Japan | 3.90 | -0.71 | - |
| 241 | *Triticum aestivum* L. | KU-1279 | Japan | 3.66 | -0.83 | - |
| 242 | *Triticum aestivum* L. | KU-1302 | Greece | 3.84 | -1.07 | - |
| 243 | *Triticum aestivum* L. | KU-1347 | Greece | 3.28 | -0.74 | - |
| 244 | *Triticum aestivum* L. | KU-1392 | Romania | 4.21 | -0.79 | - |
| 245 | *Triticum aestivum* L. | KU-1394 | Romania | 3.51 | -0.94 | - |
| 246 | *Triticum aestivum* L. | KU-1421 | Romania | 3.53 | -1.21 | - |
| 247 | *Triticum aestivum* L. | KU-1424 | Romania | 3.56 | -1.39 | - |
| 248 | *Triticum aestivum* L. | KU-1521 | The former Union of Soviet Socialist Republics | 4.02 | -1.06 | - |
| 249 | *Triticum aestivum* L. | KU-1527 | The former Union of Soviet Socialist Republics | 3.70 | -0.36 | - |
| 250 | *Triticum aestivum* L. | KU-1644 | The former Union of Soviet Socialist Republics | 3.67 | -1.12 | - |
| 251 | *Triticum aestivum* L. | KU-1668 | The former Union of Soviet Socialist Republics | 3.83 | -1.24 | - |
| 252 | *Triticum aestivum* L. | KU-1697 | The former Union of Soviet Socialist Republics | 4.12 | -1.07 | - |
| 253 | *Triticum aestivum* L. | KU-1797 | The former Union of Soviet Socialist Republics | 3.94 | -0.96 | - |
| 254 | *Triticum aestivum* L. | KU-1812 | Georgia | 3.68 | 0.26 | - |
| 255 | *Triticum aestivum* L. | KU-1814 | Georgia | 3.29 | -0.10 | - |
| 256 | *Triticum aestivum* L. | KU-1817 | Georgia | 3.52 | 0.23 | - |
| 257 | *Triticum aestivum* L. | KU-3004 | Pakistan | 3.59 | -0.11 | - |
| 258 | *Triticum aestivum* L. | KU-3006 | Pakistan | 3.58 | -0.36 | - |
| 259 | *Triticum aestivum* L. | KU-3010 | Pakistan | 3.45 | -0.36 | - |
| 260 | *Triticum aestivum* L. | KU-3037 | Pakistan | 3.63 | -0.62 | - |
| 261 | *Triticum aestivum* L. | KU-3045 | Afghanistan | 3.11 | -0.20 | - |
| 262 | *Triticum aestivum* L. | KU-3054 | Afghanistan | 2.70 | 0.02 | - |
| 263 | *Triticum aestivum* L. | KU-3062 | Afghanistan | 3.23 | -0.47 | - |
| 264 | *Triticum aestivum* L. | KU-3063 | Afganistan | 3.61 | -0.65 | - |
| 265 | *Triticum aestivum* L. | KU-3083 | Afghanistan | 3.29 | -1.11 | - |
| 266 | *Triticum aestivum* L. | KU-3089 | Afghanistan | 3.85 | 0.51 | - |
| 267 | *Triticum aestivum* L. | KU-3097 | Iran | 3.41 | -0.25 | - |
| 268 | *Triticum aestivum* L. | KU-3098 | Iran | 3.47 | -0.09 | - |
| 269 | *Triticum aestivum* L. | KU-3121 | Iran | 3.53 | -0.11 | - |
| 270 | *Triticum aestivum* L. | KU-3126 | Iran | 3.19 | 0.07 | - |
| 271 | *Triticum aestivum* L. | KU-3136 | Iran | 3.74 | -0.08 | - |
| 272 | *Triticum aestivum* L. | KU-3162 | Iran | 3.19 | -0.25 | - |
| 273 | *Triticum aestivum* L. | KU-3184 | Iran | 3.42 | -0.42 | - |
| 274 | *Triticum aestivum* L. | KU-3189 | Iran | 2.93 | 0.31 | - |
| 275 | *Triticum aestivum* L. | KU-3202 | Iran | 3.26 | -0.19 | - |
| 276 | *Triticum aestivum* L. | KU-3232 | Iran | 3.85 | 0.40 | - |
| 277 | *Triticum aestivum* L. | KU-3236 | Iran | 2.54 | -0.65 | - |
| 278 | *Triticum aestivum* L. | KU-3242 | Iran | 3.25 | -0.95 | - |
| 279 | *Triticum aestivum* L. | KU-3274 | Iran | 3.60 | -0.78 | - |
| 280 | *Triticum aestivum* L. | KU-3289 | Iran | 3.50 | -0.88 | - |
| 281 | *Triticum aestivum* L. | KU-3299 | Pakistan | 3.31 | -0.59 | - |
| 282 | *Triticum aestivum* L. | KU-3351 | Pakistan | 3.93 | -0.61 | - |
| 283 | *Triticum aestivum* L. | KU-3377 | Iran | 3.58 | -1.22 | - |
| 284 | *Triticum aestivum* L. | KU-3401 | The former German Democratic Republic | 4.18 | -0.38 | - |
| 285 | *Triticum aestivum* L. | KU-3413 | The former German Democratic Republic | 4.17 | -0.81 | - |
| 286 | *Triticum aestivum* L. | KU-3416 | The former German Democratic Republic | 3.70 | -1.04 | - |
| 287 | *Triticum aestivum* L. | KU-3417 | The former German Democratic Republic | 3.77 | -0.51 | - |
| 288 | *Triticum aestivum* L. | KU-3421 | The former German Democratic Republic | 4.00 | -0.71 | - |
| 289 | *Triticum aestivum* L. | KU-3443 | The former German Democratic Republic | 3.55 | -0.73 | - |
| 290 | *Triticum aestivum* L. | KU-3444 | The former German Democratic Republic | 3.62 | -0.87 | - |
| 291 | *Triticum aestivum* L. | KU-3445 | The former German Democratic Republic | 3.93 | -0.88 | - |
| 292 | *Triticum aestivum* L. | KU-3752 | Egypt | 4.10 | -0.26 | - |
| 293 | *Triticum aestivum* L. | KU-3777 | Jordan | 4.00 | -0.23 | - |
| 294 | *Triticum aestivum* L. | KU-3778 | Lebanon | 3.35 | -0.56 | - |
| 295 | *Triticum aestivum* L. | KU-3780 | Syria | 3.67 | -0.68 | - |
| 296 | *Triticum aestivum* L. | KU-3784 | Turkey | 3.23 | 0.06 | - |
| 297 | *Triticum aestivum* L. | KU-3789 | Turkey | 4.15 | -1.51 | - |
| 298 | *Triticum aestivum* L. | KU-3801 | Turkey | 3.26 | -0.02 | - |
| 299 | *Triticum aestivum* L. | KU-3806 | Turkey | 3.33 | 0.27 | - |
| 300 | *Triticum aestivum* L. | KU-3818 | Turkey | 3.37 | -0.82 | - |
| 301 | *Triticum aestivum* L. | KU-3834 | Turkey | 3.25 | -1.17 | - |
| 302 | *Triticum aestivum* L. | KU-3848 | Turkey | 3.44 | -0.60 | - |
| 303 | *Triticum aestivum* L. | KU-3851 | Turkey | 3.45 | -1.24 | - |
| 304 | *Triticum aestivum* L. | KU-3857 | Turkey | 3.16 | -1.11 | - |
| 305 | *Triticum aestivum* L. | KU-3860 | Turkey | 2.92 | -0.72 | - |
| 306 | *Triticum aestivum* L. | KU-3868 | Italy | 3.44 | -0.52 | - |
| 307 | *Triticum aestivum* L. | KU-4703 | Nepal | 2.70 | -1.01 | - |
| 308 | *Triticum aestivum* L. | KU-4714 | Nepal | 3.75 | 0.07 | - |
| 309 | *Triticum aestivum* L. | KU-4734 | Nepal | 3.82 | -0.41 | - |
| 310 | *Triticum aestivum* L. | KU-4759 | Nepal | 3.83 | -0.32 | - |
| 311 | *Triticum aestivum* L. | KU-4769 | Nepal | 3.48 | -0.34 | - |
| 312 | *Triticum aestivum* L. | KU-4783 | Nepal | 4.00 | -0.59 | - |
| 313 | *Triticum aestivum* L. | KU-7001 | Bhutan | 3.39 | -0.40 | - |
| 314 | *Triticum aestivum* L. | KU-7041 | Bhutan | 3.32 | -0.48 | - |
| 315 | *Triticum aestivum* L. | KU-7113 | Bhutan | 3.92 | -0.47 | - |
| 316 | *Triticum aestivum* L. | KU-7180 | Bhutan | 3.92 | -0.29 | - |
| 317 | *Triticum aestivum* L. | KU-7350 | Turkey | 3.72 | -0.54 | - |
| 318 | *Triticum aestivum* L. | KU-7356 | Ethiopia | 3.35 | -0.25 | - |
| 319 | *Triticum aestivum* L. | KU-7379 | Ethiopia | 3.36 | -0.99 | - |
| 320 | *Triticum aestivum* L. | KU-7406 | Ethiopia | 3.62 | -1.34 | - |
| 321 | *Triticum aestivum* L. | KU-7437 | Afghanistan | 3.68 | -0.22 | - |
| 322 | *Triticum aestivum* L. | KU-7459 | Afghanistan | 4.07 | -0.49 | - |
| 323 | *Triticum aestivum* L. | KU-7480 | Afghanistan | 2.95 | -0.48 | - |
| 324 | *Triticum aestivum* L. | KU-7624 | Afghanistan | 3.44 | -0.70 | - |
| 325 | *Triticum aestivum* L. | KU-7653 | Afghanistan | 3.42 | -0.74 | - |
| 326 | *Triticum aestivum* L. | KU-7669 | Afghanistan | 2.70 | -0.31 | - |
| 327 | *Triticum aestivum* L. | KU-9431 | Ethiopia | 3.65 | -0.12 | - |
| 328 | *Triticum aestivum* L. | KU-9460 | Ethiopia | 3.79 | 0.15 | - |
| 329 | *Triticum aestivum* L. | KU-9797 | Ethiopia | 3.80 | -0.19 | - |
| 330 | *Triticum aestivum* L. | KU-9820 | Ethiopia | 3.66 | -0.72 | - |
| 331 | *Triticum aestivum* L. | KU-9867 | Ethiopia | 4.44 | -0.41 | - |
| 332 | *Triticum aestivum* L. | KU-9873 | Ethiopia | -0.60 | -0.33 | - |
| 333 | *Triticum aestivum* L. | KU-10001 | Iraq | 3.27 | -1.28 | - |
| 334 | *Triticum aestivum* L. | KU-10154 | Iraq | 3.72 | -0.45 | - |
| 335 | *Triticum aestivum* L. | KU-10393 | Iran | 3.57 | -0.60 | - |
| 336 | *Triticum aestivum* L. | KU-10439 | Iran | 3.32 | -0.25 | - |
| 337 | *Triticum aestivum* L. | KU-10480 | Iran | 3.26 | -0.64 | - |
| 338 | *Triticum aestivum* L. | KU-10510 | Iran | 2.99 | -0.49 | - |
| 339 | *Triticum aestivum* L. | KU-11201 | Afghanistan | 3.93 | -0.30 | - |
| 340 | *Triticum aestivum* L. | KU-11214 | Afghanistan | 3.68 | 0.21 | - |
| 341 | *Triticum aestivum* L. | KU-11240A | Afghanistan | 3.64 | 0.05 | - |
| 342 | *Triticum aestivum* L. | KU-11351 | Romania | 3.58 | -0.07 | - |
| 343 | *Triticum aestivum* L. | KU-11702 | Greece | 3.90 | -1.46 | - |
| 344 | *Triticum aestivum* L. | KU-11809 | Greece | 3.89 | -1.01 | - |
| 345 | *Triticum aestivum* L. | KU-11829 | Greece | 3.44 | -0.50 | - |
| 346 | *Triticum aestivum* L. | KU-13501 | China | 3.66 | -1.00 | - |
| 347 | *Triticum aestivum* L. | KU-13506 | China | 3.29 | -0.13 | - |
| 348 | *Triticum aestivum* L. | KU-13546 | China | 3.79 | 0.24 | - |
| 349 | *Triticum aestivum* L. | KU-13631 | China | 4.11 | -0.34 | - |
| 350 | *Triticum aestivum* L. | KU-13662 | China | 3.83 | -0.36 | - |
| 351 | *Triticum aestivum* L. | KU-13708 | China | 3.80 | -0.20 | - |
| 352 | *Triticum aestivum* L. | KU-13807 | China | 3.92 | -0.22 | - |
| 353 | *Triticum aestivum* L. | KU-13891 | China | 3.89 | -0.07 | - |
| 354 | *Triticum aestivum* L. | Abukumawase (winter type) | Japan | 4.10 | -1.12 | - |
| 355 | *Triticum aestivum* L. | Akadaruma | Japan | 3.61 | -0.45 | - |
| 356 | *Triticum aestivum* L. | Ayahikari | Japan | 4.53 | -0.72 | - |
| 357 | *Triticum aestivum* L. | Bobwhite | Mexico | 4.01 | -1.03 | - |
| 358 | *Triticum aestivum* L. | Cheyenne | United States of America | 3.55 | -0.81 | - |
| 359 | *Triticum aestivum* L. | Chihokukomugi | Japan | 3.34 | -1.50 | - |
| 360 | *Triticum aestivum* L. | Chikugoizumi | Japan | 4.05 | -0.81 | - |
| 361 | *Triticum aestivum* L. | Chinese Spring | China | 3.86 | -0.49 | - |
| 362 | *Triticum aestivum* L. | Chogokuwase | Japan | 3.86 | -1.34 | - |
| 363 | *Triticum aestivum* L. | Fujimikomugi | Japan | 4.17 | -1.31 | - |
| 364 | *Triticum aestivum* L. | Gamenya | Autralia | 2.44 | -0.54 | - |
| 365 | *Triticum aestivum* L. | Hanamanten | Japan | 4.32 | -1.15 | - |
| 366 | *Triticum aestivum* L. | Haruyokoi | Japan | 3.59 | -0.63 | - |
| 367 | *Triticum aestivum* L. | Hokkai 240 | Japan | 3.92 | -0.63 | - |
| 368 | *Triticum aestivum* L. | Hope | United States of America | 3.45 | -0.96 | - |
| 369 | *Triticum aestivum* L. | Iwainodaichi | Japan | 3.92 | -1.26 | - |
| 370 | *Triticum aestivum* L. | Kanto 107 | Japan | 4.40 | -0.81 | - |
| 371 | *Triticum aestivum* L. | Kinuiroha | Japan | 4.23 | -0.70 | - |
| 372 | *Triticum aestivum* L. | Kitakamikomugi | Japan | 3.45 | -1.03 | - |
| 373 | *Triticum aestivum* L. | Kitanokaori | Japan | 3.77 | -0.93 | - |
| 374 | *Triticum aestivum* L. | KS831987 | United States of America | 4.19 | -1.28 | - |
| 375 | *Triticum aestivum* L. | Minaminokaori | Japan | 3.94 | -0.91 | - |
| 376 | *Triticum aestivum* L. | Minaminokomugi | Japan | 4.31 | -0.88 | - |
| 377 | *Triticum aestivum* L. | Nambukomugi | Japan | 3.86 | -0.43 | - |
| 378 | *Triticum aestivum* L. | Nebarigoshi | Japan | 4.07 | -1.19 | - |
| 379 | *Triticum aestivum* L. | Nishikazekomugi | Japan | 4.10 | -0.73 | - |
| 380 | *Triticum aestivum* L. | Nobeokabozykomugi | Japan | 3.66 | -0.61 | - |
| 381 | *Triticum aestivum* L. | Norin 26 | Japan | 4.42 | -0.99 | - |
| 382 | *Triticum aestivum* L. | Norin 61 | Japan | 4.36 | -0.67 | - |
| 383 | *Triticum aestivum* L. | Opata 85 | Mexico | 3.89 | -1.25 | - |
| 384 | *Triticum aestivum* L. | Saikai 165 | Japan | 4.40 | -0.98 | - |
| 385 | *Triticum aestivum* L. | Saikai 193 | Japan | 3.81 | -1.09 | - |
| 386 | *Triticum aestivum* L. | Shiroganekomugi | Japan | 4.05 | -1.06 | - |
| 387 | *Triticum aestivum* L. | Shyunyou | Japan | 4.06 | -1.00 | - |
| 388 | *Triticum aestivum* L. | Sumai #3 | China | 4.06 | -1.17 | - |
| 389 | *Triticum aestivum* L. | Synthetic W7984 | - | 0.92 | 1.65 | - |
| 390 | *Triticum aestivum* L. | Tamaizumi | Japan | 4.17 | -1.15 | - |
| 391 | *Triticum aestivum* L. | Timstein | United States of America | 3.65 | -0.96 | - |
| 392 | *Triticum aestivum* L. | U24 | China | 4.08 | -1.27 | - |
| 393 | *Triticum aestivum* L. | Variety duhamerianum | - | 4.45 | -0.85 | - |
| 394 | *Triticum aestivum* L. | Zenkojikomugi | Japan | 3.82 | -0.62 | - |
