# Supplementary material for: Genetic Basis for Spontaneous Hybrid Genome Doubling during Allopolyploid Speciation of Common Wheat Shown by Natural Variation Analyses of the Paternal Species
Source: PLoS One. 2013 Aug 8;8(8):e68310. doi: 10.1371/journal.pone.0068310 (PMC3738567; doi:10.1371/journal.pone.0068310)
Supplement: Table S3 — The STRUCTURE membership coefficients of the Ae . tauschii and T. aestivum accessions (K=2). The Ae . tauschii lineages are based on the PCA (Figure 1). A hyphen indicates that the information is not available. (DOCX) [file pone.0068310.s005.docx]

Table S3. The STRUCTURE membership coefficients of the *Ae. tauschii* and *T. aestivum* accessions (*K*=2).

| No. | Species | Accession | Origin | Lineage | Genepool1 | Genepool2 |
| --- | --- | --- | --- | --- | --- | --- |
| 1 | *Aegilops tauschii* Coss. | AE 1038 | Tajikistan | TauL1 | 0.998 | 0.002 |
| 2 | *Aegilops tauschii* Coss. | AE 1090 | Kazakhstan | TauL1 | 0.999 | 0.001 |
| 3 | *Aegilops tauschii* Coss. | AE 933 | Georgia | TauL1 | 0.998 | 0.002 |
| 4 | *Aegilops tauschii* Coss. | AT 47 | China | TauL1 | 0.998 | 0.002 |
| 5 | *Aegilops tauschii* Coss. | AT 55 | China | TauL1 | 0.9973 | 0.0027 |
| 6 | *Aegilops tauschii* Coss. | AT 60 | China | TauL1 | 0.997 | 0.003 |
| 7 | *Aegilops tauschii* Coss. | AT 76 | China | TauL1 | 0.9975 | 0.0025 |
| 8 | *Aegilops tauschii* Coss. | AT 80 | China | TauL1 | 0.997 | 0.003 |
| 9 | *Aegilops tauschii* Coss. | CGN 10734 | Armenia | TauL1 | 0.999 | 0.001 |
| 10 | *Aegilops tauschii* Coss. | CGN 10767 | Pakistan | TauL1 | 0.999 | 0.001 |
| 11 | *Aegilops tauschii* Coss. | CGN 10768 | Pakistan | TauL1 | 0.999 | 0.001 |
| 12 | *Aegilops tauschii* Coss. | CGN 10769 | Pakistan | TauL1 | 0.999 | 0.001 |
| 13 | *Aegilops tauschii* Coss. | CGN 10770 | Pakistan | TauL1 | 0.999 | 0.001 |
| 14 | *Aegilops tauschii* Coss. | CGN 10771 | Pakistan | TauL1 | 0.999 | 0.001 |
| 15 | *Aegilops tauschii* Coss. | IG 108561 | Pakistan | TauL1 | 0.999 | 0.001 |
| 16 | *Aegilops tauschii* Coss. | IG 120735 | Turkmenistan | TauL1 | 0.992 | 0.008 |
| 17 | *Aegilops tauschii* Coss. | IG 120736 | Uzbekistan | TauL1 | 0.999 | 0.001 |
| 18 | *Aegilops tauschii* Coss. | IG 123910 | Uzbekistan | TauL1 | 0.999 | 0.001 |
| 19 | *Aegilops tauschii* Coss. | IG 126273 | Armenia | TauL1 | 0.9945 | 0.0055 |
| 20 | *Aegilops tauschii* Coss. | IG 126280 | Armenia | TauL1 | 0.998 | 0.002 |
| 21 | *Aegilops tauschii* Coss. | IG 126293 | Armenia | TauL1 | 0.9981 | 0.0019 |
| 22 | *Aegilops tauschii* Coss. | IG 126353 | Armenia | TauL1 | 0.999 | 0.001 |
| 23 | *Aegilops tauschii* Coss. | IG 126387 | Turkmenistan | TauL1 | 0.998 | 0.002 |
| 24 | *Aegilops tauschii* Coss. | IG 126489 | Turkmenistan | TauL1 | 0.9984 | 0.0016 |
| 25 | *Aegilops tauschii* Coss. | IG 127015 | Armenia | TauL1 | 0.999 | 0.001 |
| 26 | *Aegilops tauschii* Coss. | IG 131606 | Kyrgyzstan | TauL1 | 0.999 | 0.001 |
| 27 | *Aegilops tauschii* Coss. | IG 46663 | Pakistan | TauL1 | 0.999 | 0.001 |
| 28 | *Aegilops tauschii* Coss. | IG 46666 | Pakistan | TauL1 | 0.999 | 0.001 |
| 29 | *Aegilops tauschii* Coss. | IG 46682 | Pakistan | TauL1 | 0.999 | 0.001 |
| 30 | *Aegilops tauschii* Coss. | IG 47196 | Azerbaijan | TauL1 | 0.998 | 0.002 |
| 31 | *Aegilops tauschii* Coss. | IG 47259 | Syria | TauL1 | 0.999 | 0.001 |
| 32 | *Aegilops tauschii* Coss. | IG 48042 | India | TauL1 | 0.998 | 0.002 |
| 33 | *Aegilops tauschii* Coss. | IG 48508 | Turkmenistan | TauL1 | 0.9918 | 0.0082 |
| 34 | *Aegilops tauschii* Coss. | IG 48518 | Turkmenistan | TauL1 | 0.998 | 0.002 |
| 35 | *Aegilops tauschii* Coss. | IG 48539 | Uzbekistan | TauL1 | 0.9981 | 0.0019 |
| 36 | *Aegilops tauschii* Coss. | IG 48554 | Tajikistan | TauL1 | 0.999 | 0.001 |
| 37 | *Aegilops tauschii* Coss. | IG 48559 | Tajikistan | TauL1 | 0.999 | 0.001 |
| 38 | *Aegilops tauschii* Coss. | IG 48564 | Tajikistan | TauL1 | 0.9989 | 0.0011 |
| 39 | *Aegilops tauschii* Coss. | IG 48565 | Uzbekistan | TauL1 | 0.999 | 0.001 |
| 40 | *Aegilops tauschii* Coss. | IG 48567 | Uzbekistan | TauL1 | 0.999 | 0.001 |
| 41 | *Aegilops tauschii* Coss. | IG 48747 | Armenia | TauL1 | 0.999 | 0.001 |
| 42 | *Aegilops tauschii* Coss. | IG 48748 | Armenia | TauL1 | 0.999 | 0.001 |
| 43 | *Aegilops tauschii* Coss. | IG 48758 | Armenia | TauL1 | 0.999 | 0.001 |
| 44 | *Aegilops tauschii* Coss. | IG 49095 | Iran | TauL1 | 0.999 | 0.001 |
| 45 | *Aegilops tauschii* Coss. | KU-20-6 | Pakistan | TauL1 | 0.999 | 0.001 |
| 46 | *Aegilops tauschii* Coss. | KU-2001 | Pakistan | TauL1 | 0.999 | 0.001 |
| 47 | *Aegilops tauschii* Coss. | KU-2003 | Pakistan | TauL1 | 0.999 | 0.001 |
| 48 | *Aegilops tauschii* Coss. | KU-2006 | Pakistan | TauL1 | 0.999 | 0.001 |
| 49 | *Aegilops tauschii* Coss. | KU-2008 | Pakistan | TauL1 | 0.999 | 0.001 |
| 50 | *Aegilops tauschii* Coss. | KU-2010 | Afghanistan | TauL1 | 0.999 | 0.001 |
| 51 | *Aegilops tauschii* Coss. | KU-2012 | Afghanistan | TauL1 | 0.9966 | 0.0034 |
| 52 | *Aegilops tauschii* Coss. | KU-2016 | Afghanistan | TauL1 | 0.999 | 0.001 |
| 53 | *Aegilops tauschii* Coss. | KU-2018 | Afghanistan | TauL1 | 0.999 | 0.001 |
| 54 | *Aegilops tauschii* Coss. | KU-2022 | Afghanistan | TauL1 | 0.999 | 0.001 |
| 55 | *Aegilops tauschii* Coss. | KU-2025 | Afghanistan | TauL1 | 0.999 | 0.001 |
| 56 | *Aegilops tauschii* Coss. | KU-2027 | Afghanistan | TauL1 | 0.995 | 0.005 |
| 57 | *Aegilops tauschii* Coss. | KU-2028 | Afghanistan | TauL1 | 0.999 | 0.001 |
| 58 | *Aegilops tauschii* Coss. | KU-2032 | Afghanistan | TauL1 | 0.9959 | 0.0041 |
| 59 | *Aegilops tauschii* Coss. | KU-2035 | Afghanistan | TauL1 | 0.999 | 0.001 |
| 60 | *Aegilops tauschii* Coss. | KU-2039 | Afghanistan | TauL1 | 0.9987 | 0.0013 |
| 61 | *Aegilops tauschii* Coss. | KU-2042 | Afghanistan | TauL1 | 0.999 | 0.001 |
| 62 | *Aegilops tauschii* Coss. | KU-2043 | Afghanistan | TauL1 | 0.999 | 0.001 |
| 63 | *Aegilops tauschii* Coss. | KU-2044 | Afghanistan | TauL1 | 0.9987 | 0.0013 |
| 64 | *Aegilops tauschii* Coss. | KU-2050 | Afghanistan | TauL1 | 0.999 | 0.001 |
| 65 | *Aegilops tauschii* Coss. | KU-2051 | Afghanistan | TauL1 | 0.999 | 0.001 |
| 66 | *Aegilops tauschii* Coss. | KU-2056 | Afghanistan | TauL1 | 0.999 | 0.001 |
| 67 | *Aegilops tauschii* Coss. | KU-2058 | Afghanistan | TauL1 | 0.999 | 0.001 |
| 68 | *Aegilops tauschii* Coss. | KU-2059 | Afghanistan | TauL1 | 0.999 | 0.001 |
| 69 | *Aegilops tauschii* Coss. | KU-2061 | Afghanistan | TauL1 | 0.999 | 0.001 |
| 70 | *Aegilops tauschii* Coss. | KU-2063 | Afghanistan | TauL1 | 0.999 | 0.001 |
| 71 | *Aegilops tauschii* Coss. | KU-2066 | Afghanistan | TauL1 | 0.998 | 0.002 |
| 72 | *Aegilops tauschii* Coss. | KU-2068 | Iran | TauL1 | 0.999 | 0.001 |
| 73 | *Aegilops tauschii* Coss. | KU-2082 | Iran | TauL1 | 0.9947 | 0.0053 |
| 74 | *Aegilops tauschii* Coss. | KU-2087 | Iran | TauL1 | 0.9879 | 0.0121 |
| 75 | *Aegilops tauschii* Coss. | KU-2113 | Iran | TauL1 | 0.9989 | 0.0011 |
| 76 | *Aegilops tauschii* Coss. | KU-2115 | Iran | TauL1 | 0.9849 | 0.0151 |
| 77 | *Aegilops tauschii* Coss. | KU-2116 | Iran | TauL1 | 0.999 | 0.001 |
| 78 | *Aegilops tauschii* Coss. | KU-2120 | Iran | TauL1 | 0.9985 | 0.0015 |
| 79 | *Aegilops tauschii* Coss. | KU-2121 | Iran | TauL1 | 0.9971 | 0.0029 |
| 80 | *Aegilops tauschii* Coss. | KU-2122 | Iran | TauL1 | 0.973 | 0.027 |
| 81 | *Aegilops tauschii* Coss. | KU-2131 | Turkey | TauL1 | 0.9968 | 0.0032 |
| 82 | *Aegilops tauschii* Coss. | KU-2132 | Turkey | TauL1 | 0.9965 | 0.0035 |
| 83 | *Aegilops tauschii* Coss. | KU-2133 | Turkey | TauL1 | 0.9983 | 0.0017 |
| 84 | *Aegilops tauschii* Coss. | KU-2136 | Turkey | TauL1 | 0.9961 | 0.0039 |
| 85 | *Aegilops tauschii* Coss. | KU-2137 | Turkey | TauL1 | 0.9894 | 0.0106 |
| 86 | *Aegilops tauschii* Coss. | KU-2138 | Turkey | TauL1 | 0.9984 | 0.0016 |
| 87 | *Aegilops tauschii* Coss. | KU-2140 | Turkey | TauL1 | 0.9985 | 0.0015 |
| 88 | *Aegilops tauschii* Coss. | KU-2141 | Turkey | TauL1 | 0.9987 | 0.0013 |
| 89 | *Aegilops tauschii* Coss. | KU-2142 | Iran | TauL1 | 0.998 | 0.002 |
| 90 | *Aegilops tauschii* Coss. | KU-2143 | Iran | TauL1 | 0.9978 | 0.0022 |
| 91 | *Aegilops tauschii* Coss. | KU-2144 | Iran | TauL1 | 0.995 | 0.005 |
| 92 | *Aegilops tauschii* Coss. | KU-2145 | Iran | TauL1 | 0.9989 | 0.0011 |
| 93 | *Aegilops tauschii* Coss. | KU-2148 | Iran | TauL1 | 0.999 | 0.001 |
| 94 | *Aegilops tauschii* Coss. | KU-2149 | Iran | TauL1 | 0.998 | 0.002 |
| 95 | *Aegilops tauschii* Coss. | KU-2150 | Iran | TauL1 | 0.9988 | 0.0012 |
| 96 | *Aegilops tauschii* Coss. | KU-2151 | Iran | TauL1 | 0.9941 | 0.0059 |
| 97 | *Aegilops tauschii* Coss. | KU-2152 | Iran | TauL1 | 0.999 | 0.001 |
| 98 | *Aegilops tauschii* Coss. | KU-2153 | Iran | TauL1 | 0.999 | 0.001 |
| 99 | *Aegilops tauschii* Coss. | KU-2154 | Iran | TauL1 | 0.997 | 0.003 |
| 100 | *Aegilops tauschii* Coss. | KU-2157 | Iran | TauL1 | 0.998 | 0.002 |
| 101 | *Aegilops tauschii* Coss. | KU-2612 | Afghanistan | TauL1 | 0.999 | 0.001 |
| 102 | *Aegilops tauschii* Coss. | KU-2617 | Afghanistan | TauL1 | 0.999 | 0.001 |
| 103 | *Aegilops tauschii* Coss. | KU-2619 | Afghanistan | TauL1 | 0.9984 | 0.0016 |
| 104 | *Aegilops tauschii* Coss. | KU-2621 | Afghanistan | TauL1 | 0.999 | 0.001 |
| 105 | *Aegilops tauschii* Coss. | KU-2624 | Afghanistan | TauL1 | 0.999 | 0.001 |
| 106 | *Aegilops tauschii* Coss. | KU-2627 | Afghanistan | TauL1 | 0.999 | 0.001 |
| 107 | *Aegilops tauschii* Coss. | KU-2630 | Afghanistan | TauL1 | 0.999 | 0.001 |
| 108 | *Aegilops tauschii* Coss. | KU-2632 | Afghanistan | TauL1 | 0.999 | 0.001 |
| 109 | *Aegilops tauschii* Coss. | KU-2633 | Afghanistan | TauL1 | 0.999 | 0.001 |
| 110 | *Aegilops tauschii* Coss. | KU-2635 | Afghanistan | TauL1 | 0.999 | 0.001 |
| 111 | *Aegilops tauschii* Coss. | KU-2636 | Afghanistan | TauL1 | 0.999 | 0.001 |
| 112 | *Aegilops tauschii* Coss. | KU-2638 | Afghanistan | TauL1 | 0.999 | 0.001 |
| 113 | *Aegilops tauschii* Coss. | KU-2639 | Afghanistan | TauL1 | 0.998 | 0.002 |
| 114 | *Aegilops tauschii* Coss. | KU-2809 | Armenia | TauL1 | 0.998 | 0.002 |
| 115 | *Aegilops tauschii* Coss. | KU-2810 | Armenia | TauL1 | 0.998 | 0.002 |
| 116 | *Aegilops tauschii* Coss. | KU-2814 | Armenia | TauL1 | 0.998 | 0.002 |
| 117 | *Aegilops tauschii* Coss. | KU-2816 | Armenia | TauL1 | 0.9937 | 0.0063 |
| 118 | *Aegilops tauschii* Coss. | KU-2821 | Armenia | TauL1 | 0.9949 | 0.0051 |
| 119 | *Aegilops tauschii* Coss. | KU-2822A | Armenia | TauL1 | 0.996 | 0.004 |
| 120 | *Aegilops tauschii* Coss. | KU-2823 | Armenia | TauL1 | 0.999 | 0.001 |
| 121 | *Aegilops tauschii* Coss. | KU-2824 | Armenia | TauL1 | 0.998 | 0.002 |
| 122 | *Aegilops tauschii* Coss. | KU-2826 | Georgia | TauL1 | 0.997 | 0.003 |
| 123 | *Aegilops tauschii* Coss. | KU-2828 | Georgia | TauL1 | 0.9622 | 0.0378 |
| 124 | *Aegilops tauschii* Coss. | KU-2834 | Georgia | TauL1 | 0.9915 | 0.0085 |
| 125 | *Aegilops tauschii* Coss. | KU-2836 | Georgia | TauL1 | 0.9867 | 0.0133 |
| 126 | *Aegilops tauschii* Coss. | PI 476874 | Afghanistan | TauL1 | 0.9985 | 0.0015 |
| 127 | *Aegilops tauschii* Coss. | PI 486270 | Turkey | TauL1 | 0.999 | 0.001 |
| 128 | *Aegilops tauschii* Coss. | PI 486274 | Turkey | TauL1 | 0.9985 | 0.0015 |
| 129 | *Aegilops tauschii* Coss. | PI 486277 | Turkey | TauL1 | 0.998 | 0.002 |
| 130 | *Aegilops tauschii* Coss. | PI 499262 | China | TauL1 | 0.999 | 0.001 |
| 131 | *Aegilops tauschii* Coss. | PI 508262 | China | TauL1 | 0.999 | 0.001 |
| 132 | *Aegilops tauschii* Coss. | PI 508264 | China | TauL1 | 0.997 | 0.003 |
| 133 | *Aegilops tauschii* Coss. | PI 554319 | Turkey | TauL1 | 0.9981 | 0.0019 |
| 134 | *Aegilops tauschii* Coss. | AE 1037 | Georgia | TauL2 | 0.5123 | 0.4877 |
| 135 | *Aegilops tauschii* Coss. | IG 120863 | Dagestan | TauL2 | 0.5084 | 0.4916 |
| 136 | *Aegilops tauschii* Coss. | IG 120866 | Dagestan | TauL2 | 0.4473 | 0.5527 |
| 137 | *Aegilops tauschii* Coss. | IG 126991 | Armenia | TauL2 | 0.5583 | 0.4417 |
| 138 | *Aegilops tauschii* Coss. | IG 46623 | Syria | TauL2 | 0.5245 | 0.4755 |
| 139 | *Aegilops tauschii* Coss. | IG 47173 | Armenia | TauL2 | 0.4289 | 0.5711 |
| 140 | *Aegilops tauschii* Coss. | IG 47182 | Azerbaijan | TauL2 | 0.4598 | 0.5402 |
| 141 | *Aegilops tauschii* Coss. | IG 47186 | Azerbaijan | TauL2 | 0.5251 | 0.4749 |
| 142 | *Aegilops tauschii* Coss. | IG 47188 | Azerbaijan | TauL2 | 0.4053 | 0.5947 |
| 143 | *Aegilops tauschii* Coss. | IG 47192 | Azerbaijan | TauL2 | 0.4395 | 0.5605 |
| 144 | *Aegilops tauschii* Coss. | IG 47193 | Azerbaijan | TauL2 | 0.4998 | 0.5002 |
| 145 | *Aegilops tauschii* Coss. | IG 47194 | Azerbaijan | TauL2 | 0.5172 | 0.4828 |
| 146 | *Aegilops tauschii* Coss. | IG 47199 | Azerbaijan | TauL2 | 0.5494 | 0.4506 |
| 147 | *Aegilops tauschii* Coss. | IG 47202 | Azerbaijan | TauL2 | 0.4725 | 0.5275 |
| 148 | *Aegilops tauschii* Coss. | IG 47203 | Azerbaijan | TauL2 | 0.5813 | 0.4187 |
| 149 | *Aegilops tauschii* Coss. | IG 47204 | Azerbaijan | TauL2 | 0.6033 | 0.3967 |
| 150 | *Aegilops tauschii* Coss. | IG 48274 | Dagestan | TauL2 | 0.5432 | 0.4568 |
| 151 | *Aegilops tauschii* Coss. | KU-20-1 | Dagestan | TauL2 | 0.5516 | 0.4484 |
| 152 | *Aegilops tauschii* Coss. | KU-20-10 | Iran | TauL2 | 0.3642 | 0.6358 |
| 153 | *Aegilops tauschii* Coss. | KU-20-7 | Iran | TauL2 | 0.5595 | 0.4405 |
| 154 | *Aegilops tauschii* Coss. | KU-20-8 | Iran | TauL2 | 0.5542 | 0.4458 |
| 155 | *Aegilops tauschii* Coss. | KU-20-9 | Iran | TauL2 | 0.5363 | 0.4637 |
| 156 | *Aegilops tauschii* Coss. | KU-2069 | Iran | TauL2 | 0.5191 | 0.4809 |
| 157 | *Aegilops tauschii* Coss. | KU-2074 | Iran | TauL2 | 0.5272 | 0.4728 |
| 158 | *Aegilops tauschii* Coss. | KU-2075 | Iran | TauL2 | 0.5298 | 0.4702 |
| 159 | *Aegilops tauschii* Coss. | KU-2076 | Iran | TauL2 | 0.4988 | 0.5012 |
| 160 | *Aegilops tauschii* Coss. | KU-2077 | Iran | TauL2 | 0.5481 | 0.4519 |
| 161 | *Aegilops tauschii* Coss. | KU-2078 | Iran | TauL2 | 0.4871 | 0.5129 |
| 162 | *Aegilops tauschii* Coss. | KU-2079 | Iran | TauL2 | 0.5696 | 0.4304 |
| 163 | *Aegilops tauschii* Coss. | KU-2080 | Iran | TauL2 | 0.5713 | 0.4287 |
| 164 | *Aegilops tauschii* Coss. | KU-2083 | Iran | TauL2 | 0.5151 | 0.4849 |
| 165 | *Aegilops tauschii* Coss. | KU-2086 | Iran | TauL2 | 0.5267 | 0.4733 |
| 166 | *Aegilops tauschii* Coss. | KU-2088 | Iran | TauL2 | 0.5982 | 0.4018 |
| 167 | *Aegilops tauschii* Coss. | KU-2090 | Iran | TauL2 | 0.6144 | 0.3856 |
| 168 | *Aegilops tauschii* Coss. | KU-2091 | Iran | TauL2 | 0.5302 | 0.4698 |
| 169 | *Aegilops tauschii* Coss. | KU-2092 | Iran | TauL2 | 0.4849 | 0.5151 |
| 170 | *Aegilops tauschii* Coss. | KU-2093 | Iran | TauL2 | 0.54 | 0.46 |
| 171 | *Aegilops tauschii* Coss. | KU-2096 | Iran | TauL2 | 0.5639 | 0.4361 |
| 172 | *Aegilops tauschii* Coss. | KU-2097 | Iran | TauL2 | 0.5398 | 0.4602 |
| 173 | *Aegilops tauschii* Coss. | KU-2098 | Iran | TauL2 | 0.5437 | 0.4563 |
| 174 | *Aegilops tauschii* Coss. | KU-2100 | Iran | TauL2 | 0.424 | 0.576 |
| 175 | *Aegilops tauschii* Coss. | KU-2101 | Iran | TauL2 | 0.5375 | 0.4625 |
| 176 | *Aegilops tauschii* Coss. | KU-2102 | Iran | TauL2 | 0.48 | 0.52 |
| 177 | *Aegilops tauschii* Coss. | KU-2103 | Iran | TauL2 | 0.5061 | 0.4939 |
| 178 | *Aegilops tauschii* Coss. | KU-2104 | Iran | TauL2 | 0.4029 | 0.5971 |
| 179 | *Aegilops tauschii* Coss. | KU-2105 | Iran | TauL2 | 0.421 | 0.579 |
| 180 | *Aegilops tauschii* Coss. | KU-2106 | Iran | TauL2 | 0.4397 | 0.5603 |
| 181 | *Aegilops tauschii* Coss. | KU-2107 | Iran | TauL2 | 0.4753 | 0.5247 |
| 182 | *Aegilops tauschii* Coss. | KU-2108 | Iran | TauL2 | 0.4846 | 0.5154 |
| 183 | *Aegilops tauschii* Coss. | KU-2109 | Iran | TauL2 | 0.4399 | 0.5601 |
| 184 | *Aegilops tauschii* Coss. | KU-2110 | Iran | TauL2 | 0.4654 | 0.5346 |
| 185 | *Aegilops tauschii* Coss. | KU-2111 | Iran | TauL2 | 0.583 | 0.417 |
| 186 | *Aegilops tauschii* Coss. | KU-2112 | Iran | TauL2 | 0.5484 | 0.4516 |
| 187 | *Aegilops tauschii* Coss. | KU-2118 | Iran | TauL2 | 0.4582 | 0.5418 |
| 188 | *Aegilops tauschii* Coss. | KU-2124 | Iran | TauL2 | 0.5073 | 0.4927 |
| 189 | *Aegilops tauschii* Coss. | KU-2126 | Iran | TauL2 | 0.5073 | 0.4927 |
| 190 | *Aegilops tauschii* Coss. | KU-2155 | Iran | TauL2 | 0.4821 | 0.5179 |
| 191 | *Aegilops tauschii* Coss. | KU-2156 | Iran | TauL2 | 0.465 | 0.535 |
| 192 | *Aegilops tauschii* Coss. | KU-2158 | Iran | TauL2 | 0.5119 | 0.4881 |
| 193 | *Aegilops tauschii* Coss. | KU-2159 | Iran | TauL2 | 0.493 | 0.507 |
| 194 | *Aegilops tauschii* Coss. | KU-2160 | Iran | TauL2 | 0.4792 | 0.5208 |
| 195 | *Aegilops tauschii* Coss. | KU-2801 | Azerbaijan | TauL2 | 0.5146 | 0.4854 |
| 196 | *Aegilops tauschii* Coss. | KU-2804 | Azerbaijan | TauL2 | 0.5481 | 0.4519 |
| 197 | *Aegilops tauschii* Coss. | KU-2806 | Azerbaijan | TauL2 | 0.4879 | 0.5121 |
| 198 | *Aegilops tauschii* Coss. | KU-2811 | Armenia | TauL2 | 0.4907 | 0.5093 |
| 199 | *Aegilops tauschii* Coss. | KU-2827 | Georgia | TauL2 | 0.5141 | 0.4859 |
| 200 | *Aegilops tauschii* Coss. | KU-2835B | Georgia | TauL2 | 0.5337 | 0.4663 |
| 201 | *Aegilops tauschii* Coss. | PI 486267 | Turkey | TauL2 | 0.5316 | 0.4684 |
| 202 | *Aegilops tauschii* Coss. | AE 454 | Georgia | TauL3 | 0.5654 | 0.4346 |
| 203 | *Aegilops tauschii* Coss. | AE 457 | Georgia | TauL3 | 0.5711 | 0.4289 |
| 204 | *Aegilops tauschii* Coss. | AE 929 | Georgia | TauL3 | 0.6231 | 0.3769 |
| 205 | *Aegilops tauschii* Coss. | KU-2829A | Georgia | TauL3 | 0.5888 | 0.4112 |
| 206 | *Aegilops tauschii* Coss. | KU-2832 | Georgia | TauL3 | 0.6004 | 0.3996 |
| 207 | *Triticum aestivum* L. | KU-152 | China | - | 0.002 | 0.998 |
| 208 | *Triticum aestivum* L. | KU-161 | - | - | 0.0258 | 0.9742 |
| 209 | *Triticum aestivum* L. | KU-162-2 | Pakistan | - | 0.002 | 0.998 |
| 210 | *Triticum aestivum* L. | KU-166 | China | - | 0.001 | 0.999 |
| 211 | *Triticum aestivum* L. | KU-192 | - | - | 0.0058 | 0.9942 |
| 212 | *Triticum aestivum* L. | KU-197 | Turkey | - | 0.003 | 0.997 |
| 213 | *Triticum aestivum* L. | KU-265 | Japan | - | 0.002 | 0.998 |
| 214 | *Triticum aestivum* L. | KU-309 | United States of America | - | 0.009 | 0.991 |
| 215 | *Triticum aestivum* L. | KU-333 | Canada | - | 0.026 | 0.974 |
| 216 | *Triticum aestivum* L. | KU-336 | United States of America | - | 0.0039 | 0.9961 |
| 217 | *Triticum aestivum* L. | KU-366 | United Kingdom | - | 0.002 | 0.998 |
| 218 | *Triticum aestivum* L. | KU-370 | United Kingdom | - | 0.002 | 0.998 |
| 219 | *Triticum aestivum* L. | KU-371 | United Kingdom | - | 0.0017 | 0.9983 |
| 220 | *Triticum aestivum* L. | KU-372 | United Kingdom | - | 0.002 | 0.998 |
| 221 | *Triticum aestivum* L. | KU-373 | United Kingdom | - | 0.0855 | 0.9145 |
| 222 | *Triticum aestivum* L. | KU-374 | United Kingdom | - | 0.003 | 0.997 |
| 223 | *Triticum aestivum* L. | KU-405 | The former Union of Soviet Socialist Republics | - | 0.001 | 0.999 |
| 224 | *Triticum aestivum* L. | KU-479 | China | - | 0.0025 | 0.9975 |
| 225 | *Triticum aestivum* L. | KU-481 | China | - | 0.002 | 0.998 |
| 226 | *Triticum aestivum* L. | KU-483 | Tanzania | - | 0.0012 | 0.9988 |
| 227 | *Triticum aestivum* L. | KU-497 | India | - | 0.0233 | 0.9767 |
| 228 | *Triticum aestivum* L. | KU-504 | China | - | 0.001 | 0.999 |
| 229 | *Triticum aestivum* L. | KU-601 | Japan | - | 0.001 | 0.999 |
| 230 | *Triticum aestivum* L. | KU-1002 | Spain | - | 0.0763 | 0.9237 |
| 231 | *Triticum aestivum* L. | KU-1005 | Spain | - | 0.002 | 0.998 |
| 232 | *Triticum aestivum* L. | KU-1011 | Spain | - | 0.001 | 0.999 |
| 233 | *Triticum aestivum* L. | KU-1020 | Spain | - | 0.001 | 0.999 |
| 234 | *Triticum aestivum* L. | KU-1049 | Spain | - | 0.002 | 0.998 |
| 235 | *Triticum aestivum* L. | KU-1062 | Spain | - | 0.0011 | 0.9989 |
| 236 | *Triticum aestivum* L. | KU-1137 | Spain | - | 0.006 | 0.994 |
| 237 | *Triticum aestivum* L. | KU-1143 | Spain | - | 0.001 | 0.999 |
| 238 | *Triticum aestivum* L. | KU-1208 | Japan | - | 0.0101 | 0.9899 |
| 239 | *Triticum aestivum* L. | KU-1215 | Japan | - | 0.0011 | 0.9989 |
| 240 | *Triticum aestivum* L. | KU-1230 | Japan | - | 0.001 | 0.999 |
| 241 | *Triticum aestivum* L. | KU-1279 | Japan | - | 0.002 | 0.998 |
| 242 | *Triticum aestivum* L. | KU-1302 | Greece | - | 0.002 | 0.998 |
| 243 | *Triticum aestivum* L. | KU-1347 | Greece | - | 0.0074 | 0.9926 |
| 244 | *Triticum aestivum* L. | KU-1392 | Romania | - | 0.001 | 0.999 |
| 245 | *Triticum aestivum* L. | KU-1394 | Romania | - | 0.002 | 0.998 |
| 246 | *Triticum aestivum* L. | KU-1421 | Romania | - | 0.0186 | 0.9814 |
| 247 | *Triticum aestivum* L. | KU-1424 | Romania | - | 0.0048 | 0.9952 |
| 248 | *Triticum aestivum* L. | KU-1521 | The former Union of Soviet Socialist Republics | - | 0.0018 | 0.9982 |
| 249 | *Triticum aestivum* L. | KU-1527 | The former Union of Soviet Socialist Republics | - | 0.0131 | 0.9869 |
| 250 | *Triticum aestivum* L. | KU-1644 | The former Union of Soviet Socialist Republics | - | 0.002 | 0.998 |
| 251 | *Triticum aestivum* L. | KU-1668 | The former Union of Soviet Socialist Republics | - | 0.002 | 0.998 |
| 252 | *Triticum aestivum* L. | KU-1697 | The former Union of Soviet Socialist Republics | - | 0.002 | 0.998 |
| 253 | *Triticum aestivum* L. | KU-1797 | The former Union of Soviet Socialist Republics | - | 0.0026 | 0.9974 |
| 254 | *Triticum aestivum* L. | KU-1812 | Georgia | - | 0.0062 | 0.9938 |
| 255 | *Triticum aestivum* L. | KU-1814 | Georgia | - | 0.0335 | 0.9665 |
| 256 | *Triticum aestivum* L. | KU-1817 | Georgia | - | 0.003 | 0.997 |
| 257 | *Triticum aestivum* L. | KU-3004 | Pakistan | - | 0.002 | 0.998 |
| 258 | *Triticum aestivum* L. | KU-3006 | Pakistan | - | 0.002 | 0.998 |
| 259 | *Triticum aestivum* L. | KU-3010 | Pakistan | - | 0.0241 | 0.9759 |
| 260 | *Triticum aestivum* L. | KU-3037 | Pakistan | - | 0.0056 | 0.9944 |
| 261 | *Triticum aestivum* L. | KU-3045 | Afghanistan | - | 0.0603 | 0.9397 |
| 262 | *Triticum aestivum* L. | KU-3054 | Afghanistan | - | 0.0567 | 0.9433 |
| 263 | *Triticum aestivum* L. | KU-3062 | Afghanistan | - | 0.0146 | 0.9854 |
| 264 | *Triticum aestivum* L. | KU-3063 | Afganistan | - | 0.002 | 0.998 |
| 265 | *Triticum aestivum* L. | KU-3083 | Afghanistan | - | 0.0155 | 0.9845 |
| 266 | *Triticum aestivum* L. | KU-3089 | Afghanistan | - | 0.0023 | 0.9977 |
| 267 | *Triticum aestivum* L. | KU-3097 | Iran | - | 0.002 | 0.998 |
| 268 | *Triticum aestivum* L. | KU-3098 | Iran | - | 0.002 | 0.998 |
| 269 | *Triticum aestivum* L. | KU-3121 | Iran | - | 0.0019 | 0.9981 |
| 270 | *Triticum aestivum* L. | KU-3126 | Iran | - | 0.0021 | 0.9979 |
| 271 | *Triticum aestivum* L. | KU-3136 | Iran | - | 0.0043 | 0.9957 |
| 272 | *Triticum aestivum* L. | KU-3162 | Iran | - | 0.0173 | 0.9827 |
| 273 | *Triticum aestivum* L. | KU-3184 | Iran | - | 0.0368 | 0.9632 |
| 274 | *Triticum aestivum* L. | KU-3189 | Iran | - | 0.0826 | 0.9174 |
| 275 | *Triticum aestivum* L. | KU-3202 | Iran | - | 0.0125 | 0.9875 |
| 276 | *Triticum aestivum* L. | KU-3232 | Iran | - | 0.0232 | 0.9768 |
| 277 | *Triticum aestivum* L. | KU-3236 | Iran | - | 0.1342 | 0.8658 |
| 278 | *Triticum aestivum* L. | KU-3242 | Iran | - | 0.0028 | 0.9972 |
| 279 | *Triticum aestivum* L. | KU-3274 | Iran | - | 0.0021 | 0.9979 |
| 280 | *Triticum aestivum* L. | KU-3289 | Iran | - | 0.0133 | 0.9867 |
| 281 | *Triticum aestivum* L. | KU-3299 | Pakistan | - | 0.0141 | 0.9859 |
| 282 | *Triticum aestivum* L. | KU-3351 | Pakistan | - | 0.001 | 0.999 |
| 283 | *Triticum aestivum* L. | KU-3377 | Iran | - | 0.0062 | 0.9938 |
| 284 | *Triticum aestivum* L. | KU-3401 | The former German Democratic Republic | - | 0.002 | 0.998 |
| 285 | *Triticum aestivum* L. | KU-3413 | The former German Democratic Republic | - | 0.001 | 0.999 |
| 286 | *Triticum aestivum* L. | KU-3416 | The former German Democratic Republic | - | 0.002 | 0.998 |
| 287 | *Triticum aestivum* L. | KU-3417 | The former German Democratic Republic | - | 0.0027 | 0.9973 |
| 288 | *Triticum aestivum* L. | KU-3421 | The former German Democratic Republic | - | 0.0015 | 0.9985 |
| 289 | *Triticum aestivum* L. | KU-3443 | The former German Democratic Republic | - | 0.002 | 0.998 |
| 290 | *Triticum aestivum* L. | KU-3444 | The former German Democratic Republic | - | 0.0105 | 0.9895 |
| 291 | *Triticum aestivum* L. | KU-3445 | The former German Democratic Republic | - | 0.0012 | 0.9988 |
| 292 | *Triticum aestivum* L. | KU-3752 | Egypt | - | 0.002 | 0.998 |
| 293 | *Triticum aestivum* L. | KU-3777 | Jordan | - | 0.001 | 0.999 |
| 294 | *Triticum aestivum* L. | KU-3778 | Lebanon | - | 0.0037 | 0.9963 |
| 295 | *Triticum aestivum* L. | KU-3780 | Syria | - | 0.0022 | 0.9978 |
| 296 | *Triticum aestivum* L. | KU-3784 | Turkey | - | 0.0054 | 0.9946 |
| 297 | *Triticum aestivum* L. | KU-3789 | Turkey | - | 0.001 | 0.999 |
| 298 | *Triticum aestivum* L. | KU-3801 | Turkey | - | 0.003 | 0.997 |
| 299 | *Triticum aestivum* L. | KU-3806 | Turkey | - | 0.0029 | 0.9971 |
| 300 | *Triticum aestivum* L. | KU-3818 | Turkey | - | 0.0032 | 0.9968 |
| 301 | *Triticum aestivum* L. | KU-3834 | Turkey | - | 0.0039 | 0.9961 |
| 302 | *Triticum aestivum* L. | KU-3848 | Turkey | - | 0.003 | 0.997 |
| 303 | *Triticum aestivum* L. | KU-3851 | Turkey | - | 0.002 | 0.998 |
| 304 | *Triticum aestivum* L. | KU-3857 | Turkey | - | 0.0099 | 0.9901 |
| 305 | *Triticum aestivum* L. | KU-3860 | Turkey | - | 0.0246 | 0.9754 |
| 306 | *Triticum aestivum* L. | KU-3868 | Italy | - | 0.0021 | 0.9979 |
| 307 | *Triticum aestivum* L. | KU-4703 | Nepal | - | 0.0649 | 0.9351 |
| 308 | *Triticum aestivum* L. | KU-4714 | Nepal | - | 0.0065 | 0.9935 |
| 309 | *Triticum aestivum* L. | KU-4734 | Nepal | - | 0.001 | 0.999 |
| 310 | *Triticum aestivum* L. | KU-4759 | Nepal | - | 0.0022 | 0.9978 |
| 311 | *Triticum aestivum* L. | KU-4769 | Nepal | - | 0.0139 | 0.9861 |
| 312 | *Triticum aestivum* L. | KU-4783 | Nepal | - | 0.001 | 0.999 |
| 313 | *Triticum aestivum* L. | KU-7001 | Bhutan | - | 0.0027 | 0.9973 |
| 314 | *Triticum aestivum* L. | KU-7041 | Bhutan | - | 0.0033 | 0.9967 |
| 315 | *Triticum aestivum* L. | KU-7113 | Bhutan | - | 0.001 | 0.999 |
| 316 | *Triticum aestivum* L. | KU-7180 | Bhutan | - | 0.001 | 0.999 |
| 317 | *Triticum aestivum* L. | KU-7350 | Turkey | - | 0.0016 | 0.9984 |
| 318 | *Triticum aestivum* L. | KU-7356 | Ethiopia | - | 0.0039 | 0.9961 |
| 319 | *Triticum aestivum* L. | KU-7379 | Ethiopia | - | 0.0017 | 0.9983 |
| 320 | *Triticum aestivum* L. | KU-7406 | Ethiopia | - | 0.0023 | 0.9977 |
| 321 | *Triticum aestivum* L. | KU-7437 | Afghanistan | - | 0.0026 | 0.9974 |
| 322 | *Triticum aestivum* L. | KU-7459 | Afghanistan | - | 0.001 | 0.999 |
| 323 | *Triticum aestivum* L. | KU-7480 | Afghanistan | - | 0.004 | 0.996 |
| 324 | *Triticum aestivum* L. | KU-7624 | Afghanistan | - | 0.002 | 0.998 |
| 325 | *Triticum aestivum* L. | KU-7653 | Afghanistan | - | 0.002 | 0.998 |
| 326 | *Triticum aestivum* L. | KU-7669 | Afghanistan | - | 0.0177 | 0.9823 |
| 327 | *Triticum aestivum* L. | KU-9431 | Ethiopia | - | 0.0012 | 0.9988 |
| 328 | *Triticum aestivum* L. | KU-9460 | Ethiopia | - | 0.001 | 0.999 |
| 329 | *Triticum aestivum* L. | KU-9797 | Ethiopia | - | 0.001 | 0.999 |
| 330 | *Triticum aestivum* L. | KU-9820 | Ethiopia | - | 0.002 | 0.998 |
| 331 | *Triticum aestivum* L. | KU-9867 | Ethiopia | - | 0.001 | 0.999 |
| 332 | *Triticum aestivum* L. | KU-9873 | Ethiopia | - | 0.4897 | 0.5103 |
| 333 | *Triticum aestivum* L. | KU-10001 | Iraq | - | 0.005 | 0.995 |
| 334 | *Triticum aestivum* L. | KU-10154 | Iraq | - | 0.0019 | 0.9981 |
| 335 | *Triticum aestivum* L. | KU-10393 | Iran | - | 0.0031 | 0.9969 |
| 336 | *Triticum aestivum* L. | KU-10439 | Iran | - | 0.0218 | 0.9782 |
| 337 | *Triticum aestivum* L. | KU-10480 | Iran | - | 0.002 | 0.998 |
| 338 | *Triticum aestivum* L. | KU-10510 | Iran | - | 0.05 | 0.95 |
| 339 | *Triticum aestivum* L. | KU-11201 | Afghanistan | - | 0.0021 | 0.9979 |
| 340 | *Triticum aestivum* L. | KU-11214 | Afghanistan | - | 0.0018 | 0.9982 |
| 341 | *Triticum aestivum* L. | KU-11240A | Afghanistan | - | 0.0033 | 0.9967 |
| 342 | *Triticum aestivum* L. | KU-11351 | Romania | - | 0.003 | 0.997 |
| 343 | *Triticum aestivum* L. | KU-11702 | Greece | - | 0.0019 | 0.9981 |
| 344 | *Triticum aestivum* L. | KU-11809 | Greece | - | 0.0017 | 0.9983 |
| 345 | *Triticum aestivum* L. | KU-11829 | Greece | - | 0.0366 | 0.9634 |
| 346 | *Triticum aestivum* L. | KU-13501 | China | - | 0.002 | 0.998 |
| 347 | *Triticum aestivum* L. | KU-13506 | China | - | 0.0031 | 0.9969 |
| 348 | *Triticum aestivum* L. | KU-13546 | China | - | 0.003 | 0.997 |
| 349 | *Triticum aestivum* L. | KU-13631 | China | - | 0.002 | 0.998 |
| 350 | *Triticum aestivum* L. | KU-13662 | China | - | 0.001 | 0.999 |
| 351 | *Triticum aestivum* L. | KU-13708 | China | - | 0.0017 | 0.9983 |
| 352 | *Triticum aestivum* L. | KU-13807 | China | - | 0.0115 | 0.9885 |
| 353 | *Triticum aestivum* L. | KU-13891 | China | - | 0.002 | 0.998 |
| 354 | *Triticum aestivum* L. | Abukumawase (winter type) | Japan | - | 0.001 | 0.999 |
| 355 | *Triticum aestivum* L. | Akadaruma | Japan | - | 0.0023 | 0.9977 |
| 356 | *Triticum aestivum* L. | Ayahikari | Japan | - | 0.002 | 0.998 |
| 357 | *Triticum aestivum* L. | Bobwhite | Mexico | - | 0.0017 | 0.9983 |
| 358 | *Triticum aestivum* L. | Cheyenne | United States of America | - | 0.0152 | 0.9848 |
| 359 | *Triticum aestivum* L. | Chihokukomugi | Japan | - | 0.0033 | 0.9967 |
| 360 | *Triticum aestivum* L. | Chikugoizumi | Japan | - | 0.001 | 0.999 |
| 361 | *Triticum aestivum* L. | Chinese Spring | China | - | 0.001 | 0.999 |
| 362 | *Triticum aestivum* L. | Chogokuwase | Japan | - | 0.002 | 0.998 |
| 363 | *Triticum aestivum* L. | Fujimikomugi | Japan | - | 0.0014 | 0.9986 |
| 364 | *Triticum aestivum* L. | Gamenya | Autralia | - | 0.1728 | 0.8272 |
| 365 | *Triticum aestivum* L. | Hanamanten | Japan | - | 0.001 | 0.999 |
| 366 | *Triticum aestivum* L. | Haruyokoi | Japan | - | 0.003 | 0.997 |
| 367 | *Triticum aestivum* L. | Hokkai 240 | Japan | - | 0.001 | 0.999 |
| 368 | *Triticum aestivum* L. | Hope | United States of America | - | 0.0151 | 0.9849 |
| 369 | *Triticum aestivum* L. | Iwainodaichi | Japan | - | 0.002 | 0.998 |
| 370 | *Triticum aestivum* L. | Kanto 107 | Japan | - | 0.001 | 0.999 |
| 371 | *Triticum aestivum* L. | Kinuiroha | Japan | - | 0.001 | 0.999 |
| 372 | *Triticum aestivum* L. | Kitakamikomugi | Japan | - | 0.0042 | 0.9958 |
| 373 | *Triticum aestivum* L. | Kitanokaori | Japan | - | 0.0018 | 0.9982 |
| 374 | *Triticum aestivum* L. | KS831987 | United States of America | - | 0.001 | 0.999 |
| 375 | *Triticum aestivum* L. | Minaminokaori | Japan | - | 0.0012 | 0.9988 |
| 376 | *Triticum aestivum* L. | Minaminokomugi | Japan | - | 0.001 | 0.999 |
| 377 | *Triticum aestivum* L. | Nambukomugi | Japan | - | 0.0012 | 0.9988 |
| 378 | *Triticum aestivum* L. | Nebarigoshi | Japan | - | 0.001 | 0.999 |
| 379 | *Triticum aestivum* L. | Nishikazekomugi | Japan | - | 0.0014 | 0.9986 |
| 380 | *Triticum aestivum* L. | Nobeokabozykomugi | Japan | - | 0.0019 | 0.9981 |
| 381 | *Triticum aestivum* L. | Norin 26 | Japan | - | 0.001 | 0.999 |
| 382 | *Triticum aestivum* L. | Norin 61 | Japan | - | 0.001 | 0.999 |
| 383 | *Triticum aestivum* L. | Opata 85 | Mexico | - | 0.002 | 0.998 |
| 384 | *Triticum aestivum* L. | Saikai 165 | Japan | - | 0.001 | 0.999 |
| 385 | *Triticum aestivum* L. | Saikai 193 | Japan | - | 0.0039 | 0.9961 |
| 386 | *Triticum aestivum* L. | Shiroganekomugi | Japan | - | 0.0014 | 0.9986 |
| 387 | *Triticum aestivum* L. | Shyunyou | Japan | - | 0.002 | 0.998 |
| 388 | *Triticum aestivum* L. | Sumai #3 | China | - | 0.001 | 0.999 |
| 389 | *Triticum aestivum* L. | Synthetic W7984 | - | - | 0.3477 | 0.6523 |
| 390 | *Triticum aestivum* L. | Tamaizumi | Japan | - | 0.001 | 0.999 |
| 391 | *Triticum aestivum* L. | Timstein | United States of America | - | 0.0061 | 0.9939 |
| 392 | *Triticum aestivum* L. | U24 | China | - | 0.001 | 0.999 |
| 393 | *Triticum aestivum* L. | Variety duhamerianum | - | - | 0.001 | 0.999 |
| 394 | *Triticum aestivum* L. | Zenkojikomugi | Japan | - | 0.0036 | 0.9964 |
