# Supplementary material for: Genetic Basis for Spontaneous Hybrid Genome Doubling during Allopolyploid Speciation of Common Wheat Shown by Natural Variation Analyses of the Paternal Species
Source: PLoS One. 2013 Aug 8;8(8):e68310. doi: 10.1371/journal.pone.0068310 (PMC3738567; doi:10.1371/journal.pone.0068310)
Supplement: Table S4 — The STRUCTURE membership coefficients of the T. aestivum accessions excluding W7984 (K=2). The lineage classification of the accessions is provided. A hyphen indicates that the information is not available. (DOCX) [file pone.0068310.s006.docx]

Table S4. The STRUCTURE membership coefficients of the *T. aestivum* accessions excluding W7984 (*K*=2).

| No. | Species | Accession | Origin | Genepool1 | Genepool2 | Lineage |
| --- | --- | --- | --- | --- | --- | --- |
| 1 | *Triticum aestivum* L. | KU-161 | - | 0.9601 | 0.0399 | AesL1 |
| 2 | *Triticum aestivum* L. | KU-162-2 | Pakistan | 0.9485 | 0.0515 | AesL1 |
| 3 | *Triticum aestivum* L. | KU-166 | China | 0.9197 | 0.0803 | AesL1 |
| 4 | *Triticum aestivum* L. | KU-405 | The former Union of Soviet Socialist Republics | 0.9263 | 0.0737 | AesL1 |
| 5 | *Triticum aestivum* L. | KU-504 | China | 0.9652 | 0.0348 | AesL1 |
| 6 | *Triticum aestivum* L. | KU-601 | Japan | 0.9268 | 0.0732 | AesL1 |
| 7 | *Triticum aestivum* L. | KU-1230 | Japan | 0.943 | 0.057 | AesL1 |
| 8 | *Triticum aestivum* L. | KU-3004 | Pakistan | 0.9667 | 0.0333 | AesL1 |
| 9 | *Triticum aestivum* L. | KU-3037 | Pakistan | 0.9273 | 0.0727 | AesL1 |
| 10 | *Triticum aestivum* L. | KU-3062 | Afghanistan | 0.9732 | 0.0268 | AesL1 |
| 11 | *Triticum aestivum* L. | KU-3097 | Iran | 0.9459 | 0.0541 | AesL1 |
| 12 | *Triticum aestivum* L. | KU-3098 | Iran | 0.9113 | 0.0887 | AesL1 |
| 13 | *Triticum aestivum* L. | KU-3126 | Iran | 0.902 | 0.098 | AesL1 |
| 14 | *Triticum aestivum* L. | KU-3136 | Iran | 0.9493 | 0.0507 | AesL1 |
| 15 | *Triticum aestivum* L. | KU-3162 | Iran | 0.971 | 0.029 | AesL1 |
| 16 | *Triticum aestivum* L. | KU-3189 | Iran | 0.9342 | 0.0658 | AesL1 |
| 17 | *Triticum aestivum* L. | KU-3202 | Iran | 0.9694 | 0.0306 | AesL1 |
| 18 | *Triticum aestivum* L. | KU-3299 | Pakistan | 0.9484 | 0.0516 | AesL1 |
| 19 | *Triticum aestivum* L. | KU-3351 | Pakistan | 0.9605 | 0.0395 | AesL1 |
| 20 | *Triticum aestivum* L. | KU-3752 | Egypt | 0.9709 | 0.0291 | AesL1 |
| 21 | *Triticum aestivum* L. | KU-4734 | Nepal | 0.9789 | 0.0211 | AesL1 |
| 22 | *Triticum aestivum* L. | KU-4759 | Nepal | 0.9699 | 0.0301 | AesL1 |
| 23 | *Triticum aestivum* L. | KU-4769 | Nepal | 0.9595 | 0.0405 | AesL1 |
| 24 | *Triticum aestivum* L. | KU-4783 | Nepal | 0.9673 | 0.0327 | AesL1 |
| 25 | *Triticum aestivum* L. | KU-7001 | Bhutan | 0.9153 | 0.0847 | AesL1 |
| 26 | *Triticum aestivum* L. | KU-7041 | Bhutan | 0.9459 | 0.0541 | AesL1 |
| 27 | *Triticum aestivum* L. | KU-7180 | Bhutan | 0.9269 | 0.0731 | AesL1 |
| 28 | *Triticum aestivum* L. | KU-7350 | Turkey | 0.9185 | 0.0815 | AesL1 |
| 29 | *Triticum aestivum* L. | KU-7459 | Afghanistan | 0.9344 | 0.0656 | AesL1 |
| 30 | *Triticum aestivum* L. | KU-7480 | Afghanistan | 0.9493 | 0.0507 | AesL1 |
| 31 | *Triticum aestivum* L. | KU-9431 | Ethiopia | 0.9028 | 0.0972 | AesL1 |
| 32 | *Triticum aestivum* L. | KU-11240A | Afghanistan | 0.9556 | 0.0444 | AesL1 |
| 33 | *Triticum aestivum* L. | KU-13546 | China | 0.9673 | 0.0327 | AesL1 |
| 34 | *Triticum aestivum* L. | KU-13662 | China | 0.9503 | 0.0497 | AesL1 |
| 35 | *Triticum aestivum* L. | KU-13708 | China | 0.9677 | 0.0323 | AesL1 |
| 36 | *Triticum aestivum* L. | KU-13891 | China | 0.9456 | 0.0544 | AesL1 |
| 37 | *Triticum aestivum* L. | Akadaruma | Japan | 0.9493 | 0.0507 | AesL1 |
| 38 | *Triticum aestivum* L. | Chinese Spring | China | 0.9338 | 0.0662 | AesL1 |
| 39 | *Triticum aestivum* L. | Iwainodaichi | Japan | 0.9599 | 0.0401 | AesL1 |
| 40 | *Triticum aestivum* L. | Kinuiroha | Japan | 0.9406 | 0.0594 | AesL1 |
| 41 | *Triticum aestivum* L. | Minaminokomugi | Japan | 0.9019 | 0.0981 | AesL1 |
| 42 | *Triticum aestivum* L. | Nishikazekomugi | Japan | 0.9577 | 0.0423 | AesL1 |
| 43 | *Triticum aestivum* L. | Nobeokabozykomugi | Japan | 0.9675 | 0.0325 | AesL1 |
| 44 | *Triticum aestivum* L. | Norin 61 | Japan | 0.9402 | 0.0598 | AesL1 |
| 45 | *Triticum aestivum* L. | Saikai 193 | Japan | 0.9526 | 0.0474 | AesL1 |
| 46 | *Triticum aestivum* L. | KU-309 | United States of America | 0.0542 | 0.9458 | AesL2 |
| 47 | *Triticum aestivum* L. | KU-336 | United States of America | 0.0417 | 0.9583 | AesL2 |
| 48 | *Triticum aestivum* L. | KU-366 | United Kingdom | 0.033 | 0.967 | AesL2 |
| 49 | *Triticum aestivum* L. | KU-370 | United Kingdom | 0.0618 | 0.9382 | AesL2 |
| 50 | *Triticum aestivum* L. | KU-371 | United Kingdom | 0.045 | 0.955 | AesL2 |
| 51 | *Triticum aestivum* L. | KU-372 | United Kingdom | 0.0581 | 0.9419 | AesL2 |
| 52 | *Triticum aestivum* L. | KU-373 | United Kingdom | 0.0225 | 0.9775 | AesL2 |
| 53 | *Triticum aestivum* L. | KU-374 | United Kingdom | 0.0695 | 0.9305 | AesL2 |
| 54 | *Triticum aestivum* L. | KU-1002 | Spain | 0.0492 | 0.9508 | AesL2 |
| 55 | *Triticum aestivum* L. | KU-1421 | Romania | 0.0358 | 0.9642 | AesL2 |
| 56 | *Triticum aestivum* L. | KU-1424 | Romania | 0.0356 | 0.9644 | AesL2 |
| 57 | *Triticum aestivum* L. | KU-1668 | The former Union of Soviet Socialist Republics | 0.0477 | 0.9523 | AesL2 |
| 58 | *Triticum aestivum* L. | KU-1797 | The former Union of Soviet Socialist Republics | 0.0613 | 0.9387 | AesL2 |
| 59 | *Triticum aestivum* L. | KU-3784 | Turkey | 0.0615 | 0.9385 | AesL2 |
| 60 | *Triticum aestivum* L. | KU-3801 | Turkey | 0.055 | 0.945 | AesL2 |
| 61 | *Triticum aestivum* L. | KU-3806 | Turkey | 0.0502 | 0.9498 | AesL2 |
| 62 | *Triticum aestivum* L. | KU-3857 | Turkey | 0.0434 | 0.9566 | AesL2 |
| 63 | *Triticum aestivum* L. | Cheyenne | United States of America | 0.0372 | 0.9628 | AesL2 |
| 64 | *Triticum aestivum* L. | Hope | United States of America | 0.0681 | 0.9319 | AesL2 |
| 65 | *Triticum aestivum* L. | KU-152 | China | 0.1752 | 0.8248 | Admixed |
| 66 | *Triticum aestivum* L. | KU-192 | - | 0.818 | 0.182 | Admixed |
| 67 | *Triticum aestivum* L. | KU-197 | Turkey | 0.5118 | 0.4882 | Admixed |
| 68 | *Triticum aestivum* L. | KU-265 | Japan | 0.494 | 0.506 | Admixed |
| 69 | *Triticum aestivum* L. | KU-333 | Canada | 0.3586 | 0.6414 | Admixed |
| 70 | *Triticum aestivum* L. | KU-479 | China | 0.8659 | 0.1341 | Admixed |
| 71 | *Triticum aestivum* L. | KU-481 | China | 0.5445 | 0.4555 | Admixed |
| 72 | *Triticum aestivum* L. | KU-483 | Tanzania | 0.74 | 0.26 | Admixed |
| 73 | *Triticum aestivum* L. | KU-497 | India | 0.4034 | 0.5966 | Admixed |
| 74 | *Triticum aestivum* L. | KU-1005 | Spain | 0.3212 | 0.6788 | Admixed |
| 75 | *Triticum aestivum* L. | KU-1011 | Spain | 0.2523 | 0.7477 | Admixed |
| 76 | *Triticum aestivum* L. | KU-1020 | Spain | 0.3284 | 0.6716 | Admixed |
| 77 | *Triticum aestivum* L. | KU-1049 | Spain | 0.1767 | 0.8233 | Admixed |
| 78 | *Triticum aestivum* L. | KU-1062 | Spain | 0.5348 | 0.4652 | Admixed |
| 79 | *Triticum aestivum* L. | KU-1137 | Spain | 0.5612 | 0.4388 | Admixed |
| 80 | *Triticum aestivum* L. | KU-1143 | Spain | 0.5448 | 0.4552 | Admixed |
| 81 | *Triticum aestivum* L. | KU-1208 | Japan | 0.8769 | 0.1231 | Admixed |
| 82 | *Triticum aestivum* L. | KU-1215 | Japan | 0.3575 | 0.6425 | Admixed |
| 83 | *Triticum aestivum* L. | KU-1279 | Japan | 0.6599 | 0.3401 | Admixed |
| 84 | *Triticum aestivum* L. | KU-1302 | Greece | 0.1082 | 0.8918 | Admixed |
| 85 | *Triticum aestivum* L. | KU-1347 | Greece | 0.2486 | 0.7514 | Admixed |
| 86 | *Triticum aestivum* L. | KU-1392 | Romania | 0.1306 | 0.8694 | Admixed |
| 87 | *Triticum aestivum* L. | KU-1394 | Romania | 0.1255 | 0.8745 | Admixed |
| 88 | *Triticum aestivum* L. | KU-1521 | The former Union of Soviet Socialist Republics | 0.1079 | 0.8921 | Admixed |
| 89 | *Triticum aestivum* L. | KU-1527 | The former Union of Soviet Socialist Republics | 0.1523 | 0.8477 | Admixed |
| 90 | *Triticum aestivum* L. | KU-1644 | The former Union of Soviet Socialist Republics | 0.6795 | 0.3205 | Admixed |
| 91 | *Triticum aestivum* L. | KU-1697 | The former Union of Soviet Socialist Republics | 0.1009 | 0.8991 | Admixed |
| 92 | *Triticum aestivum* L. | KU-1812 | Georgia | 0.5677 | 0.4323 | Admixed |
| 93 | *Triticum aestivum* L. | KU-1814 | Georgia | 0.5252 | 0.4748 | Admixed |
| 94 | *Triticum aestivum* L. | KU-1817 | Georgia | 0.5842 | 0.4158 | Admixed |
| 95 | *Triticum aestivum* L. | KU-3006 | Pakistan | 0.7463 | 0.2537 | Admixed |
| 96 | *Triticum aestivum* L. | KU-3010 | Pakistan | 0.8957 | 0.1043 | Admixed |
| 97 | *Triticum aestivum* L. | KU-3045 | Afghanistan | 0.6081 | 0.3919 | Admixed |
| 98 | *Triticum aestivum* L. | KU-3054 | Afghanistan | 0.7984 | 0.2016 | Admixed |
| 99 | *Triticum aestivum* L. | KU-3063 | Afganistan | 0.5894 | 0.4106 | Admixed |
| 100 | *Triticum aestivum* L. | KU-3083 | Afghanistan | 0.4391 | 0.5609 | Admixed |
| 101 | *Triticum aestivum* L. | KU-3089 | Afghanistan | 0.7831 | 0.2169 | Admixed |
| 102 | *Triticum aestivum* L. | KU-3121 | Iran | 0.8996 | 0.1004 | Admixed |
| 103 | *Triticum aestivum* L. | KU-3184 | Iran | 0.8554 | 0.1446 | Admixed |
| 104 | *Triticum aestivum* L. | KU-3232 | Iran | 0.8475 | 0.1525 | Admixed |
| 105 | *Triticum aestivum* L. | KU-3236 | Iran | 0.4131 | 0.5869 | Admixed |
| 106 | *Triticum aestivum* L. | KU-3242 | Iran | 0.5032 | 0.4968 | Admixed |
| 107 | *Triticum aestivum* L. | KU-3274 | Iran | 0.7671 | 0.2329 | Admixed |
| 108 | *Triticum aestivum* L. | KU-3289 | Iran | 0.7378 | 0.2622 | Admixed |
| 109 | *Triticum aestivum* L. | KU-3377 | Iran | 0.8826 | 0.1174 | Admixed |
| 110 | *Triticum aestivum* L. | KU-3401 | The former German Democratic Republic | 0.5116 | 0.4884 | Admixed |
| 111 | *Triticum aestivum* L. | KU-3413 | The former German Democratic Republic | 0.8935 | 0.1065 | Admixed |
| 112 | *Triticum aestivum* L. | KU-3416 | The former German Democratic Republic | 0.512 | 0.488 | Admixed |
| 113 | *Triticum aestivum* L. | KU-3417 | The former German Democratic Republic | 0.6695 | 0.3305 | Admixed |
| 114 | *Triticum aestivum* L. | KU-3421 | The former German Democratic Republic | 0.6002 | 0.3998 | Admixed |
| 115 | *Triticum aestivum* L. | KU-3443 | The former German Democratic Republic | 0.5871 | 0.4129 | Admixed |
| 116 | *Triticum aestivum* L. | KU-3444 | The former German Democratic Republic | 0.4457 | 0.5543 | Admixed |
| 117 | *Triticum aestivum* L. | KU-3445 | The former German Democratic Republic | 0.7969 | 0.2031 | Admixed |
| 118 | *Triticum aestivum* L. | KU-3777 | Jordan | 0.2322 | 0.7678 | Admixed |
| 119 | *Triticum aestivum* L. | KU-3778 | Lebanon | 0.4062 | 0.5938 | Admixed |
| 120 | *Triticum aestivum* L. | KU-3780 | Syria | 0.3957 | 0.6043 | Admixed |
| 121 | *Triticum aestivum* L. | KU-3789 | Turkey | 0.5435 | 0.4565 | Admixed |
| 122 | *Triticum aestivum* L. | KU-3818 | Turkey | 0.1519 | 0.8481 | Admixed |
| 123 | *Triticum aestivum* L. | KU-3834 | Turkey | 0.2211 | 0.7789 | Admixed |
| 124 | *Triticum aestivum* L. | KU-3848 | Turkey | 0.8382 | 0.1618 | Admixed |
| 125 | *Triticum aestivum* L. | KU-3851 | Turkey | 0.1353 | 0.8647 | Admixed |
| 126 | *Triticum aestivum* L. | KU-3860 | Turkey | 0.2969 | 0.7031 | Admixed |
| 127 | *Triticum aestivum* L. | KU-3868 | Italy | 0.3775 | 0.6225 | Admixed |
| 128 | *Triticum aestivum* L. | KU-4703 | Nepal | 0.367 | 0.633 | Admixed |
| 129 | *Triticum aestivum* L. | KU-4714 | Nepal | 0.888 | 0.112 | Admixed |
| 130 | *Triticum aestivum* L. | KU-7113 | Bhutan | 0.8917 | 0.1083 | Admixed |
| 131 | *Triticum aestivum* L. | KU-7356 | Ethiopia | 0.2101 | 0.7899 | Admixed |
| 132 | *Triticum aestivum* L. | KU-7379 | Ethiopia | 0.459 | 0.541 | Admixed |
| 133 | *Triticum aestivum* L. | KU-7406 | Ethiopia | 0.556 | 0.444 | Admixed |
| 134 | *Triticum aestivum* L. | KU-7437 | Afghanistan | 0.801 | 0.199 | Admixed |
| 135 | *Triticum aestivum* L. | KU-7624 | Afghanistan | 0.6636 | 0.3364 | Admixed |
| 136 | *Triticum aestivum* L. | KU-7653 | Afghanistan | 0.6452 | 0.3548 | Admixed |
| 137 | *Triticum aestivum* L. | KU-7669 | Afghanistan | 0.8527 | 0.1473 | Admixed |
| 138 | *Triticum aestivum* L. | KU-9460 | Ethiopia | 0.5096 | 0.4904 | Admixed |
| 139 | *Triticum aestivum* L. | KU-9797 | Ethiopia | 0.7288 | 0.2712 | Admixed |
| 140 | *Triticum aestivum* L. | KU-9820 | Ethiopia | 0.1998 | 0.8002 | Admixed |
| 141 | *Triticum aestivum* L. | KU-9867 | Ethiopia | 0.4325 | 0.5675 | Admixed |
| 142 | *Triticum aestivum* L. | KU-9873 | Ethiopia | 0.2095 | 0.7905 | Admixed |
| 143 | *Triticum aestivum* L. | KU-10001 | Iraq | 0.209 | 0.791 | Admixed |
| 144 | *Triticum aestivum* L. | KU-10154 | Iraq | 0.6438 | 0.3562 | Admixed |
| 145 | *Triticum aestivum* L. | KU-10393 | Iran | 0.6244 | 0.3756 | Admixed |
| 146 | *Triticum aestivum* L. | KU-10439 | Iran | 0.8824 | 0.1176 | Admixed |
| 147 | *Triticum aestivum* L. | KU-10480 | Iran | 0.8292 | 0.1708 | Admixed |
| 148 | *Triticum aestivum* L. | KU-10510 | Iran | 0.5688 | 0.4312 | Admixed |
| 149 | *Triticum aestivum* L. | KU-11201 | Afghanistan | 0.6016 | 0.3984 | Admixed |
| 150 | *Triticum aestivum* L. | KU-11214 | Afghanistan | 0.8758 | 0.1242 | Admixed |
| 151 | *Triticum aestivum* L. | KU-11351 | Romania | 0.4074 | 0.5926 | Admixed |
| 152 | *Triticum aestivum* L. | KU-11702 | Greece | 0.1178 | 0.8822 | Admixed |
| 153 | *Triticum aestivum* L. | KU-11809 | Greece | 0.3837 | 0.6163 | Admixed |
| 154 | *Triticum aestivum* L. | KU-11829 | Greece | 0.1406 | 0.8594 | Admixed |
| 155 | *Triticum aestivum* L. | KU-13501 | China | 0.4885 | 0.5115 | Admixed |
| 156 | *Triticum aestivum* L. | KU-13506 | China | 0.1333 | 0.8667 | Admixed |
| 157 | *Triticum aestivum* L. | KU-13631 | China | 0.8392 | 0.1608 | Admixed |
| 158 | *Triticum aestivum* L. | KU-13807 | China | 0.8396 | 0.1604 | Admixed |
| 159 | *Triticum aestivum* L. | Abukumawase (winter type) | Japan | 0.857 | 0.143 | Admixed |
| 160 | *Triticum aestivum* L. | Ayahikari | Japan | 0.7025 | 0.2975 | Admixed |
| 161 | *Triticum aestivum* L. | Bobwhite | Mexico | 0.4325 | 0.5675 | Admixed |
| 162 | *Triticum aestivum* L. | Chihokukomugi | Japan | 0.1717 | 0.8283 | Admixed |
| 163 | *Triticum aestivum* L. | Chikugoizumi | Japan | 0.8576 | 0.1424 | Admixed |
| 164 | *Triticum aestivum* L. | Chogokuwase | Japan | 0.6585 | 0.3415 | Admixed |
| 165 | *Triticum aestivum* L. | Fujimikomugi | Japan | 0.7554 | 0.2446 | Admixed |
| 166 | *Triticum aestivum* L. | Gamenya | Autralia | 0.5124 | 0.4876 | Admixed |
| 167 | *Triticum aestivum* L. | Hanamanten | Japan | 0.5066 | 0.4934 | Admixed |
| 168 | *Triticum aestivum* L. | Haruyokoi | Japan | 0.4288 | 0.5712 | Admixed |
| 169 | *Triticum aestivum* L. | Hokkai 240 | Japan | 0.536 | 0.464 | Admixed |
| 170 | *Triticum aestivum* L. | Kanto 107 | Japan | 0.5981 | 0.4019 | Admixed |
| 171 | *Triticum aestivum* L. | Kitakamikomugi | Japan | 0.5096 | 0.4904 | Admixed |
| 172 | *Triticum aestivum* L. | Kitanokaori | Japan | 0.4984 | 0.5016 | Admixed |
| 173 | *Triticum aestivum* L. | KS831987 | United States of America | 0.4714 | 0.5286 | Admixed |
| 174 | *Triticum aestivum* L. | Minaminokaori | Japan | 0.7989 | 0.2011 | Admixed |
| 175 | *Triticum aestivum* L. | Nambukomugi | Japan | 0.7246 | 0.2754 | Admixed |
| 176 | *Triticum aestivum* L. | Nebarigoshi | Japan | 0.4748 | 0.5252 | Admixed |
| 177 | *Triticum aestivum* L. | Norin 26 | Japan | 0.8816 | 0.1184 | Admixed |
| 178 | *Triticum aestivum* L. | Opata 85 | Mexico | 0.4864 | 0.5136 | Admixed |
| 179 | *Triticum aestivum* L. | Saikai 165 | Japan | 0.7766 | 0.2234 | Admixed |
| 180 | *Triticum aestivum* L. | Shiroganekomugi | Japan | 0.8955 | 0.1045 | Admixed |
| 181 | *Triticum aestivum* L. | Shyunyou | Japan | 0.425 | 0.575 | Admixed |
| 182 | *Triticum aestivum* L. | Sumai #3 | China | 0.6413 | 0.3587 | Admixed |
| 183 | *Triticum aestivum* L. | Tamaizumi | Japan | 0.7873 | 0.2127 | Admixed |
| 184 | *Triticum aestivum* L. | Timstein | United States of America | 0.4912 | 0.5088 | Admixed |
| 185 | *Triticum aestivum* L. | U24 | China | 0.557 | 0.443 | Admixed |
| 186 | *Triticum aestivum* L. | Variety duhamerianum | - | 0.6293 | 0.3707 | Admixed |
| 187 | *Triticum aestivum* L. | Zenkojikomugi | Japan | 0.5585 | 0.4415 | Admixed |
