# Supplementary material for: Genetic Basis for Spontaneous Hybrid Genome Doubling during Allopolyploid Speciation of Common Wheat Shown by Natural Variation Analyses of the Paternal Species
Source: PLoS One. 2013 Aug 8;8(8):e68310. doi: 10.1371/journal.pone.0068310 (PMC3738567; doi:10.1371/journal.pone.0068310)
Supplement: Table S5 — Genome doubling frequencies of the LDN-Ae.tauschii triploid F1 hybrids. A hyphen indicates that the information is not available. (DOCX) [file pone.0068310.s007.docx]

Table S5. Genome doubling frequencies of the LDN-*Ae. tauschii* triploid F1 hybrids.

| *Ae. tauschii* accession | Lineage | Hybrid genome doubling frequency (2003) | Hybrid genome doubling frequency (2004) |
| --- | --- | --- | --- |
| AE 1090 | TauL1 | 0.39 | 0.54 |
| AT 55 | TauL1 | - | 0.33 |
| AT 76 | TauL1 | - | 0.18 |
| AT 80 | TauL1 | - | 0.34 |
| IG 126387 | TauL1 | - | 0.41 |
| IG 127015 | TauL1 | 0.30 | - |
| IG 131606 | TauL1 | - | 0.47 |
| IG 47259 | TauL1 | 0.23 | - |
| IG 48042 | TauL1 | - | 0.38 |
| KU-2001 | TauL1 | 0.53 | - |
| KU-2012 | TauL1 | - | 0.48 |
| KU-2025 | TauL1 | - | 0.31 |
| KU-2068 | TauL1 | - | 0.13 |
| KU-2132 | TauL1 | 0.50 | - |
| KU-2136 | TauL1 | 0.38 | - |
| KU-2144 | TauL1 | - | 0.43 |
| KU-2816 | TauL1 | 0.63 | 0.61 |
| KU-2826 | TauL1 | - | 0.60 |
| KU-2828 | TauL1 | - | 0.60 |
| PI 476874 | TauL1 | 0.25 | 0.41 |
| PI 486274 | TauL1 | 0.35 | - |
| PI 499262 | TauL1 | 0.27 | - |
| PI 508262 | TauL1 | 0.33 | - |
| IG 47202 | TauL2 | - | 0.35 |
| KU-20-10 | TauL2 | 0.33 | - |
| KU-20-8 | TauL2 | - | 0.16 |
| KU-20-9 | TauL2 | 0.39 | 0.50 |
| KU-2069 | TauL2 | 0.13 | - |
| KU-2075 | TauL2 | - | 0.41 |
| KU-2076 | TauL2 | 0.24 | 0.49 |
| KU-2078 | TauL2 | 0.46 | - |
| KU-2079 | TauL2 | 0.25 | 0.33 |
| KU-2080 | TauL2 | 0.07 | 0.06 |
| KU-2088 | TauL2 | 0.41 | 0.59 |
| KU-2090 | TauL2 | 0.28 | - |
| KU-2091 | TauL2 | 0.29 | - |
| KU-2092 | TauL2 | - | 0.65 |
| KU-2093 | TauL2 | 0.48 | 0.39 |
| KU-2097 | TauL2 | 0.08 | 0.11 |
| KU-2098 | TauL2 | 0.10 | 0.35 |
| KU-2103 | TauL2 | 0.58 | 0.66 |
| KU-2104 | TauL2 | 0.54 | - |
| KU-2105 | TauL2 | - | 0.49 |
| KU-2106 | TauL2 | - | 0.50 |
| KU-2109 | TauL2 | 0.30 | - |
| KU-2111 | TauL2 | - | 0.61 |
| KU-2124 | TauL2 | - | 0.21 |
| KU-2126 | TauL2 | 0.23 | - |
| KU-2155 | TauL2 | 0.18 | - |
| KU-2156 | TauL2 | 0.26 | 0.45 |
| KU-2158 | TauL2 | 0.28 | 0.51 |
| KU-2159 | TauL2 | - | 0.68 |
| KU-2160 | TauL2 | - | 0.47 |
| AE 454 | TauL3 | - | 0.26 |
| AE 929 | TauL3 | - | 0.06 |
| KU-2829A | TauL3 | - | 0.19 |
