# Supplementary material for: Genetic Basis for Spontaneous Hybrid Genome Doubling during Allopolyploid Speciation of Common Wheat Shown by Natural Variation Analyses of the Paternal Species
Source: PLoS One. 2013 Aug 8;8(8):e68310. doi: 10.1371/journal.pone.0068310 (PMC3738567; doi:10.1371/journal.pone.0068310)
Supplement: Table S6 — Comparison of normal and aberrant PMC counts between the HGD and LGD hybrids. (DOCX) [file pone.0068310.s008.docx]

Table S6. Comparison of normal and aberrant PMC counts between the HGD and LGD hybrids

| Stage | Aberration type | HGD hybrid^a^ | LGD hybrid^a^ |
| --- | --- | --- | --- |
| Prophase | Normal | 93 | 102 |
|  | Irregular cytokinesis | 0 | 0 |
|  | Lagging chromosomes | 0 | 0 |
|  | Both aberrations | 0 | 0 |
|  | Total, aberrants | 0 | 0 |
|  | Subtotal | 93 | 102 |
| Late prophase | Normal | 79 | 101 |
|  | Irregular cytokinesis | 0 | 0 |
|  | Lagging chromosomes | 0 | 0 |
|  | Both aberrations | 0 | 0 |
|  | Total, aberrants | 0 | 0 |
|  | Subtotal | 79 | 101 |
| Metaphase | Normal | 54 (69.2) | 28 (45.9) |
|  | Irregular cytokinesis | 0 | 0 |
|  | Lagging chromosomes | 24 (30.8) | 30 (49.2) |
|  | Both aberrations | 0 | 3 (4.9) |
|  | Total, aberrants | 24 (30.8) | 33 (54.1) |
|  | Subtotal | 78 | 61 |
| Restitutive nucleus | Normal | 58 (82.9) | 28 (35.9) |
|  | Irregular cytokinesis | 9 (12.9) | 26 (33.3) |
|  | Lagging chromosomes | 3 (4.3) | 7 (9) |
|  | Both aberrations | 0 | 17 (21.8) |
|  | Total, aberrants | 12 (17.1) | 50 (64.1) |
|  | Subtotal | 70 | 78 |
| Anaphase | Normal | 28 (48.3) | 16 (29.6) |
|  | Irregular cytokinesis | 2 (3.4) | 4 (7.4) |
|  | Lagging chromosomes | 24 (41.4) | 27 (50) |
|  | Both aberrations | 4 (6.9) | 7 (13) |
|  | Total, aberrants | 30 (51.7) | 38 (70.4) |
|  | Subtotal | 58 | 54 |
| Telophase/dyad | Normal | 100 (70.4) | 22 (21.2) |
|  | Irregular cytokinesis | 10 (7.0) | 16 (15.4) |
|  | Lagging chromosomes | 16 (11.3) | 28 (26.9) |
|  | Both aberrations | 16 (11.3) | 38 (36.5) |
|  | Total, aberrants | 42 (29.6) | 82 (78.8) |
|  | Subtotal | 142 | 104 |
| Total |  | 520 | 500 |

^a^ Within-stage percentages are given in parentheses.
