# Supplementary material for: Genetic Basis for Spontaneous Hybrid Genome Doubling during Allopolyploid Speciation of Common Wheat Shown by Natural Variation Analyses of the Paternal Species
Source: PLoS One. 2013 Aug 8;8(8):e68310. doi: 10.1371/journal.pone.0068310 (PMC3738567; doi:10.1371/journal.pone.0068310)
Supplement: Table S7 — Linkages and positions of microsatellite markers used for the QTL analysis of hybrid genome doubling. (DOCX) [file pone.0068310.s009.docx]

Table S7. Linkages and positions of microsatellite markers used for the QTL analysis of hybrid genome doubling.

| Chromosome | Position (centimorgan) | Microsatellite marker |
| --- | --- | --- |
| 1 | 0.0 | *Xwmc432* |
| 1 | 3.3 | *Xcfd61* |
| 1 | 8.9 | *Xcfd92* |
| 1 | 16.9 | *Xcfd72* |
| 1 | 18.8 | *Xwmc429* |
| 1 | 24.7 | *Xwmc216* |
| 1 | 36.2 | *Xcfd48* |
| 1 | 52.4 | *Xgdm126* |
| 1 | 78.8 | *Xgwm232* |
| 1 | 79.2 | *Xwmc609* |
| 1 | 85.3 | *Xgdm111* |
| 2 | 0.0 | *Xgwm455* |
| 2 | 0.4 | *Xgwm296* |
| 2 | 1.9 | *Xwmc503* |
| 2 | 17.3 | *Xbar168* |
| 2 | 34.3 | *Xwmc18* |
| 2 | 36.6 | *Xbar145* |
| 2 | 42.8 | *Xgwm157* |
| 2 | 56.9 | *Xwmc175* |
| 2 | 56.9 | *Xcfd233* |
| 2 | 112.1 | *Xwmc167* |
| 3 | 0.0 | *Xcfd35* |
| 3 | 10.7 | *Xcfd55* |
| 3 | 45.3 | *Xwmc529* |
| 3 | 49.7 | *Xwmc533* |
| 3 | 51.8 | *Xcfd4* |
| 3 | 55.5 | *Xgwm52* |
| 3 | 70.9 | *Xcfd201* |
| 3 | 72.8 | *Xgwm645* |
| 3 | 83.9 | *Xcfd152* |
| 3 | 90.4 | *Xcfd223* |
| 3 | 95.1 | *Xcfd211* |
| 3 | 98.8 | *Xgwm3* |
| 3 | 101.2 | *Xwmc552* |
| 3 | 106.9 | *Xcfd9* |
| 3 | 116.1 | *Xgwm114* |
| 4 | 0.0 | *Xwmc285* |
| 4 | 12.2 | *Xwmc617* |
| 4 | 32.3 | *Xwmc48* |
| 4 | 32.7 | *Xwmc720* |
| 4 | 33.2 | *Xcfd106* |
| 4 | 33.2 | *Xwmc457* |
| 4 | 39.1 | *Xwmc331* |
| 4 | 67.0 | *Xcfd84* |
| 5 | 0.0 | *Xcfd189* |
| 5 | 14.5 | *Xcfd81* |
| 5 | 21.7 | *Xcfd67* |
| 5 | 22.4 | *Xwmc318* |
| 5 | 22.9 | *Xcfd40* |
| 5 | 44.2 | *Xcfd8* |
| 5 | 53.4 | *Xcfd102* |
| 5 | 60.9 | *Xgdm153* |
| 5 | 62.2 | *Xcfd7* |
| 5 | 66.5 | *Xcfd12* |
| 5 | 70.6 | *Xwmc289* |
| 5 | 76.9 | *Xgwm292* |
| 5 | 77.6 | *Xwmc215* |
| 5 | 77.6 | *Xgwm212* |
| 5 | 82.4 | *Xcfd29* |
| 5 | 90.9 | *Xwmc97* |
| 5 | 97.5 | *Xcfd86* |
| 5 | 112.2 | *Xcfd10* |
| 5 | 131.8 | *Xwmc443* |
| 5 | 134.2 | *Xgwm272* |
| 6 | 0.0 | *Xcfd49* |
| 6 | 35.7 | *Xcfd42* |
| 6 | 59.5 | *Xcfd287* |
| 6 | 60.0 | *Xcfd76* |
| 6 | 123.4 | *Xwmc773* |
| 7 | 0.0 | *Xwmc463* |
| 7 | 1.4 | *Xwmc438* |
| 7 | 38.1 | *Xwmc121* |
| 7 | 49.5 | *Xwmc488* |
| 7 | 57.2 | *Xgdm67* |
| 7 | 73.4 | *Xwmc671* |
| 7 | 102.5 | *Xwmc273* |
| 7 | 104.2 | *Xgwm37* |
